# Supplementary material for: Impact of social restrictions during the COVID-19 pandemic on the physical activity levels of adults aged 50–92 years: a baseline survey of the CHARIOT COVID-19 Rapid Response prospective cohort study
Source: BMJ Open. 2021 Aug 25;11(8):e050680. doi: 10.1136/bmjopen-2021-050680 (PMC8390149; doi:10.1136/bmjopen-2021-050680)
Supplement: Supplementary data [file bmjopen-2021-050680supp001.pdf]

Supplementary file 1

Supplementary methods

Metabolic Equivalent of Task (MET) calculation

Briefly, 1 MET equates to an individual's resting energy expenditure. According to the IPAQ scoring protocol, 3.3 METS is considered equivalent to walking, and moderate and vigorous activity to be 4 and 8 METS, respectively. To calculate the continuous variable of total MET minutes a week, the self-reported duration (minutes) and frequency (days) of each of these PA categories is multiplied by the by the specified metric.

For the purposes of regression analyses, employment status was re-categorised as per Table S1

Supplementary figures and tables

Figure S1: Timeline of lockdown restrictions in the United Kingdom in 2020

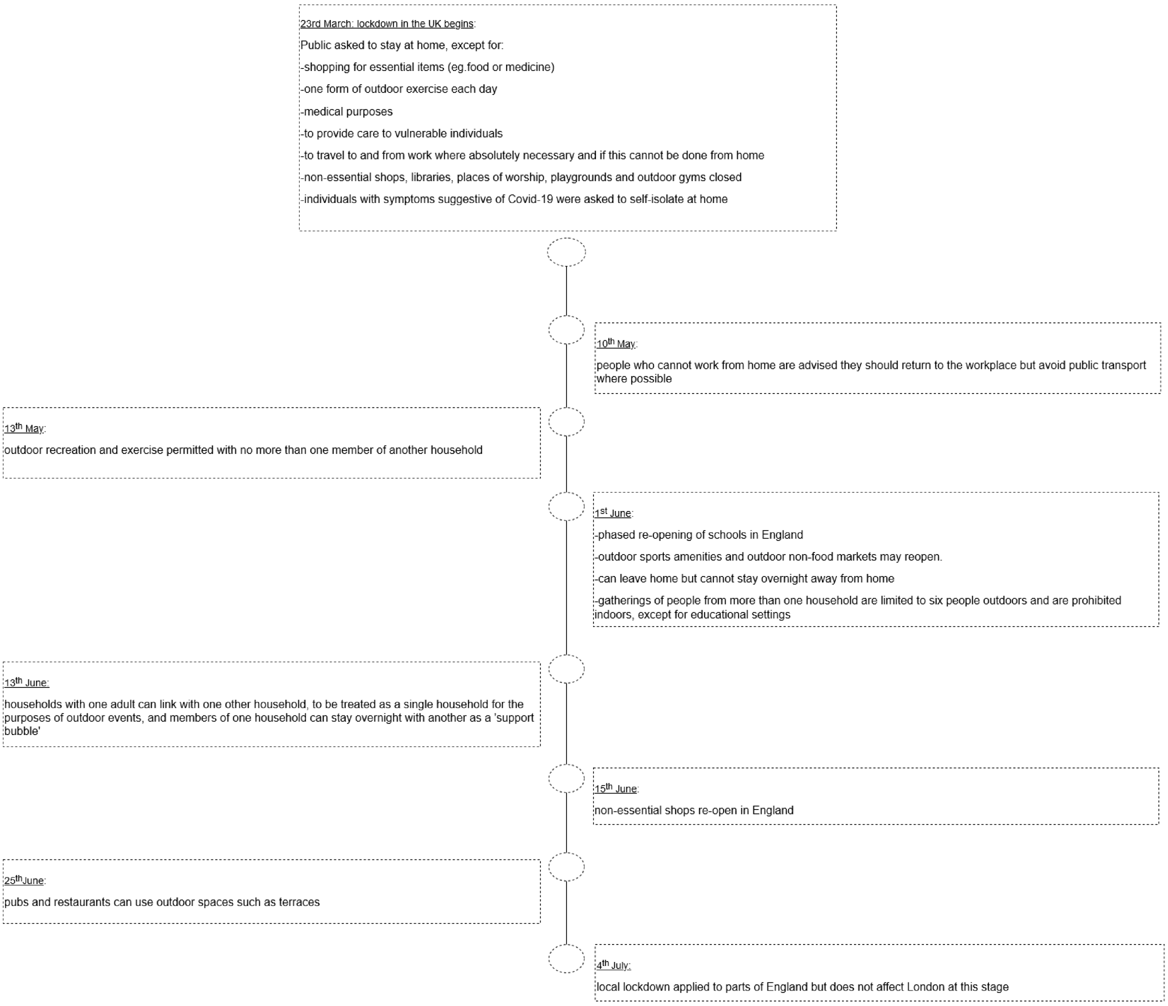

Gov.uk. UK Government COVID-19 guidance

Figure S2: Box-plot of distribution of MET minutes per week after introduction of lockdown by month of survey completion for 6,219 participants with completed IPAQ data

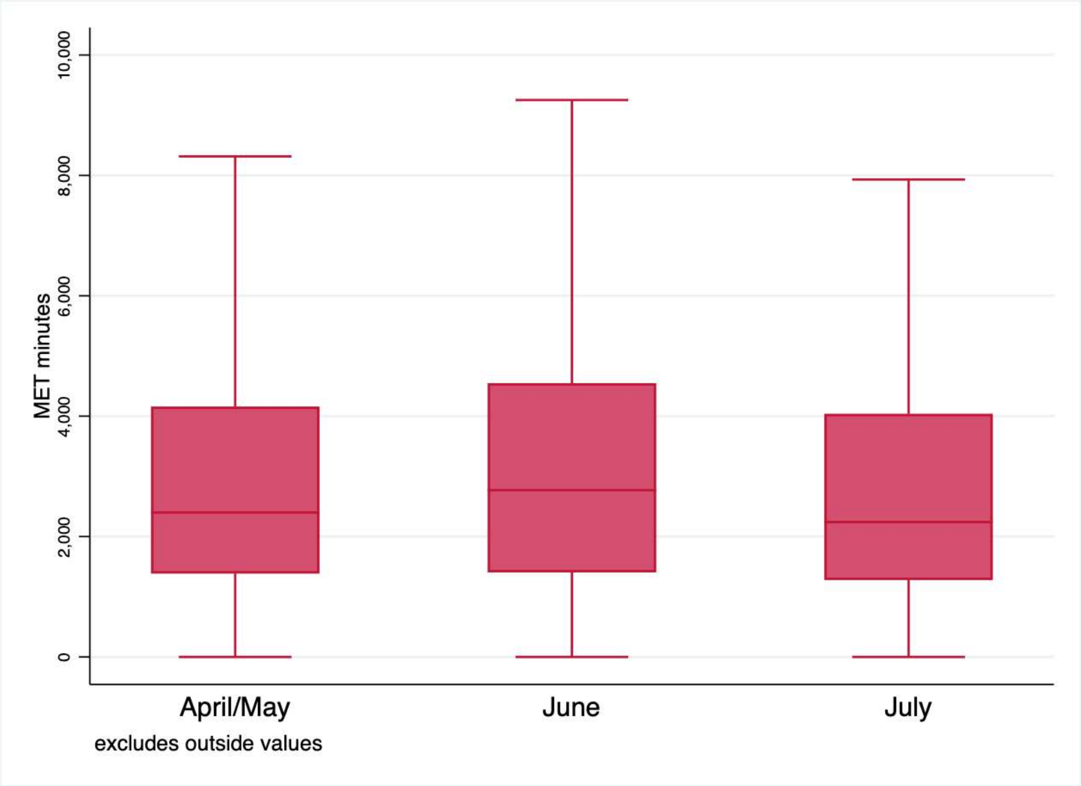

Table S1: Mean MET minutes after introduction of lockdown measures by month of survey completion

Month	Total	Percent	Mean MET minutes	p value¶
April/May*	4975	80.0%	3139	
June	994	16.0%	3470	0.0007
July	250	4.0%	2967	

* April (110) and May (4865) combined due to small numbers completed in April
¶ p-value from linear regression models of MET minutes as dependent variable, against survey completion month as explanatory variable.

Table S2: Re-categorisation of employment status

Recategorised variable	Original variable(s)
Retired	Retired
Employed	A key worker<br>A student<br>Continuing to work in your usual job; at home<br>Continuing to work in your usual job and leave home for your job
Furloughed	Furloughed
Unemployed	Had to close your business due to COVID-19<br>Lost my job due to the lockdown<br>Unemployed
Missing	None of the above

Figure S3: Causal diagram representing factors impacting on change in physical activity after lockdown

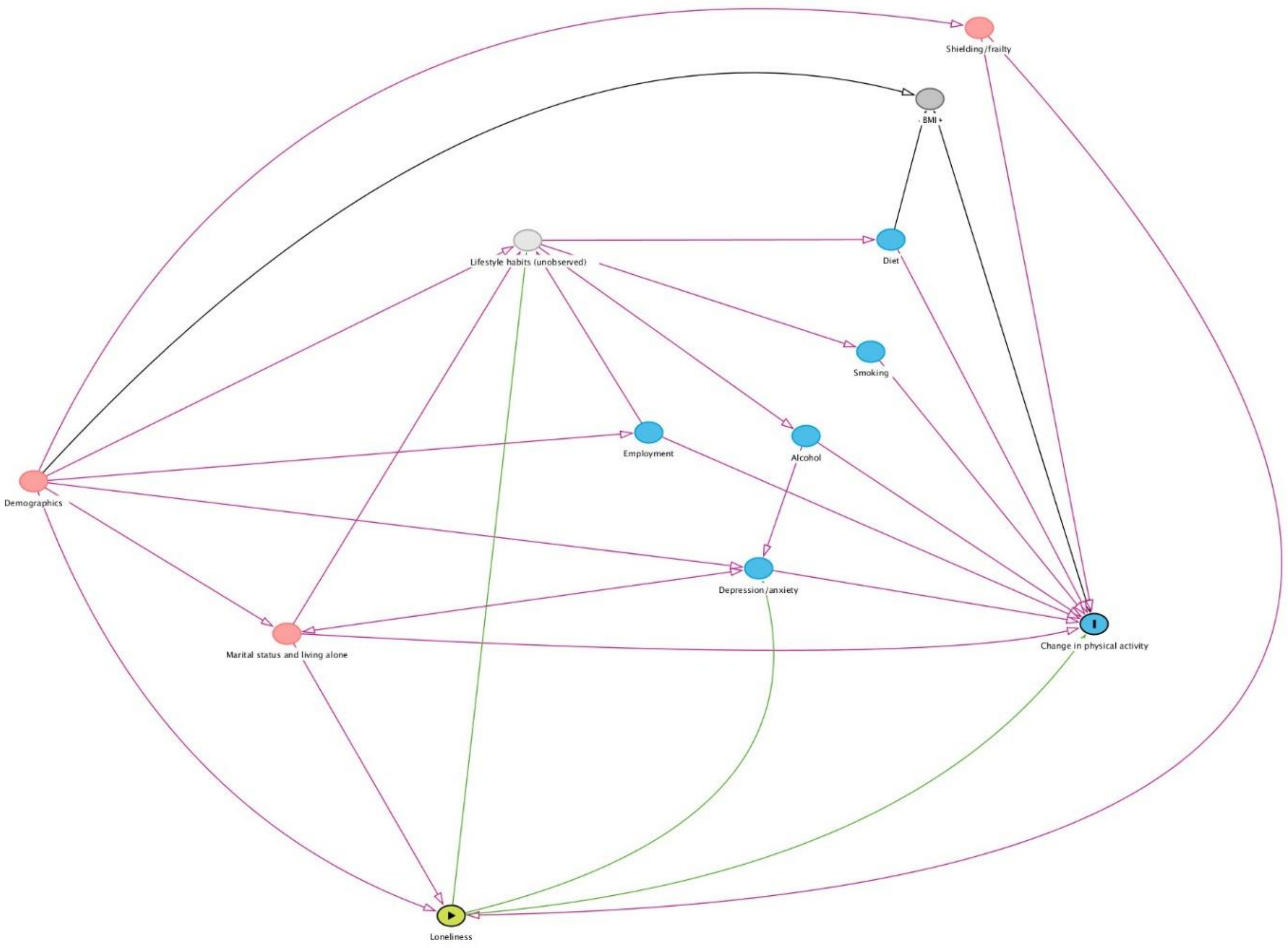

Figure S4: Box-plot of distribution of MET minutes per week before and after the introduction of lockdown for 6,219 participants with completed IPAQ data

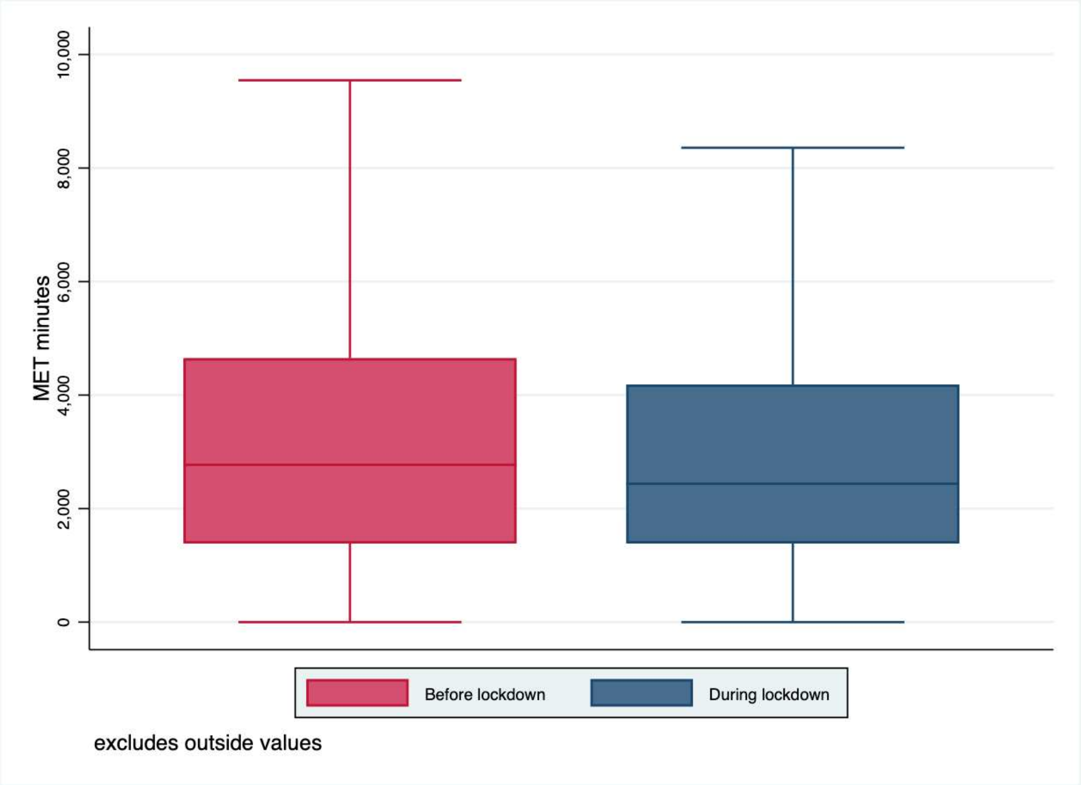

Figure S5: Box-plot of distribution of MET minutes per week before introduction of lockdown by month of survey completion for 6,219 participants with completed IPAQ data

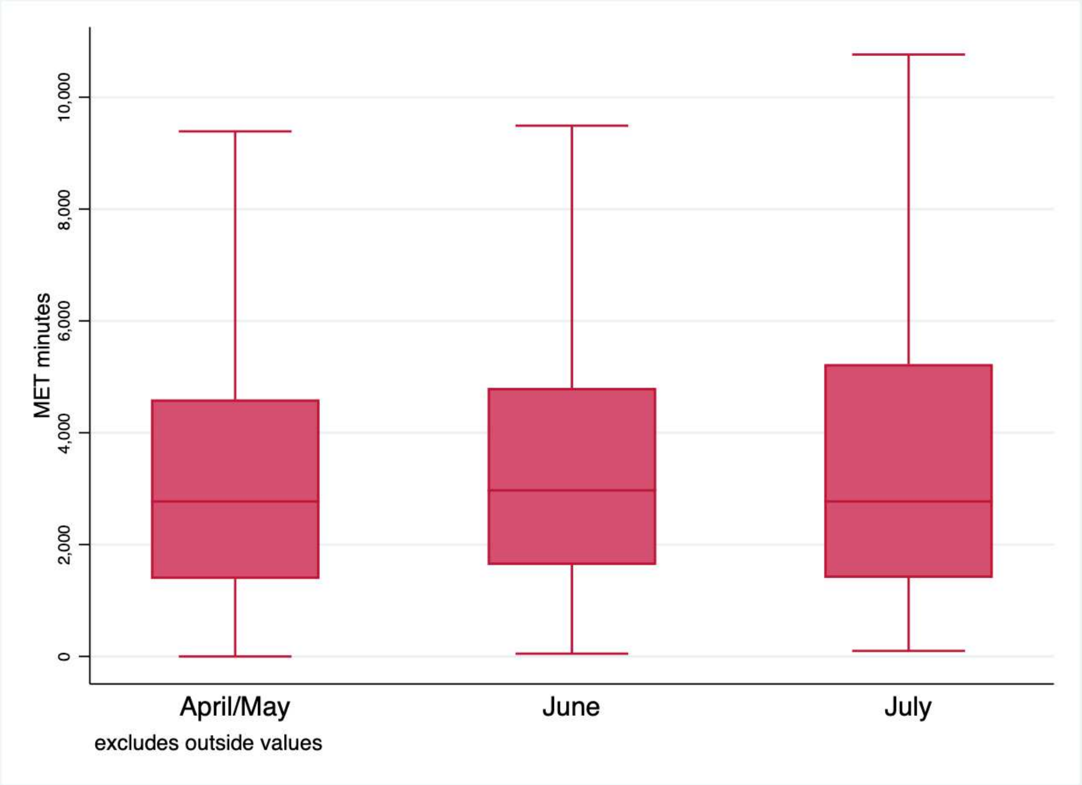

Linear regression models of MET minutes as dependent variable, against survey completion month as explanatory variable showed no significant association (p=0.1112).

Table S3: Unadjusted associations in physical activity (MET minutes per week) after introduction of lockdown measures and change from before lockdown, from linear regression models. Note: negative values for change in activity indicate reduction after lockdown

	Physical activity after lockdown (MET minutes/week)				Change in physical activity from before lockdown (MET minutes/week)			
		95% confidence interval				95% confidence interval		
Predictor	Mean	Lower	Upper	p value	Mean	Lower	Upper	p value
**Mean (whole cohort)**	3186	3120	3253	-	-333	-396	-271	-
**Age (years)**								
50-64	3341	3191	3491	<0.001	-196	-338	-55	0.184
65-74	3201	3112	3290		-362	-446	-278	
75-84	3092	2952	3232		-365	-497	-233	
85+	2326	1863	2790		-503	-940	-66	
**Sex**								
Female	3227	3138	3317	0.180	-450	-533	-366	<0.001
Male	3136	3036	3235		-189	-282	-95	
**Ethnicity**								
White	3196	3127	3265	0.425	-330	-394	-265	0.641
Mixed/multiple ethnic groups	3346	2819	3873		-392	-887	102	
Asian/Asian British	2929	2530	3327		-326	-700	48	
Black/African/Caribbean/Black British	3351	2551	4151		4	-746	754	
Other ethnic group	2754	2099	3410		-740	-1355	-125	
**Body Mass Index category**								
Underweight	3815	3137	4493	<0.001	-21	-649	607	0.055
Healthy weight	3569	3439	3700		-126	-247	-5	
Overweight	3130	2959	3300		-363	-521	-204	
Obese	2590	2309	2870		-400	-659	-140	

**Employment status**								
Employed	3093	2950	3236		-217	-351	-84	
Furloughed	3496	3122	3870	0.118	-488	-838	-139	0.101
Unemployed	3463	3031	3894		-672	-1076	-268	
Retired	3191	3111	3271		-334	-409	-259	
**Marital status**								
Divorced/single/widowed	3026	2908	3143	0.001	-540	-650	-430	<0.001
Living with a partner/married	3262	3181	3342		-236	-312	-161	
**Household**								
Not living alone	3262	3185	3340	<0.001	-240	-313	-168	<0.001
Living alone	2983	2855	3110		-582	-702	-463	
**Loneliness**								
Not ever	3284	3188	3380		-216	-306	-126	
Rarely	3087	2951	3224	0.024	-360	-488	-232	<0.001
Sometimes	3155	3010	3300		-481	-617	-345	
Often	2938	2666	3210		-762	-1018	-507	
**Shielding**								
Not shielding	3273	3196	3350	<0.001	-243	-315	-171	<0.001
Shielding	2942	2812	3072		-588	-710	-466	
**Frailty**								
Robust	3257	3183	3330		-335	-404	-265	
Pre-frail	2903	2746	3059	<0.001	-328	-475	-180	0.389
Frail	1952	1055	2849		-925	-1768	-82	
**Alcohol drinker**								
No	3044	2884	3203	0.054	-485	-634	-335	0.029
Yes	3217	3143	3290		-301	-370	-233	
**Smoker**								
No	3202	3135	3270	0.010	-322	-385	-259	0.046
Yes	2696	2318	3074		-689	-1043	-334	

**Diet**								
No change from usual - already had a healthy diet	3257	3183	3331	<0.001	-333	-402	-263	<0.001
My diet has become more healthy	3314	3119	3509		-55	-238	129	
My diet was healthy before but has got worse since lockdown	2523	2227	2818		-890	-1168	-612	
No change from usual - my diet isn't very healthy	2009	1641	2377		-479	-825	-133	
**HADS (depression score)**								
Normal (0-7)	3195	3119	3270	<0.001	-293	-365	-222	<0.001
Borderline (8-10)	2787	2495	3079		-676	-953	-399	
Abnormal (11-21)	2450	1971	2929		-1450	-1904	-997	
**HADS (anxiety score)**								
Normal (0-7)	3123	3044	3201	0.150	-312	-386	-237	0.004
Borderline (8-10)	3343	3109	3577		-348	-570	-125	
Abnormal (11-21)	3288	2971	3605		-836	-1137	-535	

*HADS – Hospital Anxiety and Depression Score

Table S4: Results of multivariable linear regression models of physical activity after lockdown, adjusted for age, sex, ethnicity, month of survey completion and baseline physical activity. Data presented as mean MET minutes/week +/- 95% confidence interval compared to the reference group, with negative values indicating lower physical activity than the reference.

Predictor	Physical activity after lockdown (MET minutes/week)	95% confidence interval		p value	Number of observations
		Lower	Upper		
**Age (years)**					
50-64 (reference)	-	-	-		
65-74	-154	-296	-12	<0.001	6155
75-84	-213	-380	-46		
85+	-640	-1034	-246		
**Sex**					
Female (reference)	-	-	-	0.053	6155
Male	108	-1	216		
**Ethnicity**					
White (reference)	-	-	-		
Mixed/multiple ethnic groups	-14	-442	415		
Asian/Asian British	-136	-463	191	0.517	6155
Black/African/Caribbean/Black British	248	-398	894		
Other ethnic group	-435	-969	100		
**Body Mass Index category**					
Underweight	153	-411	717		
Healthy weight (reference)	-	-	-	0.030	2987
Overweight	-341	-518	-165		
Obese	-578	-832	-324		
**Employment status**					
Employed (reference)	-	-	-		
Furloughed	47	-278	372	0.905	5958
Unemployed	-110	-480	259		

Retired	99	-48	246		
**Marital status**					
Divorced/single/widowed (reference)	-	-	-	<0.001	6155
Living with a partner/married	240	120	360		
**Household**					
Not living alone (reference)	-	-	-	<0.001	6155
Living alone	-277	-402	-152		
**Loneliness**					
Not ever (reference)	-	-	-		
Rarely	-161	-297	-25	<0.001	6077
Sometimes	-186	-329	-42		
Often	-452	-688	-217		
**Shielding**					
Not shielding (reference)	-	-	-	<0.001	6155
Shielding	-290	-417	-163		
**Frailty**					
Robust (reference)	-	-	-	0.005	6142
Pre-frail	-160	-301	-19		
Frail	-926	-1663	-189		
**Alcohol drinker**					
No (reference)	-	-	-	0.049	6155
Yes	145	1	289		
**Smoker**					
No (reference)				0.005	6155
Yes	-451	-762	-140		
**Diet**					
No change from usual - already had a healthy diet (reference)	-	-	-	<0.001	6155
My diet has become more healthy	156	-13	326		
My diet was healthy before but has got worse since lockdown	-662	-910	-414		

No change from usual - my diet isn't very healthy	-667	-975	-359		
**HADS (depression score)**					
Normal (0-7) (reference)	-	-	-	<0.001	5038
Borderline (8-10)	-408	-654	-163		
Abnormal (11-21)	-1007	-1401	-612		
**HADS (anxiety score)**					
Normal (0-7) (reference)	-	-	-	0.478	5038
Borderline (8-10)	94	-109	296		
Abnormal (11-21)	-220	-486	47		

HADS – Hospital Anxiety and Depression Score

Table S5: Multivariable linear regression model for physical activity after lockdown with loneliness, adjusted for age, sex, ethnicity, month of survey completion, baseline physical activity, living alone, marital status, shielding and frailty

Predictor	Physical activity after lockdown (MET minutes/week)	95% confidence interval		p value	Number of observations
		Lower	Upper		
**Loneliness**					
Not ever (reference)	-	-	-		
Rarely	-127	-265	11	0.007	6077
Sometimes	-107	-256	42		
Often	-306	-552	-60		

Adjusted: age, sex, ethnicity, month of survey completion, baseline physical activity, living alone, marital status, shielding, frailty
