# Supplementary material for: Impact of social restrictions during the COVID-19 pandemic on the physical activity levels of adults aged 50–92 years: a baseline survey of the CHARIOT COVID-19 Rapid Response prospective cohort study
Source: BMJ Open. 2021 Aug 25;11(8):e050680. doi: 10.1136/bmjopen-2021-050680 (PMC8390149; doi:10.1136/bmjopen-2021-050680)
Supplement: Supplementary data [file bmjopen-2021-050680supp002.pdf]

CHARIOT COVID-19 Rapid Response (CCRR) Study

Baseline Survey

Please answer all the questions in this survey before submitting it. Follow the prompts for those questions that are not applicable to you.

Symptoms

Q1. In the last week, have you had a cough?

<1> No

<2> Yes

Q2. In the last week, have you experienced unusual shortness of breath (difficulty breathing) compared to what's normal for you?

<1> No

<2> Yes, but it did not affect my normal activities

<3> Yes, it did affect my normal activities (eg walking short distances)

<4> Yes, even when I was sitting or lying down

Q3. In the last week, have you had a fever (feeling too hot) and did you take your temperature?

<1> I have NOT felt feverish

<2> I have felt feverish but did not check my temperature

<3> I felt feverish and my temperature was equal to, or BELOW 38 degrees Celcius

<4> I felt feverish and my temperature measured ABOVE 38 degrees Celcius

Q4. In the last week, have you experienced any of these other symptoms? Please do NOT include symptoms you experience on a regular basis due to a health condition you already know about. Please tick all that apply:

<1> Loss of sense of smell

<2> Loss of sense of taste

<3> Decrease in appetite (skipping meals)

<4> Diarrhoea

<5> Nauseas and/or Vomiting

<6> Abdominal pain/tummy ache

<7> Chills (feeling too cold)

<8> Difficulty sleeping

<9> Felt more tired than normal

<10> Severe Fatigue

<11> Sneezing

<12> Chest pain / tightness

<13> Tightness in chest

<14> Sore throat

<15> Hoarse voice

<16> Runny nose

<17> Blocked nose

<18> Sore eyes

<19> Itchy eyes

<20> Headache

<21> Joint pain / aches

<22> Dizziness

<23> Muscle pain/aches

<99> None of these

If you answered, 'None of these', please skip Q5 and go to Q6.

Q5. Thinking about the 14 days before your symptoms started, had you been in physical contact (within 2 metres / 6 feet) with someone who has a confirmed diagnosis of coronavirus (Covid-19), or someone with the following symptoms: dry cough, fever, loss of sense of smell, loss of sense of taste, shortness of breath or difficulty breathing.

<1> Yes, and it was an individual within my household

<2> Yes, and it was an individual from outside my household

<3> No, not that I am aware of

QX Since COVID-19 emerged in January, but before the official lockdown started on March 23rd 2020, which, if any of the following, have you experienced? Please do NOT include symptoms you experience on a regular basis due to a health condition you already know about. Please tick all that apply.

<1> New, continuous cough (coughing a lot for more than an hour, or have had 3 coughing episodes in 24 hours)

<2> High temperature (hot to touch on chest or back)

<3> Loss of sense of smell

<4> Loss of sense of taste

<5> Loss of appetite (skipping meals)

<6> Diarrhoea

<7> Vomiting

<8> Fatigue

<9> Sneezing

<10> Chest pain / tightness

<11> Sore throat

<12> Runny nose

<13> Itchy eyes

<14> Headache

<15> Joint pain / aches

<16> Muscle or joint pain

<99> None of these

If you answered, 'None of these', go to Q6.

QXa Approximately when did you start experiencing these symptoms?

[DD/MM/YYYY]

QXb Approximately how long did these symptoms last?

[Days:]

QXX Thinking about the 14 days before your symptoms started, had you been in physical contact (within 2 metres / 6 feet) with someone who has a confirmed diagnosis of coronavirus (Covid-19), or someone with the following symptoms: dry cough, fever, loss of sense of smell, loss of sense of taste, shortness of breath or difficulty breathing.

<1> Yes, and it was an individual within my household

<2> Yes, and it was an individual from outside my household

<3> No, not that I am aware of

Q6 Now, thinking about the period prior to last week, but after the official lockdown started on 23rd March 2020 , which, if any of the following, have you experienced? Please do NOT include symptoms you experience on a regular basis due to a health condition you already know about. Please tick all that apply.

<1> Fever (feeling too hot)

<2> New persistent cough

<3> Shortness of breath affecting normal activities

<4> Loss of sense of smell

<5> Loss of sense of taste

<6> Decrease in appetite (skipping meals)

<7> Diarrhoea

<8> Nauseas and/or vomiting

<9> Abdominal pain/tummy ache

<10> Chills (feeling too cold)

<11> Difficulty sleeping

<12> Felt more tired than normal

<13> Severe fatigue

<14> Sneezing

<15> Chest pain

<16> Tightness in chest

<17> Sore throat

<18> Hoarse throat

<19> Runny nose

<20> Blocked nose

<21> Sore eyes

<22> Itchy eyes

<23> Headache

<24> Dizziness

<25> Joint pain / aches

<26> Muscle pain/aches

If you answered, 'None of these', go to Q8.

Q6a Approximately when did you start experiencing these symptoms?

[DD/MM/YYYY]

Q6b Approximately how long did these symptoms last?

[Days:]

Q7 Thinking about the 14 days before your symptoms started, had you been in physical contact (within 2 metres / 6 feet) with someone who has a confirmed diagnosis of coronavirus (Covid-19), or someone with the following symptoms: dry cough, fever, loss of sense of smell, loss of sense of taste, shortness of breath or difficulty breathing.

<1> Yes, and it was an individual within my household

<2> Yes, and it was an individual from outside my household

<3> No, not that I am aware of

Q8 Have you or anyone in your house been tested for coronavirus? Please tick all that apply

<1> No testing

<2> I have not been tested -- BUT I think I have already had coronavirus and recovered

<3> I was tested - positive result

<4> I was tested - awaiting result

<5> I was tested - negative result

<6> Household member tested - positive result

<7> Household member tested - awaiting result

<8> Household member tested - negative result

Q9 In the last week, has anyone in your household had a new cough or fever?

Not applicable

<1> No

<2> Yes

Q10 Have you had any healthcare contact since the lockdown started? Please tick all that apply

<1> No

<2> Yes - remote appointment with my GP (phone/video)

<3> Yes - I attended my GP practice for an appointment

<4> Yes - remote appointment with hospital (phone/video)

<5> Yes - I attended hospital for an appointment

<6> Yes - attended Accident and Emergency

<7> Yes -- I was admitted to hospital (not because of coronavirus)

<8> Yes -- I was admitted to hospital with symptoms of coronavirus

<9> Yes – One or more remote calls to 111- home visit by ambulance

Q11. In the last week, have you been taking any medication for new symptoms?

<1> No

<2> Yes

<3> If yes, what medication?

Underlying conditions

For the following question, please remember that your answers are always treated confidentially and are never analysed individually. We have provided you with a "Prefer not to say" option if you would rather not share your experiences.

Q12 Which, if any, of the following chronic health conditions have you been diagnosed with? (Please select all that apply. If you do not currently have a chronic health condition, please select the 'None of these' option)

<1> Arthritis

<2> Asthma

<3> My doctor has told me I have severe asthma

<4> I am having cancer treatment

<5> Blood or bone marrow cancer, such as leukaemia

<6> Cystic fibrosis

<7> Chronic obstructive pulmonary disease (COPD)

<8> Diabetes

<9> Epilepsy

<10> Heart disease

<11> High blood pressure

<12> High cholesterol

<13> HIV/ AIDS

<14> Mental health condition

<15> Multiple Sclerosis

<16> I have had an organ transplant

<17> I have a condition that makes me much more likely to get infections

<18> I am taking medicine that weakens my immune system

<19> Dementia, Parkinson's or other neurological disease

<98> Prefer not to say

<99> None of these

Contacts

[Q13 What is your date of birth:

Date …..]

Q14 What is your sex:

<1> Female

<2> Male

<3> Prefer not to say

Q15 What ethnic group best describes you? Please select one option only.

<1>

English / Welsh / Scottish / Northern Irish / British

<11>

Bangladeshi

<2>

Irish

<12>

Chinese

<3>

Gypsy or Irish Traveller

<13>

Any other Asian background

<4>

Any other White background

<14>

African

<5>

White and Black Caribbean

<15>

Caribbean

<6>

White and Black African

<16>

Any other Black / African / Caribbean background

<7>

White and Asian

<17>

Arab

<8>

Any other Mixed / Multiple ethnic background

<18 fixed>

Any other ethnic group

<9>

Indian

<19 fixed>

Prefer not to say

<10>

Pakistani

Q16 Who else is CURRENTLY living in your household? Please tick all that apply

<1> I live by myself

<2> I live with my partner

<3> I live with my child/children aged under 18

<4> I live with my child/children aged over 18

<5> I live with family members other than partner / children

<6> I live with housemates

16a Where are you living during lockdown?

<1> My usual home

<2> not my usual home – keeping away from household members who are at high risk coronavirus

<3> Not my usual home – other reason

Q17 How many people, including yourself, are there in your household? Please include both adults and children. If you live alone, enter 1

Number

Q17a

For each household member ask age (in years) and sex

The following questions will ask you to report on how many people you have come into contact with both inside and outside of your household.

A contact is defined as either:

• Direct skin-to-skin physical contact (e.g. kiss/embrace/handshake)

• Face-to-face conversation with another person which lasts over 3 mins, within 2m distance

• Being within 2m distance from another individual for over 5 mins

Note: if you contacted the same person in different times through the day, they should be counted once.

Q18 How many different people did you have contact with, both inside your household and while outside (after having left your household) in the past 7 days?

Enter 0 if you had no contacts in the last 7 days

1. (enter number)

2. Don't know

Q19a Among the contacts you had, just from yesterday, both inside your household and while outside (after having left your household), how many belonged to the following age groups?

No contacts yesterday

0 to <10 years old (enter number)

10 to <20 years old (enter number)

20 to <30 years old (enter number)

30 to <40 years old (enter number)

40 to <50 years old (enter number)

50 to <60 years old (enter number)

60 to <70 years old (enter number)

70 to <80 years old (enter number)

80 to <90 years old (enter number)

90+ years old (enter number)

12. Don't know

IF Q18 is NOT=0

Q19b How many different people did you come in contact with in the past 7 days outside of your household?

Enter 0 if you had no contacts in the last 7 days outside of your household

1. (enter number)

2. Don't know

IF Q19b is NOT=0

Q19c Among the contacts you had, just from yesterday, outside your household, how many belonged to the following age groups?

No contacts yesterday

0 to <10 years old (enter number)

10 to <20 years old (enter number)

20 to <30 years old (enter number)

30 to <40 years old (enter number)

40 to <50 years old (enter number)

50 to <60 years old (enter number)

60 to <70 years old (enter number)

70 to <80 years old (enter number)

80 to <90 years old (enter number)

90+ years old (enter number)

Don't know

IF Q19b is NOT=0

Q19c Among the contacts that you have had in the past 7 days outside your household, how many contacts occurred at work?

Enter 0 if you had no contacts in the last 7 days outside of your household that occurred at work

1. (enter number)

2. Don't know

For the following questions please answer according to the following terms;

Self-isolation – refers to those who are symptomatic and self-isolating for 7 days from when symptoms started

Shielding – those in specific vulnerable groups staying at home for 12 weeks. These groups would include those with underlying chronic health conditions: cancers, respiratory disease, on immunosuppressants, those at increased risk of infection or pregnant women with heart disease and/or those advised by the NHS of their extremely vulnerable status'.

Household quarantine – 14-day quarantine period for all members of a household from the first day of symptom onset in first case in that household

Social distancing and isolation

Q20 Are you currently in self-isolation?

<1> Yes

<2> No

If yes, for how long:…days

Q21 Are you currently shielding as per government guidelines for vulnerable groups?

<1> Yes

<2> No

Q22 Have you moved residence recently due to the pandemic? Y/N

Q23. Are you single, married, living with a partner, divorced, widowed?

Q24. Are you

<1> Continuing to work in your usual job; at home

<2> Continuing to work in your usual job and leave home for your job <3> volunteering in response to the COVID pandemic

<4> a key worker

<5> unemployed

<6> retired

<7> furloughed (put on leave, still getting paid)

<8> had to close your business due to COVID-19

<9> lost my job due to the lockdown

<10> a student

<99> None of the above

Q25. How often are you now contacting friends/family members remotely (Skype/Zoom/Mobile/landline phone etc)?

Several times per day, once a day, 2-3 x per week, 4-6 x per week, once a week, less than once a week?

Q26 Overall, how are your relationships with other members of your household?

Not applicable

1 = worst

2

3

4

5

6

7

8

9

10 = best

Q27 If you are leaving your home, what activity is this for? Please tick all that apply

<1> I am not leaving my home

<2> Commute to work

<3> Essential shopping

<4> Exercise

<5> Other

Q28 Have you or anyone in your household received a letter or message informing you that you are in the population at 'high risk' from coronavirus? Please tick all that apply.

<1> No - Neither myself or anyone in my household is at 'high risk'

<2> No - but I think I should have

<3> No - but someone in my household is at 'high risk'

<4> Yes - letter about me

<5> Yes - letter about someone in my household

Health behaviours: dietary, alcohol and smoking

Q29 Do you drink alcohol?

<1> Yes (If yes trigger sub-questions)

Drinking less since lockdown

Drinking the same amount since lockdown

Drinking more since lockdown

How many units do you consume per week:…units

(half pint/ 300ml = approx. 1 unit, 175ml glass wine= approx. 2 units)

<2> No (If no, trigger sub-questions)

I never drink alcohol

I had already stopped drinking alcohol before lockdown

I stopped drinking alcohol when lockdown started

Q30 Do you smoke?

<1> Yes (if yes, trigger sub-questions)

Smoking less since lockdown

Smoking the same amount since lockdown

Smoking more since lockdown

If yes, how many cigarettes or roll-ups do you smoke per day:…

<2> No (if now, trigger sub-questions)

I never smoked

I had already stopped smoking before lockdown

I stopped smoking since the lockdown

Q30a) Has there been a change in your vaping (e-cigarettes) status since the coronavirus lockdown?

<1> I never vaped

<2> I had already stopped vaping before

<3> I stopped vaping since the lockdown

<4> Vaping less

<5> Vaping the same amount

<6> Vaping more

Q31 Since the lockdown, are you managing to keep a healthy diet, for example, eating fresh fruits and vegetables?

<1> No change from usual - already had a healthy diet

<2> No change from usual - my diet isn't very healthy

<3> My diet has become more healthy

<4> My diet was healthy before but has got worse since lockdown

Q32 On average, how many portions (or servings) of fruit and vegetables do you eat per day?.......

- One portion is typically 80g, 3 heaped tablespoons of cooked veg or 1 cereal bowl of mixed salad
- Three heaped tablespoons of beans and other pulse vegetables, such as kidney beans, lentils and chickpeas, count as 1 portion.
- The following starchy vegetables should not be included – potatoes, yams, cassava and plantain

Q32a Have you ever skipped meals due to difficulties accessing food as a result of COVID-19?

Yes /No

If yes:

How many meals per week, on average have you missed?

<1> 1-3 meals per week

<2> 4-6 meals per week

<4> 7-9 meals per week

<5> 10 or more meals per week

Biometric data: height and weight

Q33 Please enter your weight: Kg

Q34 Please enter your height:…cm

Q35 Do you have a recent (from the past week) blood pressure?_____mm/Hg

Current Physical activity: International Physical Activity Questionnaire

We are interested in finding out about the kinds of physical activities that people do as part of their everyday lives. The questions will ask you about the time you spent being physically active in the last 7 days. Please answer each question even if you do not consider yourself to be an active person. Please think about the activities you do at work, as part of your house and garden work, to get from place to place, and in your spare time for recreation, exercise or sport.

Think about all the vigorous activities that you did in the last 7 days. Vigorous physical activities refer to activities that take hard physical effort and make you breathe much harder than normal. Think only about those physical activities that you did for at least 10 minutes at a time.

Q36: During the last 7 days, on how many days did you do vigorous physical activities like heavy lifting, digging, aerobics, or fast bicycling?

_____ *days per week*

If no vigorous physical activities, skip to question 38

Q37: How much time did you usually spend doing vigorous physical activities on one of those days? If you only exercised in hours or minutes, please input a '0' in the non-applicable field.

_____ *hours per day*

_____ minutes per day

Think about all the moderate activities that you did in the last 7 days. Moderate activities refer to activities that take moderate physical effort and make you breathe somewhat harder than normal. Think only about those physical activities that you did for at least 10 minutes at a time.

Q38: During the last 7 days, on how many days did you do moderate physical activities like carrying light loads or bicycling at a regular pace? Do not include walking.

_____ days per week

If no moderate physical activities, skip to question 40

Q39: How much time did you usually spend doing moderate physical activities on one of those days? If you only exercised in hours or minutes, please input a '0' in the non-applicable field.

_____ hours per day

_____ minutes per day

Think about the time you spent walking in the last 7 days. This includes at work and at home, walking to travel from place to place, and any other walking that you have done solely for recreation, sport, exercise, or leisure.

Q40: During the last 7 days, on how many days did you walk for at least 10 minutes at a time?

_____ days per week

No walking, skip to question 42

Q41: How much time did you usually spend walking on one of those days? If you only exercised in hours or minutes, please input a '0' in the non-applicable field.

_____ hours per day

_____ minutes per day

The last question is about the time you spent sitting on weekdays during the last 7 days. Include time spent at work, at home, while doing course work and during leisure time. This may include time spent sitting at a desk, reading, or sitting or lying down to watch television.

Q42: During the last 7 days, how much time did you spend sitting on a week day?

If you only exercised in hours or minutes, please input a '0' in the non-applicable field.

_____ *hours per day*

_____ *minutes per day*

Previous Physical activity: International Physical Activity Questionnaire

These questions will ask you about the time you spent being physically active in the 7 days prior to implementation of social distancing measures (please use first week of March 2020). Please answer each question even if you do not consider yourself to be an active person. Please think about the activities you do at work, as part of your house and garden work, to get from place to place, and in your spare time for recreation, exercise or sport.

Think about all the vigorous activities that you did in the 7 days prior to social distancing measures. Vigorous physical activities refer to activities that take hard physical effort and make you breathe much harder than normal. Think only about those physical activities that you did for at least 10 minutes at a time.

Q43: During the 7 days prior to social distancing measures (please use first week of March 2020), on how many days did you do vigorous physical activities like heavy lifting, digging, aerobics, or fast bicycling?

_____ *days per week*

If no vigorous physical activities, skip to question 45

Q44: How much time did you usually spend doing vigorous physical activities on one of those days? If you only exercised in hours or minutes, please input a '0' in the non-applicable field.

_____ *hours per day*

_____ *minutes per day*

Think about all the moderate activities that you did in the 7 days prior to social distancing measures. Moderate activities refer to activities that take moderate physical effort and make you breathe somewhat harder than normal. Think only about those physical activities that you did for at least 10 minutes at a time.

Q45: During the 7 days prior to social distancing measures (please use first week of March 2020), on how many days did you do moderate physical activities like carrying light loads or bicycling at a regular pace? Do not include walking.

_____ *days per week*

If no moderate physical activities, skip to question 47

Q46: How much time did you usually spend doing moderate physical activities on one of those days? If you only exercised in hours or minutes, please input a '0' in the non-applicable field.

_____ *hours per day*

_____ *minutes per day*

Think about the time you spent walking in the 7 days prior to social distancing measures. This includes at work and at home, walking to travel from place to place, and any other walking that you have done solely for recreation, sport, exercise, or leisure.

Q47: During the 7 days prior to social distancing measures (please use first week of March 2020), on how many days did you walk for at least 10 minutes at a time?

_____ *days per week*

No walking, skip to question 49

Q48: How much time did you usually spend walking on one of those days? If you only exercised in hours or minutes, please input a '0' in the non-applicable field.

_____ *hours per day*

_____ *minutes per day*

The last question is about the time you spent sitting on weekdays during 7 days prior to social distancing measures. Include time spent at work, at home, while doing course work and during leisure time. This may include time spent sitting at a desk, reading, or sitting or lying down to watch television.

Q49: During the 7 days prior to social distancing measures (please use first week of March 2020), how much time did you spend sitting on a week day? If you only exercised in hours or minutes, please input a '0' in the non-applicable field.

_____ *hours per day*

_____ *minutes per day*

Frailty Questionnaire:

Q50: Are you fatigued?

<1> Yes

<2> No

Q51: Can you walk up one flight of stairs?

<1> Yes

<2> No

Q52: Can you walk around the block?

<1> Yes

<2> No

Q53: Do you have more than 5 illnesses?

<1> Yes

<2> No

Q54: Have you lost more than 5% of your weight in the past 6 months?

<1> Yes

<2> No

Qx Have you had a fall during the COVID lockdown period?

Yes/No

If yes:

What actions were taken (select multiple where applicable):

a) No follow-up required, I did not hurt myself

b) Pain medication

c) A hospital and/or GP appointment

d) A follow-up X-ray

e) Sling/plaster cast for a fracture

f) Surgery

Mood

a) Tick the box beside the reply that is closest to how you have been feeling in the past week.

Don't take too long over you replies: your immediate response is best.

Tick here

1.

Tick here

8.

I feel tense or 'wound up':

I feel as if I am slowed down:

Most of the time

Nearly all the time

A lot of the time

Very often

From time to time, occasionally

Sometimes

Not at all

Not at all

2.

9.

I still enjoy the things I used to

enjoy:

I get a sort of frightened feeling like 'butterflies' in the stomach:

Definitely as much

Not at all

Not quite so much

Occasionally

Only a little

Quite Often

Hardly at all

Very Often

3.

10.

I get a sort of frightened feeling as if something awful is about to

happen:

I have lost interest in my appearance:

Very definitely and quite badly

Definitely

Yes, but not too badly

I don't take as much care as I should

A little, but it doesn't worry me

I may not take quite as much care

Not at all

I take just as much care as ever

4.

11.

I can laugh and see the funny side of things:

I feel restless as I have to be on the move:

As much as I always could

Very much indeed

Not quite so much now

Quite a lot

Definitely not so much now

Not very much

Not at all

Not at all

5.

12.

Worrying thoughts go through my mind:

I look forward with enjoyment to things:

A great deal of the time

As much as I ever did

A lot of the time

Rather less than I used to

From time to time, but not too often

Definitely less than I used to

Only occasionally

Hardly at all

6.

13.

I feel cheerful:

I get sudden feelings of panic:

Not at all

Very often indeed

Not often

Quite often

Sometimes

Not very often

Most of the time

Not at all

7.

14.

I can sit at ease and feel relaxed:

I can enjoy a good book or radio or TV program:

Definitely

Often

Usually

Sometimes

Not Often

Not often

Not at all

Very seldom

Qx . I experience a general sense of emptiness

- *Not ever*
- *Rarely*
- *Sometimes*
- *Often*

Qy. There are plenty of people I can rely on when I have problems

- *Not ever*
- *Rarely*
- *Sometimes*
- *Often*

Qz. I miss having people around me

- *Not ever*
- *Rarely*
- *Sometimes*
- *Often*

Please check you have answered all the questions above.

b) For each of the 17 mood questions above, please also indicate if you are feeling or experiencing this 1, less than; 2, the same as; or 3, more than before social isolation was implemented.

<1> [1 or 2 or 3]

<2> [1 or 2 or 3]

<3> [1 or 2 or 3]

<4> [1 or 2 or 3]

<5> [1 or 2 or 3]

<6> [1 or 2 or 3]

<7> [1 or 2 or 3]

<8> [1 or 2 or 3]

<9> [1 or 2 or 3]

<10> [1 or 2 or 3]

<11> [1 or 2 or 3]

<12> [1 or 2 or 3]

<13> [1 or 2 or 3]

<14> [1 or 2 or 3]

<15> [1 or 2 or 3]

<16> [1 or 2 or 3]

<17> [1 or 2 or 3]

Q. People may have worries about Covid-19. Have you been worried about any of the following and, if so, how much?

Not at all

Little

Some

Rather much

Very much

Getting Covid-19 infection and/or infecting someone else

That a person close to me could get infected with Covid-19

Being discriminated against or avoided because of Covid-19

Impact of the Covid-19 epidemic on my own

economy and/or loss of my employment

Economic impact of the Covid-19 epidemic on the global economy

The government's and/or health system's lack of ability to handle the Covid-19 pandemic situation, including the shortage of food and other groceries

Imperial College Sleep Quality (ICSQ) Questionnaire

Instructions:

The following questions relate to your usual sleep habits for a period of one month before and during a period of reduced social contact. Your answers should indicate the most accurate reply for the majority of days and nights during these periods.

Please answer all questions.

1. During the period before reduced social contact, what time did you usually go to bed at night: bed-time was …………………………………………………

1b) During the period of reduced social contact, what time have you usually gone to bed at night: bed-time is - ……………………………………………………......

2. During the period before reduced social contact, how long (in minutes) did it usually take you to fall asleep each night: number of minutes - …………………

2b) During the period of reduced social contact, how long (in minutes) has it usually taken you to fall asleep each night: number of minutes - ……………………………

3. During the period before reduced social contact, what time did you usually get up in the morning: getting-up time was - ………………………………………

3b) During the period of reduced social contact, what time do you usually get up in the morning: getting-up time is - ……………………………………………………….

4. During the period before reduced social contact, how many hours of actual sleep did you get at night? (This may be different from the number of hours you

spent in bed): hours of sleep per night - …………………………………………

4b) During the period of reduced social contact, how many hours of actual sleep do you get at night? (This may be different from the number of hours you spend in bed): hours of sleep per night - ………………………………………………...

5. During the period before reduced social contact, how often did you have trouble sleeping because you could not get to sleep within 30 minutes:

o Not ever

o Less than once a week

o Once or twice a week

o Three or more times a week

5b) During the period of reduced social contact, how often have you had trouble sleeping because you could not get to sleep within 30 minutes:

o Not ever

o Less than once a week

o Once or twice a week

o Three or more times a week

6. During the period before reduced social contact, did you experience poor sleep (restless and unable to sleep):

o Not ever

o Less than once a week

o Once or twice a week

o Three or more times a week

6b) During the period of reduced social contact, have you experienced poor sleep (restless and unable to sleep):

o Not ever

o Less than once a week

o Once or twice a week

o Three or more times a week

7a) During the period before reduced social contact, did you experience loneliness (felt isolated, with no companions):

o Not ever

o Rarely

o Sometimes

o Often

7b) During the period of reduced social contact, have you experienced loneliness (felt isolated, with no companions):

o Not ever

o Rarely

o Sometimes

o Often

7c) During the period of reduced social contact, have you experienced loneliness: 1,

less than; 2, the same as; or 3, more than before social isolation was implemented

Select: [1 or 2 or 3]

Functional Activities Questionnaire

For each of the tasks below please rate your ability to carry out the task/activity independently on the following scale:

1. I had no difficulty

2. I had some difficulty, but I completed the task/activity myself.

3. I need some assistance to complete the task/activity:

a) I did not need assistance prior to COVID-19 lockdown but need assistance now to maintain social isolation/distancing

b) I could do the task/activity before the COVID-19 lockdown, but now would need assistance even if it were not to maintain social distancing

c) I required assistance since before the COVID-19 lockdown

4. I needed others to do this for me,

a) I could do the task/activity myself or with assistance prior to COVID-19 lockdown but need others to do it for me to maintain social isolation/distancing

b) I could do the task/activity myself or with assistance before the COVID-19 lockdown, but now would need others to do it for me even if it were not to maintain social distancing

c) I required others to do it for me since before the COVID-19 lockdown

5. I am unsure if I require assistance (e.g., never did the task/activity or have not done the task/activity over the past week)

Activities:

1. Writing cheques, paying bills, balancing cheque book, using an ATM cash machine

Response: …………………

2. Assembling tax records, business affairs, or papers

Response: …………………

3. Shopping alone for household necessities, medicines or groceries

Response: …………………

4. Playing a game of skill, working on a hobby

Response: …………………

5. Heating water, making a cup of coffee, turning off stove after use

Response: …………………

6. Preparing a balanced meal

Response: …………………

7. Keeping track of current events

Response: …………………

8. Paying attention to, understanding, discussing TV, video, book, magazine

Response: …………………

9. Remembering appointments, family occasions, public holidays, to take medications

Response: …………………

10. Travelling out of my neighbourhood by taxi, car, bus or train and making travel arrangements.

Response: …………………

THANK YOU FOR COMPLETING THIS QUESTIONNAIRE. YOUR RESPONSES HAVE BEEN SAVED AND SENT TO THE STUDY TEAM.

NHS health advice and information regarding the novel coronavirus can be found here: https://www.nhs.uk/conditions/coronavirus-covid-19/

For Advice on Mental health we suggest using these links:

1. The NHS Every Mind Matters website has information on how to look after your mental wellbeing while in isolation: https://www.nhs.uk/oneyou/every-mind-matters/

2. The charity Mental Health UK have advice on managing mental health during the coronavirus outbreak: https://mentalhealth-uk.org/help-and-information/covid-19-and-your-mental-health/

3. The NHS recommends a range of mobile apps to help with mental wellbeing, many of which are free to download: https://www.nhs.uk/apps-library/category/mental-health/

4. If you need someone to talk to about your mental health, the charity Samaritans have a helpline available 24 hours a day, 7 days a week:

a. Call: 116 123

b. or visit: https://www.samaritans.org/how-we-can-help/contact-samaritan/

For Advice on Physical activity we suggest using these links:

1. The NHS Live Well website has a range of free advice and programmes from light activity to more strenuous exercises for those aged under 65: https://www.nhs.uk/live-well/exercise/

2. The NHS Live Well website has a range of free advice and programmes from light activity to more strenuous exercises for those aged 65 or older: https://www.nhs.uk/live-well/exercise/physical-activity-guidelines-older-adults/

3. Tips, advice and guidance from Sport England on how to keep or get active in and around your home: https://www.sportengland.org/stayinworkout

4. Stay Active at Home: a simple set of exercises designed for older people to stay active at home: https://www.csp.org.uk/public-patient/keeping-active-and-healthy/staying-healthy-you-age/staying-strong-you-age/strength

For Advice on Sleep we suggest using these links:

1. The NHS ten top tips to improve sleep: https://www.nhs.uk/live-well/sleep-and-tiredness/10-tips-to-beat-insomnia/

2. The NHS recommends a range of mobile apps to help with sleep: https://www.nhs.uk/apps-library/category/sleep/

Supplementary Table 1: CCRR survey
