# Supplementary material for: Impact of social restrictions during the COVID-19 pandemic on the physical activity levels of adults aged 50–92 years: a baseline survey of the CHARIOT COVID-19 Rapid Response prospective cohort study
Source: BMJ Open. 2021 Aug 25;11(8):e050680. doi: 10.1136/bmjopen-2021-050680 (PMC8390149; doi:10.1136/bmjopen-2021-050680)
Supplement: Author's manuscript [file bmjopen-2021-050680.draft_revisions.pdf]

BMJ Open is committed to open peer review. As part of this commitment we make the peer review history of every article we publish publicly available.

When an article is published we post the peer reviewers' comments and the authors' responses online. We also post the versions of the paper that were used during peer review. These are the versions that the peer review comments apply to.

The versions of the paper that follow are the versions that were submitted during the peer review process. They are not the versions of record or the final published versions. They should not be cited or distributed as the published version of this manuscript.

BMJ Open is an open access journal and the full, final, typeset and author-corrected version of record of the manuscript is available on our site with no access controls, subscription charges or pay-per-view fees (http://bmjopen.bmj.com).

If you have any questions on BMJ Open's open peer review process please email info.bmjopen@bmj.com

BMJ Open

The impact of social restrictions during the COVID-19 pandemic on the physical activity levels of older adults: a baseline analysis of the CHARIOT COVID-19 Rapid Response prospective cohort study

Journal:	*BMJ Open*
Manuscript ID	bmjopen-2021-050680
Article Type:	Original research
Date Submitted by the Author:	25-Feb-2021
Complete List of Authors:	Salman, David; Imperial College London Department of Primary Care and Public Health; Imperial College London, MSk lab, Department of Surgery and Cancer, Faculty of Medicine<br>Beaney, Thomas; Imperial College London, Department of Primary Care and Public Health<br>Robb, Catherine; Imperial College London, Ageing Epidemiology Research Unit (AGE), School of Public Health, Faculty of Medicine<br>de-Jaegar Loots, Celeste; Imperial College London, Ageing Epidemiology Research Unit (AGE), School of Public Health, Faculty of Medicine<br>Giannakopoulou, Parthenia; Imperial College London, Ageing Epidemiology Research Unit (AGE), School of Public Health, Faculty of Medicine<br>Udeh-Momoh, Chinedu ; Imperial College London, Ageing Epidemiology Research Unit (AGE), School of Public Health, Faculty of Medicine<br>Ahmadi-Abhari, Sara ; Imperial College London, Ageing Epidemiology Research Unit (AGE), School of Public Health, Faculty of Medicine<br>Majeed, Azeem; Imperial College London, Department of Primary Care and Public Health; Imperial College Healthcare NHS Trust, Public Health Directorate<br>Middleton, Lefkos; Imperial College London, Ageing Epidemiology Research Unit (AGE), School of Public Health, Faculty of Medicine; Imperial College Healthcare NHS Trust, Public Health Directorate<br>McGregor, Alison; Imperial College London, MSk lab, Department of Surgery and Cancer, Faculty of Medicine
Keywords:	COVID-19, PREVENTIVE MEDICINE, PUBLIC HEALTH, SPORTS MEDICINE, GERIATRIC MEDICINE

SCHOLARONE™
Manuscripts

I, the Submitting Author has the right to grant and does grant on behalf of all authors of the Work (as defined in the below author licence), an exclusive licence and/or a non-exclusive licence for contributions from authors who are: i) UK Crown employees; ii) where BMJ has agreed a CC-BY licence shall apply, and/or iii) in accordance with the terms applicable for US Federal Government officers or employees acting as part of their official duties; on a worldwide, perpetual, irrevocable, royalty-free basis to BMJ Publishing Group Ltd ("BMJ") its licensees and where the relevant Journal is co-owned by BMJ to the co-owners of the Journal, to publish the Work in this journal and any other BMJ products and to exploit all rights, as set out in our licence.

The Submitting Author accepts and understands that any supply made under these terms is made by BMJ to the Submitting Author unless you are acting as an employee on behalf of your employer or a postgraduate student of an affiliated institution which is paying any applicable article publishing charge ("APC") for Open Access articles. Where the Submitting Author wishes to make the Work available on an Open Access basis (and intends to pay the relevant APC), the terms of reuse of such Open Access shall be governed by a Creative Commons licence – details of these licences and which Creative Commons licence will apply to this Work are set out in our licence referred to above.

Other than as permitted in any relevant BMJ Author's Self Archiving Policies, I confirm this Work has not been accepted for publication elsewhere, is not being considered for publication elsewhere and does not duplicate material already published. I confirm all authors consent to publication of this Work and authorise the granting of this licence.

For peer review only

The impact of social restrictions during the COVID-19 pandemic on the physical activity levels of older adults: a baseline analysis of the CHARIOT COVID-19 Rapid Response prospective cohort study

David Salman, academic clinical fellow in primary care*[1,2@], **Thomas Beaney,** academic clinical fellow in primary care*[2@], **Catherine E. Robb,** postgraduate research associate*[3], Celeste A. de Jager Loots, research fellow[3], Parthenia Giannakopoulou, data management coordinator[3], Chi Udeh-Momoh, research programme manager[3], Sara Ahmadi-Abhari, lecturer in epidemiology of ageing[3], Azeem Majeed, chair: primary care and public health[2,4], Lefkos T. Middleton, chair: neurology, neuroepidemiology and ageing[3,4], Alison. H. McGregor, professor of musculoskeletal biodynamics[1]

*contributed equally as joint lead authors

Author affiliations:

[1]MSk lab, Faculty of Medicine, Imperial College London, UK, W12 0BZ

[2]Department of Primary Care and Public Health, Imperial College London, UK, W6 8RP

[3]Ageing Epidemiology Research Unit (AGE), School of Public Health, Faculty of Medicine, Imperial College London, UK

[4] Public Health Directorate, Imperial College NHS Healthcare Trust, London, UK

[@]**Correspondence to:**

David Salman

ORCID ID: 0000-0002-1481-8829

MSk lab, 2nd Floor, Sir Michael Uren Hub, 86 Wood Lane, London, W12 0BZ

Email: d.salman11@imperial.ac.uk

Phone: +44 (0) 20 7594 2703

Thomas Beaney

ORCID ID: 0000-0001-9709-7264

Department of Primary Care and Public Health

Charing Cross Campus, The Reynolds Building, St Dunstan's Road, London, W6 8RP

Email: thomas.beaney@imperial.ac.uk

Phone: +44 (0) 207 5943 368

Word count: 3683

Figures:3

Tables:2

For peer review only

Contributorship and the guarantor

DS, TB and CR conceived the paper, developed the survey materials, carried out the analysis, wrote the paper equally as joint lead authors and are the guarantors. All authors developed the survey, carried out analysis and contributed to the development and editing of the paper.

Transparency declaration

The lead authors confirm that the submitted manuscript is an honest, accurate and transparent account of the study being reported. No important aspects of the study have been omitted.

Ethics approval

This research was approved by the Imperial College Research and Ethics Committee (ICREC) and Joint Research Compliance Office (22/04/2020; 20IC5942). All participants were required to provide informed consent before taking part in the study. Data collected as a part of this study are anonymized and kept strictly confidential in accordance with the UK General Data Protection Regulations (2016).

Data sharing

This is an ongoing study, but anonymised data can be provided upon request for the purposes of further data analysis, and can be requested from the Data Management Co-ordinator, Parthenia Giannakopoulou: parthenia.giannakopoulou13@imperial.ac.uk

Dissemination declaration

Participants in the CHARIOT cohort are informed by regular newsletter of all publications pertaining to the cohort.

Acknowledgements

Work towards this article was in part supported by the National Institute for Health Research (NIHR) Applied Research Collaboration Northwest London and Imperial Biomedical Research Centre (BRC). DS and TB are supported by NIHR academic clinical fellowships. The views expressed in this publication are those of the authors and not necessarily those of the National Institute for Health Research or the Department of Health and Social Care. Imperial College London is the sponsor for the CCRR study, and has no influence on the direction or content of the work. There was no external financial funding for the study.

We are grateful to Lesley Williamson, Monica Munoz-Troncoso, Snehal Pandya and Emily Pickering (CHARIOT register and facilitator team); Mariam Jiwani, Rachel Veeravalli, Islam Saiful, Danielle Rose, Susie Gold, Rachel Nejade and Shehla Shamsuddin (Imperial College London student volunteers); Stefan McGinn-Summers, Neil Beckford, Inthushaa Indrakumar

For peer review only

For peer review only - http://bmjopen.bmj.com/site/about/guidelines.xhtml

and Kristina Lakey (Departmental administrative staff in AGE); Dinithi Perera (departmental manager); Heather McLellan-Young (project manager); Helen Ward, James McKeand, Geraint Price, Josip Car, Christina Atchison, Nicholas Peters, Aldo Faisal, and Jennifer Quint (investigator team contributing to CCRR survey design, development and improvement).

How patients were involved in the creation of this article

Older adult volunteers (60-80 years of age) from various social and cultural backgrounds provided feedback on the survey content. This feedback was incorporated into the survey design.

Conflicts of Interest

All authors have completed the ICMJE uniform disclosure form at www.icmje.org/coi_disclosure.pdf and declare: no support from any organisation for the submitted work; Lefkos T. Middleton reports research funding from Janssen, Novartis, Merck and Takeda, outside the submitted work.

Licence

The Corresponding Author has the right to grant on behalf of all authors and does grant on behalf of all authors, a worldwide licence to the Publishers and its licensees in perpetuity, in all forms, formats and media (whether known now or created in the future), to i) publish, reproduce, distribute, display and store the Contribution, ii) translate the Contribution into other languages, create adaptations, reprints, include within collections and create summaries, extracts and/or, abstracts of the Contribution, iii) create any other derivative work(s) based on the Contribution, iv) to exploit all subsidiary rights in the Contribution, v) the inclusion of electronic links from the Contribution to third party material where-ever it may be located; and, vi) licence any third party to do any or all of the above.

For peer review only

Abstract

Objectives: Physical inactivity is more common in older adults, is associated with social isolation and loneliness, and contributes to increased morbidity and mortality. We examined the effect of social restrictions, implemented to reduce transmission of COVID-19 in the UK (lockdown), on physical activity (PA) levels of older adults, and the demographic, lifestyle and social predictors of this change.

Design: Baseline analysis of a survey-based prospective cohort study

Setting: Adults enrolled in the Cognitive Health in Ageing Register for Investigational and Observational Trials (CHARIOT) cohort from GP practices in North West London were invited to participate from April to July 2020.

Participants: 6,219 cognitively healthy adults aged 50 to 92 years completed the survey.

Main outcome measures: Self-reported PA before and after lockdown, as measured by Metabolic Equivalent of Task (MET) minutes. Associations of PA with demographic, lifestyle and social factors, mood and frailty.

Results: Mean PA was significantly lower following lockdown, from 3,519 MET minutes/week to 3,185 MET minutes/week (p<0.001). After adjustment for confounders and pre-lockdown PA, lower levels of PA after lockdown were found in those who were over 85 years old (640 [95% CI: 246 to 1034] MET minutes/week less); were divorced or single (240 [95% CI: 120 to 360] MET minutes/week less); living alone (277 [95% CI: 152 to 402] MET minutes/week less); reported feeling lonely often (306 [95% CI: 60 to 552] MET minutes/week less); and showed symptoms of depression (1007 [95% CI: 1401 to 612] MET minutes/week less) compared to those aged 50-64 years, married, co-habiting, and not reporting loneliness or depression, respectively.

Conclusions and Implications: Markers of social isolation, loneliness and depression were associated with lower PA following lockdown in the UK. Targeted interventions to increase PA in older adults who identify as socially isolated, lonely or depressed should be considered.

Limitations:

- Survey responders identified predominantly as White/Caucasian background, which may limit the generalisability of the findings to other population groups
- Survey responders showed higher levels of physical activity than the general population

Strengths:

For peer review only - http://bmjopen.bmj.com/site/about/guidelines.xhtml

- Survey responses were obtained from over 6000 older adults
- Multivariable analyses were adjusted for confounders according to pre-determined causal pathways

For peer review only

For peer review only - http://bmjopen.bmj.com/site/about/guidelines.xhtml

1.0 Background and Rationale

Physical inactivity (PA) adversely affects older adults, with 60-70% of those aged over 75 years not sufficiently active for good health[1,2] as defined by meeting World Health Organization (WHO)[3] and UK[4] guidelines. From March until June 2020 in the UK, a national 'lockdown' was implemented to reduce exposure to, and transmission of, COVID-19. Although applied to the whole population, adults aged over 70 years and those with underlying health conditions at higher risk of severe COVID-19 disease were asked to follow more stringent social distancing measures. These included remaining at home where possible; avoiding social mixing in the community; avoiding physically interacting with friends and family; and avoiding public transport.[5]

Social isolation and loneliness in older adults, possibly exacerbated during lockdowns,[6] is associated with increases in morbidity and mortality, and also with increases in physical inactivity and sedentary time, as shown from subjective self-reporting and from accelerometer data.[7,8] Physical inactivity may therefore have a role in mediating the increased morbidity and mortality associated with social isolation.[9] We set up the CHARIOT COVID-19 Rapid Response study (CCRR) in April 2020 to monitor symptoms and the impact of the COVID-19 pandemic on various health and lifestyle factors, by repeat questionnaire survey of the Cognitive Health in Ageing Register for Investigational and Observational Trials (CHARIOT) members.

We hypothesised that imposed social restrictions would negatively impact on PA levels of older adults, and that change in PA after lockdown would be modified by certain demographic, lifestyle and social factors, with a focus on markers of social isolation and perceived loneliness. An awareness of the extent of, and predictors for, change in PA levels will aid our understanding of the impact of social isolation on the health of older adults, both with respect to pandemic-related lockdowns and social isolation itself.

2.0 Methods

2.1 CCRR survey

Study participants were recruited from the CHARIOT register, a cohort of over 40,000 cognitively healthy adult volunteers aged over 50 years, recruited from 172 GP surgeries across West and North London as part of a collaboration between regional GP practices and the School of Public Health, at Imperial College London.

For peer review only - http://bmjopen.bmj.com/site/about/guidelines.xhtml

This ongoing prospective cohort study was initiated in April 2020 with repeated questionnaire surveys conducted every six weeks. The CCRR baseline survey consists of questions related to basic demographics, diet, alcohol and smoking status, symptoms of COVID-19, functional activities, physical activity, sleep, frailty and mental health (supplementary file 1). For physical activity, the International Physical Activity Questionnaire (IPAQ) short-form was used,[10] asking respondents to document their weekly vigorous and moderate activity, walking and sitting time from the week prior to completing the survey; and for the week prior to implementation of social restriction measures. For assessing frailty, the 5-point FRAIL scale,[11,12] and for assessing mental health symptoms, the Hospital Anxiety and Depression (HADS) scale,[13] were used. A question on loneliness was used from the Imperial College Sleep Quality questionnaire; in turn adapted from the Pittsburgh Sleep Quality Index[14] and Centre for Epidemiologic Studies of Depression Scale[15], for work-free periods.

The survey was sent to 15,000 CHARIOT participants via email, with a subsequent 25,000 contacted by post. 7,320 participants responded and completed the survey. Of these respondents, 6,219 completed IPAQ data both before and after introduction of lockdown measures and were included in the final analysis. Data used in the present analysis were completed between 30th April and the 22nd July 2020.

2.2 Statistical analysis

All analyses were conducted using Stata version 16.1 (StataCorp 2019) and R.[16,17] Body Mass Index (BMI) was calculated as weight in kilograms divided by the square of height in metres and categorised according to standard WHO criteria. IPAQ data were cleaned according to the IPAQ data cleaning protocol,[18] and the Metabolic Equivalent of Task (MET) minutes per week, calculated for each activity and total activity (supplementary file 2). Paired t-tests were used to compare the distributions of mean PA levels pre- and post-lockdown.

Measures of association with explanatory variables were explored in univariable linear regression models for two outcomes: i) overall weekly MET minutes after introduction of lockdown and ii) the difference in overall weekly MET minutes before versus after the introduction of lockdown. Multivariable models were constructed for the outcome of MET minutes after lockdown, adjusting *a priori* each explanatory variable in turn for age, sex and ethnicity. Month of survey completion was also included in the model to account for seasonal changes, and the finding that physical activity after lockdown varied by month (supplementary file 2: figure 2). Weekly MET minutes before lockdown was also included in the model given its strong association with activity levels after lockdown, which remained significantly associated in all models. Denominators for each model vary according to the levels of

For peer review only - http://bmjopen.bmj.com/site/about/guidelines.xhtml

missingness in variables included in the models, which was low for most variables, except for BMI (unrecorded in 51.4% of participants).

A causal diagram was constructed using DAGitty[19] (supplementary file 2: figure 4) to aid adjustment for confounders in order to separate the overall causal effects of marital status, loneliness and living alone on physical activity. Additional multivariable models were then constructed based on the causal diagram for loneliness, adjusting for age, sex, ethnicity, household status, marital status, shielding status and frailty category. No further adjustment was necessary for marital status or household status. Residuals were plotted against fitted values to assess for outlying points and heteroskedasticity; and plots of Cook's distance and leverage against fitted values were examined to detect the presence of influential points.

3.0 Results

3.1 Participant characteristics

Of the 6,219 participants included in the present study, 55.4% were female, and the majority (55.3%) were aged 65-74 years with a mean age of 70 years. 93.7% of respondents classified themselves as being of white ethnic background, with 2.8% of Asian ethnic background, and only 0.7% of black African or Caribbean background. Approximately half of participants (48.6%) had a recorded height and weight, with a mean BMI of 25.3 kg/m^2. The majority of respondents were married (62.2%), co-habiting (72.8%) and retired (69.5%). Most respondents did not smoke (96.9%), drank alcohol (82.6%) and felt they ate a healthy diet (80.3%). 18.0% of respondents were classified as pre-frail, with 0.5% as frail and 26.2% reported that they were shielding at the time of the survey (table 1).

3.2 Physical activity before and after social distancing measures

Mean (SD) PA for participants prior to lockdown was 3,519 (2867) MET minutes/week. There was a significant reduction in mean MET minutes following implementation of lockdown to 3,186 (2673) MET minutes/week ($p<0.001$; table 2 & figure 1). 3,167 (50.9%) participants decreased their activity during lockdown by a mean (SD) of 1,957 (2025) MET minutes/week, 534 (8.6%) maintained the same level of activity, and 2,518 (40.5%) increased activity by a mean (SD) of 1,636 (1775) MET minutes/week. Mean sitting time increased by 276 MET minutes/week after lockdown (2,680) compared to before (2,404) (table 2).

5,762 (92.7%) participants achieved at least the minimum guidance of 600 MET minutes/week of activity, as defined by WHO,[3] prior to implementation of lockdown measures, slightly reducing to 5,672 (91.2%) following their introduction ($p<0.001$). 5,039 (81.0%) achieved 1,200 MET minutes/week before lockdown, with 4,904 (78.9%) achieving this after lockdown ($p<0.001$). Following lockdown, PA levels varied by month of survey completion, with the

For peer review only - http://bmjopen.bmj.com/site/about/guidelines.xhtml

highest levels in June and lowest levels in July. There was no significant difference between self-reported PA before lockdown by month of survey completion (supplementary file 2: table 1 & figures 2-3).

3.3 Predictors of physical activity after lockdown, and change from before lockdown

3.3.1 Demographic and lifestyle factors

Univariable linear regression models showed statistically significant associations with lower PA after lockdown in older age groups but no evidence of differences in the change from before lockdown between age groups ($p<0.001$ and $p=0.184$, respectively; figures 1 & 2). After multivariable adjustment for sex, ethnicity, month of survey completion and pre-lockdown physical activity there was evidence of significantly lower levels of PA with increasing age, with adults aged 85 years and over doing on average 640 (95% CI: 246 to 1034) MET minutes/week less than those aged 50-64 years (figure 3). There was no significant difference in PA after lockdown in males and females ($p=0.180$), but females on average exhibited a greater decline in PA from before lockdown than males (450 vs 189 MET minutes/week less respectively; $p<0.001$; figures 1 & 2). After multivariable adjustment, including age, there was only a small and borderline significant difference in PA after lockdown between gender (PA in males on average 108 MET minutes/week more than females; 95% CI: -1 to 216; figure 3). No significant associations were seen between PA after lockdown or change in PA according to ethnicity or employment status, before or after adjustment.

Lower levels of PA after lockdown were seen with increasing BMI category, in current smokers and in those reporting an unhealthy or worsening diet before and after adjustment (figure 1). After adjustment, a dose-response relationship was evident between lower PA and increasing BMI ($p=0.030$), with obese individuals doing 578 (95% CI: 324 to 832) MET minutes/week less than those of a healthy weight (figure 3). The denominator included in analyses of BMI was significantly lower than for other models, as BMI was unrecorded for 51.4% of participants. Current alcohol consumption was weakly associated with increased levels of PA in both univariable and multivariable models, with current drinkers reporting 145 MET minutes/week more than non-drinkers after adjustment (95% CI: 1 to 289; figures 2 & 3).

3.3.2 Associations with social isolation and loneliness

Participants who were divorced, single or widowed were, on average, less active after lockdown than those married or living with a partner (3,026 vs 3,262 MET minutes/week; $p=0.001$); and exhibited a greater decline in PA from before lockdown (540 vs 236 MET minutes/week less; $p<0.001$; figures 1 & 2). The association with PA after lockdown remained after adjustment, with those divorced, single or widowed doing on average 240 (95% CI: 120

For peer review only - http://bmjopen.bmj.com/site/about/guidelines.xhtml

to 360) MET minutes/week less (figure 3). Participants living alone were also less active than those co-habiting and showed greater reductions in PA from before lockdown. After adjustment for confounders and PA before lockdown, those living alone were doing 277 (95% CI: 152 to 402) MET minutes/week less than those co-habiting (figure 3).

Significant associations were seen between PA after lockdown and frequency of loneliness, with those 'often' experiencing loneliness achieving 2,938 MET minutes/week compared with 3,284 MET minutes/week in those 'never' experiencing loneliness (p=0.024; figure 1). Greater declines in PA from before lockdown were also seen with increasing loneliness (figure 2). After adjustment, PA after lockdown was significantly lower for those with increased frequency of loneliness (figure 3). After full adjustment including, in addition, household status, shielding status and frailty category, those experiencing loneliness 'often' reported 306 (95% CI: 60 to 552) MET minutes/week less activity than those 'never' lonely (supplementary file 2: table 4).

Significantly lower physical activity levels were recorded in those shielding and in participants categorised as pre-frail or frail (both p<0.001; figure 1). Larger declines in PA from before lockdown were seen in those shielding compared to those not shielding (588 vs 243 MET minutes/week less; p<0.001), but there was no significant difference in change in PA according to frailty category (p=0.389; figure 2). After adjustment, frail participants were doing 926 (95% CI: 189 to 1,663) MET minutes less on average than those classed as robust (figure 3). Participants who were shielding were doing an average of 290 (95% CI: 163 to 417) MET minutes/week less than those not shielding (figure 3).

3.3.3 Associations with depression and anxiety

Symptoms of depression were associated with lower levels of PA during lockdown, with those meeting the criteria for depression reporting 2,450 MET minutes/week compared to 3,195 MET minutes/week in those with normal scores (p<0.001; figure 1). There was no strong association with anxiety scores. Mean change in PA from before lockdown was associated with both depression and, in contrast to absolute PA levels, with anxiety scores. Participants with depression reported 1,450 MET minutes/week less on average after lockdown compared with before, while those with normal scores reported 293 MET minutes/week less (p<0.001). Similarly, in those with anxiety, PA reduced by 836 MET minutes/week compared to 312 MET minutes/week in those with normal scores (p=0.004; figure 2).

After adjustment, those meeting the criteria for depression on the HADS scale had significantly lower PA levels than those with normal scores, doing on average 1,007 (95% CI: 1401 to 612) MET minutes/week less (figure 3). There remained no statistically significant association between anxiety score and physical activity after adjustment.

For peer review only - http://bmjopen.bmj.com/site/about/guidelines.xhtml

4.0 Discussion

4.1 Main findings

Data from the CCRR study show that participants experienced, on average, a significant decrease in PA after the introduction of lockdown in the UK when compared with before, together with an increase in sitting time. When adjusted for age, sex, ethnicity, month of survey completion and baseline physical activity, factors strongly associated with a reduction in PA include; increased age, increased BMI, frailty, current smoking, and a change to a less healthy diet. Factors associated with social isolation were also significantly associated with a reduction in PA: those divorced, single or widowed, living alone, shielding or reporting increased frequency of loneliness did significantly less PA after lockdown. Furthermore, a strong association was also seen with lower PA during lockdown in those with depression, but not for those with anxiety.

4.2 The effect of lockdown on physical activity

There was a reduction in PA in over half of our participants, and a decrease in mean levels of PA by 333 MET minutes/week following the introduction of lockdown measures in the UK. This was accompanied by an increase in sitting time by 276 minutes per week, an adverse finding given the adverse health impacts associated with increased sedentary and sitting time.[20] These findings correlate with other studies from the UK (a decrease in 25% of adults aged over 20 years following lockdown),[21] Spain[22] and China,[23] and from a global survey collected in 8 different languages,[24] despite the differences in outdoor exercise permissions between countries. Reductions in PA may impact disproportionately across society. We found that increasing age associated with a reduction in PA after lockdown, corresponding with that seen in Japan, with a 26.5% (65 minutes) decrease in total physical activity in adults aged 65 to 84.[25] The UK Active Lives Survey found a 7.3% reduction in the proportion of active adults aged 55-74 years, from 63% to 56%, during the pandemic, and a 6.6% reduction in those aged 75 years and above, from 42% to 35%.[26] A self-reported study in the UK found that those with a diagnosis of obesity, hypertension, lung disease, depression or a disability were more likely to reduce PA during lockdown.[21]

4.3 Social relationships, loneliness, and physical activity

Individuals for whom social engagement was more likely to be restricted, such as those who were shielding, divorced, single, widowed, or living alone, were more likely to have lower levels of PA after lockdown, and to have declined to a greater extent. Similarly, those who subjectively reported feeling lonely were more likely to have lower PA levels, and

For peer review only - http://bmjopen.bmj.com/site/about/guidelines.xhtml

greater declines from before lockdown. These associations remained significant after multivariable adjustment.

Associations between health behaviours, including PA, and social relationships have been noted previously. Data from the English Longitudinal Study of Ageing (ELSA) showed that socially isolated respondents were less likely to report healthy diets, and more likely to smoke.[7] Crucially, they showed reduced activity counts in socially isolated individuals (measured by accelerometer) in a sample of adults older than 50 years,[8] and reduced self-reported moderate to vigorous physical activity.[7] This is particularly important given that isolated and lonely individuals are at an increased risk of morbidity and mortality from cardiovascular events, with the majority of this association mediated by risk factors which include physical inactivity.[27] Fixed effect models from the ELSA cohort show that social disengagement, domestic isolation and loneliness are associated with measures of poorer physical performance, and although they appear to be independent of physical activity, may still be associated along the causal pathway.[28] Studies of spousal pairs found that both men and women in married couples had greater levels of PA than their single counterparts,[29] and changes in PA are positively associated with changes in the PA of a spouse.[30] Increasing PA is associated with larger,[31,32] more diverse[33] and more heterogenous (in terms of PA) social networks, and having more physically active people in a social network is associated with being more active.[34]

The interaction between social relationships and PA levels may be bi-directional. Levels of PA are influenced by multiple factors at different levels, including individual (psychological, genetic); interpersonal (social networks); environmental (social, built, natural); and regional or global determinants.[35] Social networks might influence PA through social support for individuals to take up and maintain activity, but also by regulating social norms, and associating PA with social connections or attachments.[36] There may also be increased opportunities for PA[34] when social networks are present.

4.4 Mood, health behaviours and physical activity

In those reporting symptoms of depression, there were significantly lower levels of PA and a significant decrease in activity when compared to before lockdown. These findings correlate with those from the UK,[37] Australia,[38] and Spain,[39] which found inverse associations between physical activity levels and poor mental health. Similarly, a cross sectional study of Brazilian adults who were self-isolating found lower odds of symptoms of anxiety or depression in those who were performing over 30 or 15 minutes per day of moderate or vigorous activity respectively, and higher odds in those with prolonged sedentary time over 10 hours.[40] The associations between PA and mental health are well

For peer review only - http://bmjopen.bmj.com/site/about/guidelines.xhtml

known, with positive impacts on wellbeing,[41] and reduced incidence and severity of symptoms of mental ill-health.[42–44] Therefore, these findings are unsurprising, although the interaction between PA and reduced markers of mental ill-health in older adults may be bi-directional. Moreover, social isolation and loneliness may mediate some of this effect: previous data from the CCRR cohort showed an interaction between social isolation, loneliness, and female gender with worsening depression and anxiety over lockdown.[45] We found no statistically significant difference in PA during lockdown with anxiety symptoms, at odds with previous studies.[37] However, the trajectory of anxiety symptoms is not known, and it is not clear whether anxiety symptoms pre-dated the introduction of lockdown.

4.5 Health behaviours and physical activity

A decrease in PA was associated with other detrimental health behaviours, including unhealthy diet and smoking. A similar tendency of clustering of unhealthy behaviours during the COVID-19 pandemic was noted in a cohort of patients in Spain with type 2 diabetes mellitus, who showed an increase in sugary foods and snack consumption alongside an increase in sitting time, and a decrease in time spent walking or doing moderate physical activity during lockdown when compared to beforehand.[46] That detrimental health behaviours might coincide in response to lockdown shows the importance of targeted interventions for certain groups. Interestingly, alcohol consumption was seen to be a protective factor in our cohort, and this does not tie with other findings on the negative associations with increased alcohol use during the COVID-19 pandemic.[47] This may be due to the specific demographic features of our cohort, but the possibility of alcohol consumption being associated with social interaction in this group cannot be excluded.

4.6 Limitations

This study has several limitations which may impact the generalisability of our findings. First, the CCRR cohort appear more physically active than the general population. 90% of participants in CCRR achieved minimum UK [4] and WHO [3] guidance, both before and following lockdown. Over 78% achieved double this amount, and mean levels of PA were at least five times greater than the minimum recommendation. In contrast, only 61% of UK adults aged 55-74 years achieve minimum recommended levels.[2] Despite this, CCRR participants may still not be active enough for major health gains. A 2016 systematic review and meta-analysis suggested that optimal risk reduction for breast and colorectal cancer, diabetes, ischaemic heart disease and stroke events were obtained from physical activity at 3000-4000 MET minutes per week.[48]

Second, there are differences in demography between the CCRR cohort and the general population of the UK, which may explain the higher levels of PA we observed. 93% of CCRR

For peer review only - http://bmjopen.bmj.com/site/about/guidelines.xhtml

respondents identify as white/Caucasian ethnicity. The Active Lives Survey demonstrated a difference in those achieving minimum activity levels in White British individuals (65%) and those from Black (58%) and Asian (54%) ethnicities.[2] Third, the CCRR survey relies on self-report, using the short form IPAQ. IPAQ data is well validated across diverse participants up to the age of 65 years [10] and a study of the performance of the IPAQ in older Japanese adults demonstrated adequate validity.[49] However, results from self-reporting tools for PA only weakly correlate with those from objective measures, such as accelerometers and pedometers.[50–53] Finally, recall bias and seasonal changes in physical activity may also have impacted on the results. The CCRR survey was collected in April-July 2020, with participants asked to recall PA levels in the week before lockdown, which over time may become less reliable. However, no significant differences were found in the mean PA levels reported before lockdown according to month of survey completion and although there were apparent differences in PA during lockdown by month, we were able to adjust for this in multivariable models. The CCRR prospective cohort study is ongoing, with follow-up questionnaires sent to participants at regular intervals. When complete this will allow for long-term impacts to be measured, accounting for seasonal variation.

4.7 Conclusions

Findings from our CCRR study suggest a significant decline in average physical activity levels in older adults following the introduction of lockdown measures during the COVID-19 pandemic. Lower activity levels after lockdown were strongly linked to older age, and to those with objective markers of social isolation, subjective feelings of loneliness and symptoms of depression. Strategies and targeted interventions to increase and sustain PA levels in older adults are needed to mitigate the adverse health impacts not only of COVID-19 related lockdowns, but of social isolation in general, and should consider social relationships in their design and implementation.

5.0 Summary boxes

<u>What is already known on this topic</u>

-Physical inactivity adversely affects older adults: almost two-thirds of adults over 75 years old are not sufficiently physically active for good health

-Social isolation and loneliness are associated with increased morbidity and mortality, and decreased physical activity; lockdowns for Covid-19, although crucial, may exacerbate this

<u>What this study adds</u>

For peer review only - http://bmjopen.bmj.com/site/about/guidelines.xhtml

-Physical activity decreased in older adults following implementation of lockdown measures in the UK
-Those with factors suggesting increased social isolation, loneliness and depression were particularly susceptible to lower levels of physical activity after lockdown
-Interventions designed to increase physical activity in older adults should take account of social relationships in their design and implementation, and there is a case for specific resources to help protect socially isolated individuals during pandemic-related lockdowns

6.0 References

1 NHS Digital. Health Survey for England 2016 Physical activity in adults. 2017 https://files.digital.nhs.uk/publication/m/3/hse16-adult-phy-act.pdf.

2 Sport England. Active Lives Adult Survey November 2018/19 Report. 2020 https://sportengland-production-files.s3.eu-west-2.amazonaws.com/s3fs-public/2020-04/Active Lives Adult November 18-19 Report..pdf?BhkAy2K28pd9bDEz_NuisHl2ppuqJtpZ.

3 World Health Organization. Global Recommendations on Physical Activity for Health. 2010 https://www.who.int/publications/i/item/9789241599979.

4 Davies SC, Atherton F, McBride M, Calderwood C. UK Chief Medical Officers' Physical Activity Guidelines. 2019.

5 Gov.uk. UK Government COVID-19 guidance. .

6 Wu B. Social isolation and loneliness among older adults in the context of COVID-19: a global challenge. *Glob Heal Res Policy* 2020; **5**: 27.

7 Kobayashi LC, Steptoe A. Social Isolation, Loneliness, and Health Behaviors at Older Ages: Longitudinal Cohort Study. *Ann Behav Med* 2018; **52**: 582–93.

8 Schrempft S, Jackowska M, Hamer M, Steptoe A. Associations between social isolation, loneliness, and objective physical activity in older men and women. *BMC Public Health* 2019; **19**: 74.

9 Elovainio M, Hakulinen C, Pulkki-Råback L, *et al.* Contribution of risk factors to excess mortality in isolated and lonely individuals: an analysis of data from the UK Biobank cohort study. *Lancet Public Heal* 2017. DOI:10.1016/S2468-2667(17)30075-0.

10 Craig CL, Marshall AL, Sjöström M, *et al.* International physical activity questionnaire: 12-country reliability and validity. *Med Sci Sports Exerc* 2003; **35**: 1381–95.

11 Gleason LJ, Benton EA, Alvarez-Nebreda ML, Weaver MJ, Harris MB, Javedan H. FRAIL Questionnaire Screening Tool and Short-Term Outcomes in Geriatric Fracture Patients. *J Am Med Dir Assoc* 2017. DOI:10.1016/j.jamda.2017.07.005.

12 Woo J, Yu R, Wong M, Yeung F, Wong M, Lum C. Frailty screening in the community using the FRAIL scale. *J Am Med Dir Assoc* 2015. DOI:10.1016/j.jamda.2015.01.087.

13 Zigmond AS, Snaith RP. The Hospital Anxiety and Depression Scale. *Acta Psychiatr Scand* 1983. DOI:10.1111/j.1600-0447.1983.tb09716.x.

For peer review only - http://bmjopen.bmj.com/site/about/guidelines.xhtml

For peer review only

14 Buysse DJ, Reynolds CF, Monk TH, Berman SR, Kupfer DJ. The Pittsburgh sleep quality index: A new instrument for psychiatric practice and research. *Psychiatry Res* 1989. DOI:10.1016/0165-1781(89)90047-4.

15 Radloff LS. The CES-D Scale: A Self-Report Depression Scale for Research in the General Population. *Appl Psychol Meas* 1977. DOI:10.1177/014662167700100306.

16 R Core Team. R: A language and environment for statistical computing. R Found. Stat. Comput. 2019.

17 Viechtbauer W. Conducting meta-analyses in R with the metafor. *J Stat Softw* 2010; **36**: 1–48.

18 IPAQ scoring protocol - International Physical Activity Questionnaire. https://sites.google.com/site/theipaq/scoring-protocol (accessed Dec 1, 2020).

19 Textor J, van der Zander B, Gilthorpe MS, Liśkiewicz M, Ellison GT. Robust causal inference using directed acyclic graphs: The R package 'dagitty'. *Int J Epidemiol* 2016. DOI:10.1093/ije/dyw341.

20 Ekelund U, Tarp J, Steene-Johannessen J, *et al.* Dose-response associations between accelerometry measured physical activity and sedentary time and all cause mortality: Systematic review and harmonised meta-analysis. *BMJ* 2019. DOI:10.1136/bmj.l4570.

21 Rogers NT, Waterlow NR, Brindle H, *et al.* Behavioral Change Towards Reduced Intensity Physical Activity Is Disproportionately Prevalent Among Adults With Serious Health Issues or Self-Perception of High Risk During the UK COVID-19 Lockdown . Front. Public Heal. . 2020; **8**: 526.

22 Castañeda-Babarro A, Arbillaga-Etxarri A, Gutiérrez-Santamaría B, Coca A. Physical Activity Change during COVID-19 Confinement. *Int J Environ Res Public Health* 2020; **17**: 1–10.

23 Wang X, Lei SM, Le S, *et al.* Bidirectional Influence of the COVID-19 Pandemic Lockdowns on Health Behaviors and Quality of Life among Chinese Adults. DOI:10.3390/ijerph17155575.

24 Ammar A, Brach M, Trabelsi K, *et al.* Effects of COVID-19 Home Confinement on Eating Behaviour and Physical Activity: Results of the ECLB-COVID19 International Online Survey. *Nutrients* 2020; **12**. DOI:https://dx.doi.org/10.3390/nu12061583.

25 Yamada M, Kimura Y, Ishiyama D, *et al.* Effect of the COVID-19 Epidemic on Physical Activity in Community-Dwelling Older Adults in Japan: A Cross-Sectional Online Survey. *J Nutr Health Aging* 2020; : 1–3.

26 Active Lives Adult Survey Coronavirus (Covid-19) Report. 2020 https://sportengland-production-files.s3.eu-west-2.amazonaws.com/s3fs-public/2020-10/Active Lives Adult May 19-20 Coronavirus Report.pdf?2L6TBVV5UvCGXb_VxZcWHcfFX0_wRal7 (accessed Feb 8, 2021).

27 Hakulinen C, Pulkki-Råback L, Virtanen M, Jokela M, Kivimäki M, Elovainio M. Social isolation and loneliness as risk factors for myocardial infarction, stroke and mortality: UK Biobank cohort study of 479 054 men and women. *Heart* 2018. DOI:10.1136/heartjnl-2017-312663.

28 Philip KEJ, Polkey MI, Hopkinson NS, Steptoe A, Fancourt D. Social isolation, loneliness and physical performance in older-adults: fixed effects analyses of a cohort study. *Sci Rep* 2020; **10**: 13908.

For peer review only - http://bmjopen.bmj.com/site/about/guidelines.xhtml

29 Pettee KK, Brach JS, Kriska AM, *et al.* Influence of marital status on physical activity levels among older adults. *Med Sci Sports Exerc* 2006; **38**: 541–6.

30 Cobb LK, Godino JG, Selvin E, Kucharska-Newton A, Coresh J, Koton S. Spousal influence on physical activity in middle-aged and older adults. *Am J Epidemiol* 2016; **183**: 444–51.

31 Marquez B, Elder JP, Arredondo EM, Madanat H, Ji M, Ayala GX. Social network characteristics associated with health promoting behaviors among Latinos. *Heal Psychol* 2014; **33**: 544–53.

32 Tamers SL, Okechukwu C, Allen J, *et al.* Are social relationships a healthy influence on obesogenic behaviors among racially/ethnically diverse and socio-economically disadvantaged residents? *Prev Med (Baltim)* 2013; **56**: 70–4.

33 Legh-Jones H, Moore S. Network social capital, social participation, and physical inactivity in an urban adult population. *Soc Sci Med* 2012; **74**: 1362–7.

34 Mötteli S, Dohle S. Egocentric social network correlates of physical activity. *J Sport Heal Sci* 2020; **9**: 339–44.

35 Bauman AE, Reis RS, Sallis JF, *et al.* Correlates of physical activity: Why are some people physically active and others not? Lancet. 2012. DOI:10.1016/S0140-6736(12)60735-1.

36 McNeill LH, Kreuter MW, Subramanian S V. Social Environment and Physical activity: A review of concepts and evidence. *Soc Sci Med* 2006; **63**: 1011–22.

37 Jacob L, Tully MA, Barnett Y, *et al.* The relationship between physical activity and mental health in a sample of the UK public: A cross-sectional study during the implementation of COVID-19 social distancing measures. *Ment Health Phys Act* 2020; **19**: 100345.

38 Stanton R, To QG, Khalesi S, *et al.* Depression, Anxiety and Stress during COVID-19: Associations with Changes in Physical Activity, Sleep, Tobacco and Alcohol Use in Australian Adults. *Int J Environ Res Public Health* 2020; **17**: 4065.

39 López-Bueno R, Calatayud J, Ezzatvar Y, *et al.* Association Between Current Physical Activity and Current Perceived Anxiety and Mood in the Initial Phase of COVID-19 Confinement . Front. Psychiatry . 2020; **11**: 729.

40 Schuch FB, Bulzing RA, Meyer J, *et al.* Associations of moderate to vigorous physical activity and sedentary behavior with depressive and anxiety symptoms in self-isolating people during the COVID-19 pandemic: A cross-sectional survey in Brazil. *Psychiatry Res* 2020; **292**: 113339.

41 Penedo FJ, Dahn JR. Exercise and well-being: A review of mental and physical health benefits associated with physical activity. Curr. Opin. Psychiatry. 2005. DOI:10.1097/00001504-200503000-00013.

42 Chekroud SR, Gueorguieva R, Zheutlin AB, *et al.* Association between physical exercise and mental health in 1·2 million individuals in the USA between 2011 and 2015: a cross-sectional study. *The Lancet Psychiatry* 2018. DOI:10.1016/S2215-0366(18)30227-X.

43 McDowell CP, Dishman RK, Gordon BR, Herring MP. Physical Activity and Anxiety: A Systematic Review and Meta-analysis of Prospective Cohort Studies. Am. J. Prev. Med. 2019; **57**: 545–56.

44 Schuch FB, Vancampfort D, Firth J, *et al.* Physical activity and incident depression: A

For peer review only - http://bmjopen.bmj.com/site/about/guidelines.xhtml

meta-analysis of prospective cohort studies. *Am J Psychiatry* 2018. DOI:10.1176/appi.ajp.2018.17111194.

45 Robb CE, de Jager CA, Ahmadi-Abhari S, *et al.* Associations of Social Isolation with Anxiety and Depression During the Early COVID-19 Pandemic: A Survey of Older Adults in London, UK. *Front Psychiatry* 2020. DOI:10.3389/fpsyt.2020.591120.

46 Ruiz-Roso MB, Knott-Torcal C, Matilla-Escalante DC, *et al.* COVID-19 Lockdown and Changes of the Dietary Pattern and Physical Activity Habits in a Cohort of Patients with Type 2 Diabetes Mellitus. *Nutrients* 2020; **12**. DOI:10.3390/nu12082327.

47 Sallie SN, Ritou V, Bowden-Jones H, Voon V. Assessing international alcohol consumption patterns during isolation from the COVID-19 pandemic using an online survey: highlighting negative emotionality mechanisms. *BMJ Open* 2020; **10**: e044276.

48 Kyu HH, Bachman VF, Alexander LT, *et al.* Physical activity and risk of breast cancer, colon cancer, diabetes, ischemic heart disease, and ischemic stroke events: systematic review and dose-response meta-analysis for the Global Burden of Disease Study 2013. *BMJ* 2016; **354**: i3857.

49 Tomioka K, Iwamoto J, Saeki K, Okamoto N. Reliability and validity of the international physical activity questionnaire (IPAQ) in elderly adults: The Fujiwara-kyo study. *J Epidemiol* 2011. DOI:10.2188/jea.JE20110003.

50 Prince SA, Adamo KB, Hamel ME, Hardt J, Connor Gorber S, Tremblay M. A comparison of direct versus self-report measures for assessing physical activity in adults: a systematic review. *Int J Behav Nutr Phys Act* 2008; **5**: 56.

51 Lee PH, Macfarlane DJ, Lam TH, Stewart SM. Validity of the international physical activity questionnaire short form (IPAQ-SF): A systematic review. Int. J. Behav. Nutr. Phys. Act. 2011; **8**: 115.

52 Cleland C, Ferguson S, Ellis G, Hunter RF. Validity of the International Physical Activity Questionnaire (IPAQ) for assessing moderate-to-vigorous physical activity and sedentary behaviour of older adults in the United Kingdom. *BMC Med Res Methodol* 2018; **18**. DOI:10.1186/s12874-018-0642-3.

53 Kowalski K, Rhodes R, Naylor P-J, Tuokko H, MacDonald S. Direct and indirect measurement of physical activity in older adults: a systematic review of the literature. *Int J Behav Nutr Phys Act* 2012; **9**: 148.

For peer review only

Participant characteristic		Total	Percent
**Gender**	Female	3,445	55.4%
	Male	2,770	44.5%
	Prefer not to say	4	0.1%
**Age (years)**	Mean (SD)	69.9 (7.3)	
	Median (IQR)	70 (66-74)	
	Range	50 - 92	
	50-64	1,212	19.5%
	65-74	3,440	55.3%
	75-84	1,394	22.4%
	85+	127	2.0%
	Missing	46	0.7%
**Ethnicity**	**White**	**5,825**	**93.7%**
	English/Welsh/Scottish/Northern Irish/British	5,143	82.7%
	Any other white background	536	8.6%
	Irish	146	2.3%
	**Mixed/multiple ethnic groups**	**99**	**1.6%**
	White and Black African	10	0.2%
	White and Asian	33	0.5%
	White and Black Caribbean	7	0.1%
	Any other mixed/multiple ethnic background	49	0.8%
	**Asian/Asian British**	**174**	2.8%
	Indian	91	1.5%
	Pakistani	12	0.2%
	Bangladeshi	2	0.0%
	Chinese	32	0.5%
	Any other Asian background	37	0.6%
	**Black/African/Caribbean/Black British**	**43**	**0.7%**
	African	13	0.2%
	Caribbean	21	0.3%
	Any other Black/African/Caribbean/Black British	9	0.1%
	**Other ethnic group**	**64**	**1.0%**
	Arab	17	0.3%
	Any other ethnic group	47	0.8%
	**Prefer not to say**	**14**	**0.2%**
**Body Mass Index (BMI) (Kg/m2)**	Mean (SD)	25.3 (5.1)	
	Median (IQR)	24.4 (22.2-27.1)	
	<18.5 (underweight range)	61	1.0%
	18.5-24.9 (healthy weight)	1,644	26.4%
	25.0-29.9 (overweight)	962	15.5%
	>=30.0 (obese range)	358	5.8%
	Missing data	3,194	51.4%
**Shielding at time of questionnaire**	No	4,591	73.8%
	Yes	1,628	26.2%
**Marital status**	Married	3,869	62.2%
	Single	789	12.7%
	Widowed	601	9.7%
	Divorced	595	9.6%

For peer review only

	Living with a partner	365	5.9%
**Living arrangements**	Co-habiting	4,530	72.8%
	Living alone	1,689	27.2%
**Employment**	Retired	4,322	69.5%
	Continuing to work in your usual job; at home	1,101	17.7%
	None of the above	201	3.2%
	Furloughed	197	3.2%
	Continuing to work in your usual job and leave home for your job	141	2.3%
	A key worker	96	1.5%
	Had to close your business due to COVID-19	70	1.1%
	Lost my job due to the lockdown	42	0.7%
	Unemployed	36	0.6%
	A student	13	0.2%
**Current smoker**	No	6,027	96.9%
	Yes	192	3.1%
**Alcohol intake**	No	1,083	17.4%
	Yes	5,136	82.6%
**Diet**	No change from usual - already had a healthy diet	4,991	80.3%
	My diet has become more healthy	715	11.5%
	My diet was healthy before but has got worse since lockdown	312	5.0%
	No change from usual - my diet isn't very healthy	201	3.2%
**FRAIL scale**	Robust	5,055	81.3%
	Pre-frail	1,117	18.0%
	Frail	34	0.5%
	Missing	13	0.2%
**HADS (depression score)**	Normal (0-7)	4,658	74.9%
	Borderline (8-10)	312	5.0%
	Abnormal (11-21)	116	1.9%
	Missing	1,133	18.2%
**HADS (anxiety score)**	Normal (0-7)	4,335	69.7%
	Borderline (8-10)	486	7.8%
	Abnormal (11-21)	265	4.3%
	Missing	1133	18.2%
	**Total participants**	**6,219**	

For peer review only

Physical activity type		Before	During	p value for difference
**Vigorous activity**	Mean (SD) minutes/week	145 (276)	135 (253)	0.004
	Median (IQR) minutes/week	40 (0 - 180)	10 (0 - 180)	
**Moderate activity (minutes/week)**	Mean (SD) minutes/week	292 (430)	245 (374)	<0.001
	Median (IQR) minutes/week	120 (0 - 360)	120 (0-360)	
**Walking (minutes/week)**	Mean (SD) minutes/week	462 (460)	403 (408)	<0.001
	Median (IQR) minutes/week	360 (150 - 630)	315 (150 - 525)	
**Sitting (minutes/week) ***	Mean (SD) minutes/week	2404 (1137)	2680 (1181)	<0.001
	Median (IQR) minutes/week	2100 (1680 - 2940)	2520 (1680 - 3360)	
**MET minutes/week**	Mean (SD) minutes/week	3519 (2867)	3185 (2673)	<0.001
	Median (IQR) minutes/week	2772 (1386 - 4650)	2440 (1386 - 4185)	

For peer review only

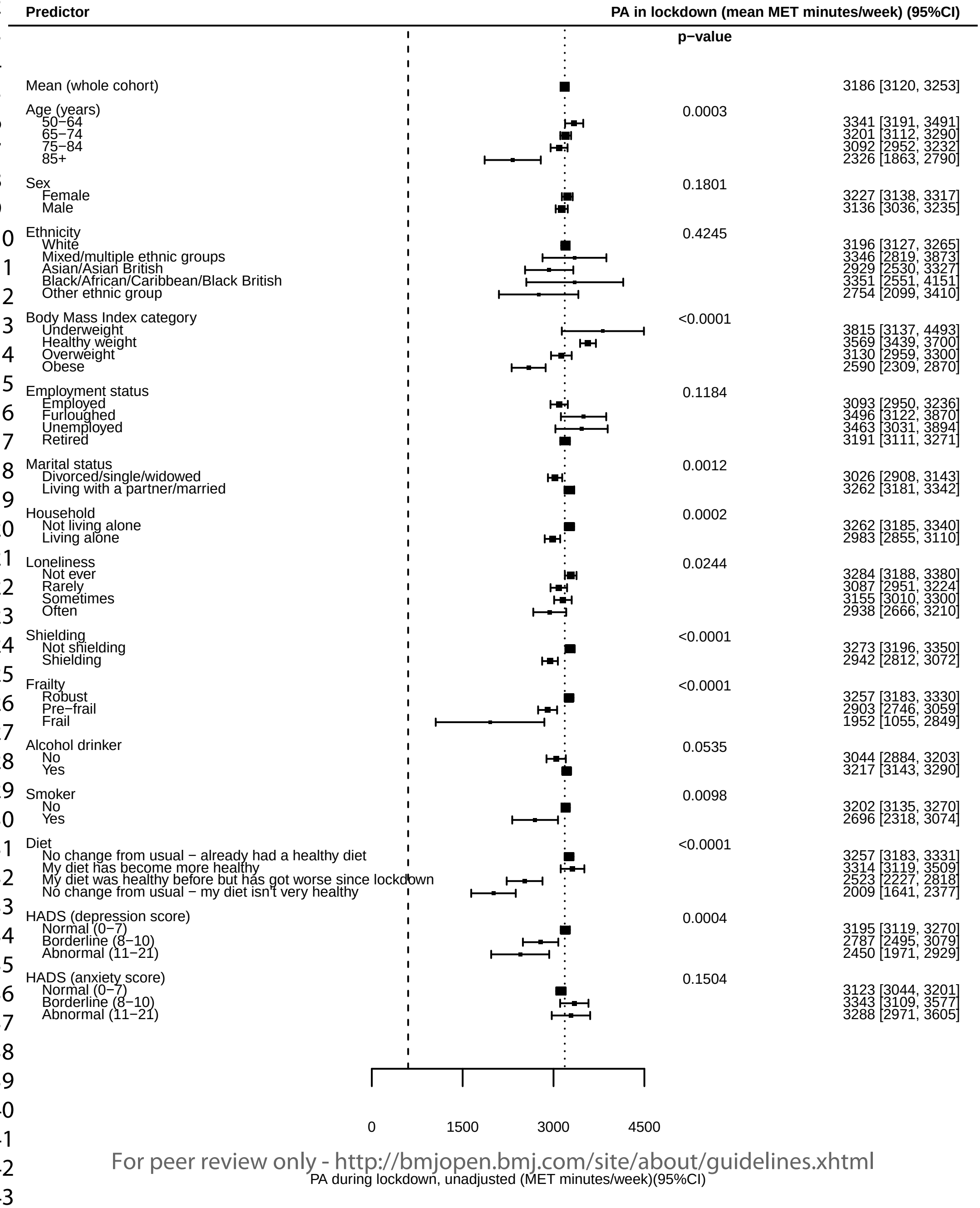

For peer review only - http://bmjopen.bmj.com/site/about/guidelines.xhtml

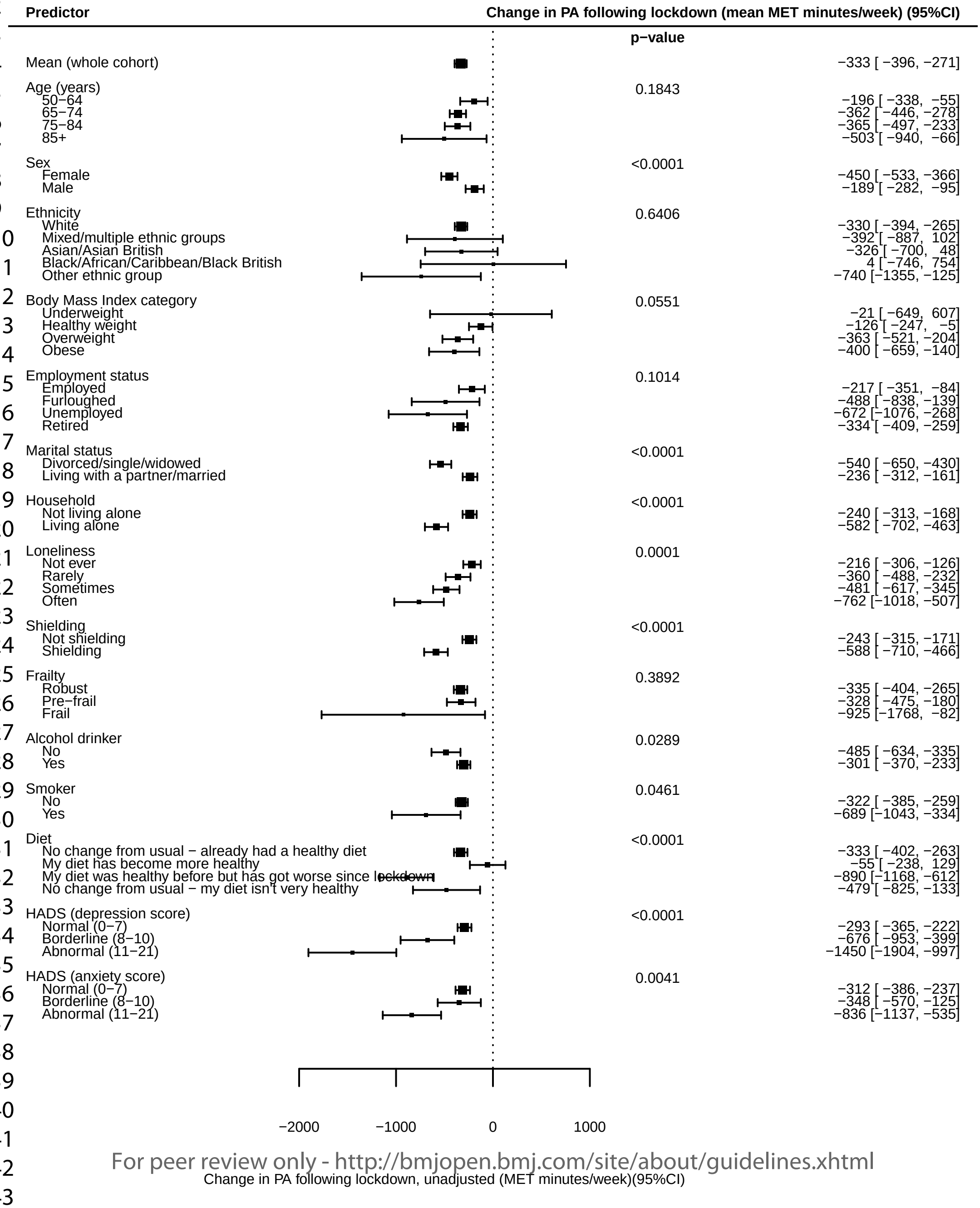

For peer review only - http://bmjopen.bmj.com/site/about/guidelines.xhtml

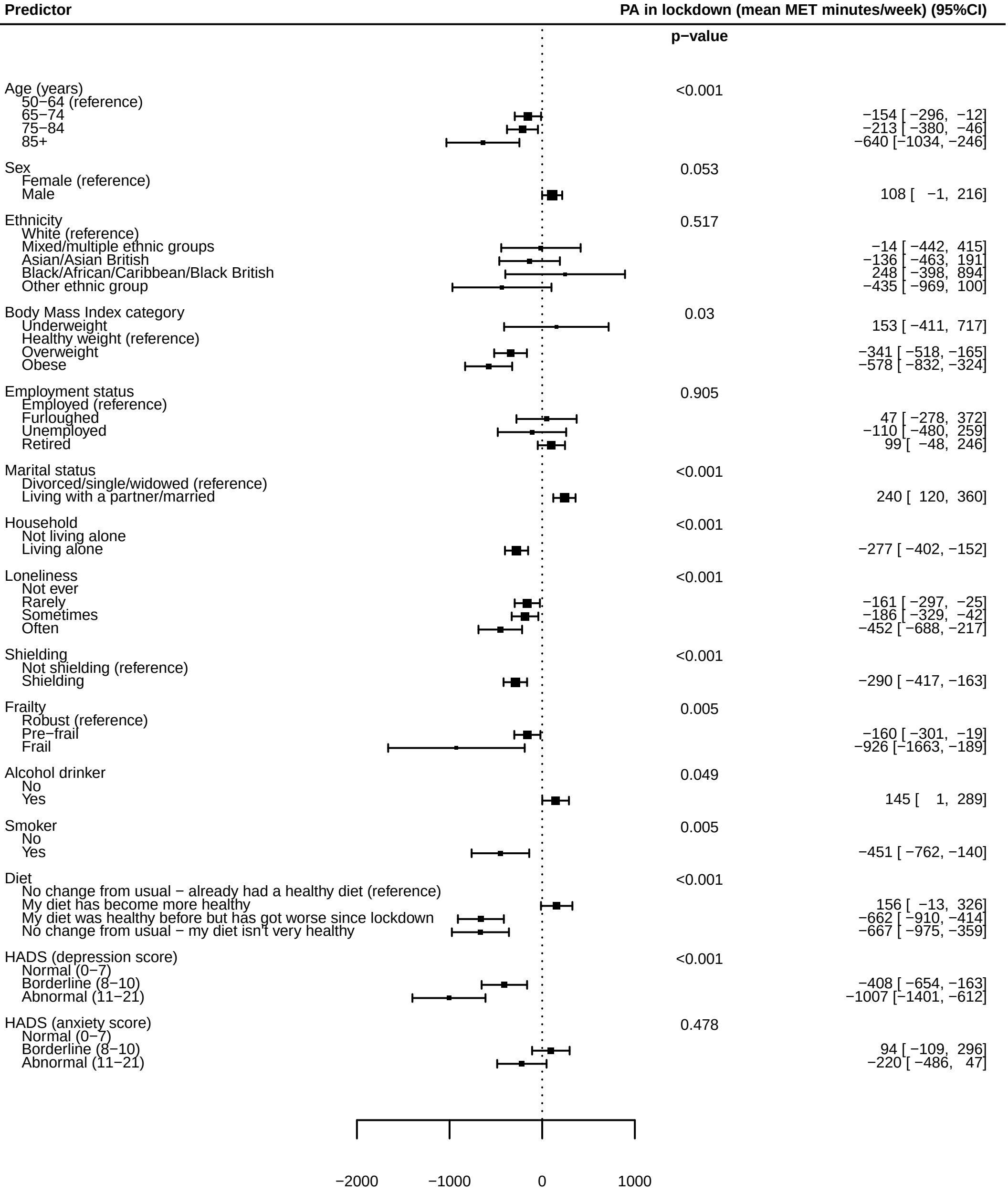

For peer review only - http://bmjopen.bmj.com/site/about/guidelines.xhtml

Multivariable associations with physical activity during lockdown (MET minutes/week)(95%CI)

Table and figure captions

Table 1: Participant characteristics for 6,219 participants with complete data on physical activity; HADS – Hospital Anxiety and Depression Score

Table 2: Physical activity and sitting time for recipients before and following introduction of lockdown measures. Data presented as minutes per week with both mean (standard deviation) and median (interquartile range) shown. p-values from paired t-test; *denominator 6,023; MET - Metabolic Equivalent of Task

Figure 1: Forest plot of unadjusted univariable associations with physical activity (PA) during lockdown. Data presented as mean MET minutes/week +/- 95% confidence interval. Heavy dashed line – 600 MET minutes/week (WHO minimal physical activity guideline for adults); light dashed line – mean MET minutes for the whole cohort. See also supplementary file 2: table 2; HADS – Hospital Anxiety and Depression Score; MET – Metabolic Equivalent of Task; PA – Physical Activity; WHO – World Health Organization

Figure 2: Forest plot of unadjusted mean change in physical activity (PA) for all variables (mean MET minutes/week +/- 95% confidence interval). Negative values indicate a decline in activity after lockdown compared to before lockdown. See also supplementary file 2: table 2; HADS – Hospital Anxiety and Depression Score; MET – Metabolic Equivalent of Task; PA – Physical Activity

Figure 3: Forest plot of multivariable associations with physical activity after lockdown, adjusted for age, sex, ethnicity, month of year of survey completion and baseline physical activity. Data presented as mean MET minutes/week +/- 95% confidence interval, compared to the reference group, with negative values indicating lower physical activity than the reference. See also supplementary file 2: table 3; HADS – Hospital Anxiety and Depression Score; MET – Metabolic Equivalent of Task; PA – Physical Activity

For peer review only

CHARIOT COVID-19 Rapid Response (CCRR) Study

Baseline Survey

Please answer all the questions in this survey before submitting it. Follow the prompts for those questions that are not applicable to you.

Symptoms

Q1. In the last week, have you had a cough?

<1> No

<2> Yes

Q2. In the last week, have you experienced unusual shortness of breath (difficulty breathing) compared to what's normal for you?

<1> No

<2> Yes, but it did not affect my normal activities

<3> Yes, it did affect my normal activities (eg walking short distances)

<4> Yes, even when I was sitting or lying down

Q3. In the last week, have you had a fever (feeling too hot) and did you take your temperature?

<1> I have NOT felt feverish

<2> I have felt feverish but did not check my temperature

<3> I felt feverish and my temperature was equal to, or BELOW 38 degrees Celcius

<4> I felt feverish and my temperature measured ABOVE 38 degrees Celcius

Q4. In the last week, have you experienced any of these other symptoms? Please do NOT include symptoms you experience on a regular basis due to a health condition you already know about. Please tick all that apply:

<1> Loss of sense of smell

<2> Loss of sense of taste

<3> Decrease in appetite (skipping meals)

<4> Diarrhoea

<5> Nauseas and/or Vomiting

<6> Abdominal pain/tummy ache

<7> Chills (feeling too cold)

<8> Difficulty sleeping

For peer review only

<9> *Felt more tired than normal*

<10> *Severe Fatigue*

<11> *Sneezing*

<12> *Chest pain / tightness*

<13> *Tightness in chest*

<14> *Sore throat*

<15> *Hoarse voice*

<16> *Runny nose*

<17> *Blocked nose*

<18> *Sore eyes*

<19> *Itchy eyes*

<20> *Headache*

<21> *Joint pain / aches*

<22> *Dizziness*

<23> *Muscle pain/aches*

<99> *None of these*

If you answered, 'None of these', please skip Q5 and go to Q6.

Q5. Thinking about the 14 days before your symptoms started, had you been in physical contact (within 2 metres / 6 feet) with someone who has a confirmed diagnosis of coronavirus (Covid-19), or someone with the following symptoms: dry cough, fever, loss of sense of smell, loss of sense of taste, shortness of breath or difficulty breathing.

<1> *Yes, and it was an individual within my household*

<2> *Yes, and it was an individual from outside my household*

<3> *No, not that I am aware of*

QX Since COVID-19 emerged in January, but before the official lockdown started on March 23rd 2020, which, if any of the following, have you experienced? Please do NOT include symptoms you experience on a regular basis due to a health condition you already know about. Please tick all that apply.

<1> *New, continuous cough (coughing a lot for more than an hour, or have had 3 coughing episodes in 24 hours)*

<2> *High temperature (hot to touch on chest or back)*

<3> *Loss of sense of smell*

<4> *Loss of sense of taste*

<5> *Loss of appetite (skipping meals)*

<6> *Diarrhoea*

<7> *Vomiting*

<8> *Fatigue*

<9> *Sneezing*

<10> *Chest pain / tightness*

For peer review only

<11> Sore throat

<12> Runny nose

<13> Itchy eyes

<14> Headache

<15> Joint pain / aches

<16> Muscle or joint pain

<99> None of these

If you answered, 'None of these', go to Q6.

QXa Approximately when did you start experiencing these symptoms?

[DD/MM/YYYY]

QXb Approximately how long did these symptoms last?

[Days:]

QXX Thinking about the 14 days before your symptoms started, had you been in physical contact (within 2 metres / 6 feet) with someone who has a confirmed diagnosis of coronavirus (Covid-19), or someone with the following symptoms: dry cough, fever, loss of sense of smell, loss of sense of taste, shortness of breath or difficulty breathing.

<1> Yes, and it was an individual within my household

<2> Yes, and it was an individual from outside my household

<3> No, not that I am aware of

Q6 Now, thinking about the period prior to last week, but after the official lockdown started on 23rd March 2020 , which, if any of the following, have you experienced? Please do NOT include symptoms you experience on a regular basis due to a health condition you already know about. Please tick all that apply.

<1> Fever (feeling too hot)

<2> New persistent cough

<3> Shortness of breath affecting normal activities

<4> Loss of sense of smell

<5> Loss of sense of taste

<6> Decrease in appetite (skipping meals)

<7> Diarrhoea

<8> Nauseas and/or vomiting

<9> Abdominal pain/tummy ache

<10> Chills (feeling too cold)

<11> Difficulty sleeping

<12> Felt more tired than normal

<13> Severe fatigue

For peer review only

<14> Sneezing

<15> Chest pain

<16> Tightness in chest

<17> Sore throat

<18> Hoarse throat

<19> Runny nose

<20> Blocked nose

<21> Sore eyes

<22> Itchy eyes

<23> Headache

<24> Dizziness

<25> Joint pain / aches

<26> Muscle pain/aches

If you answered, 'None of these', go to Q8.

Q6a Approximately when did you start experiencing these symptoms?

[DD/MM/YYYY]

Q6b Approximately how long did these symptoms last?

[Days:]

Q7 Thinking about the 14 days before your symptoms started, had you been in physical contact (within 2 metres / 6 feet) with someone who has a confirmed diagnosis of coronavirus (Covid-19), or someone with the following symptoms: dry cough, fever, loss of sense of smell, loss of sense of taste, shortness of breath or difficulty breathing.

<1> Yes, and it was an individual within my household

<2> Yes, and it was an individual from outside my household

<3> No, not that I am aware of

Q8 Have you or anyone in your house been tested for coronavirus? Please tick all that apply

<1> No testing

<2> I have not been tested -- BUT I think I have already had coronavirus and recovered

<3> I was tested - positive result

<4> I was tested - awaiting result

<5> I was tested - negative result

<6> Household member tested - positive result

<7> Household member tested - awaiting result

For peer review only

<8> Household member tested - negative result

Q9 In the last week, has anyone in your household had a new cough or fever?

Not applicable

<1> No

<2> Yes

Q10 Have you had any healthcare contact since the lockdown started? Please tick all that apply

<1> No

<2> Yes - remote appointment with my GP (phone/video)

<3> Yes - I attended my GP practice for an appointment

<4> Yes - remote appointment with hospital (phone/video)

<5> Yes - I attended hospital for an appointment

<6> Yes - attended Accident and Emergency

<7> Yes -- I was admitted to hospital (not because of coronavirus)

<8> Yes -- I was admitted to hospital with symptoms of coronavirus

<9> Yes – One or more remote calls to 111- home visit by ambulance

Q11. In the last week, have you been taking any medication for new symptoms?

<1> No

<2> Yes

<3> If yes, what medication?

Underlying conditions

For the following question, please remember that your answers are always treated confidentially and are never analysed individually. We have provided you with a "Prefer not to say" option if you would rather not share your experiences.

Q12 Which, if any, of the following chronic health conditions have you been diagnosed with? (Please select all that apply. If you do not currently have a chronic health condition, please select the 'None of these' option)

<1> Arthritis

<2> Asthma

For peer review only

<3> My doctor has told me I have severe asthma

<4> I am having cancer treatment

<5> Blood or bone marrow cancer, such as leukaemia

<6> Cystic fibrosis

<7> Chronic obstructive pulmonary disease (COPD)

<8> Diabetes

<9> Epilepsy

<10> Heart disease

<11> High blood pressure

<12> High cholesterol

<13> HIV/ AIDS

<14> Mental health condition

<15> Multiple Sclerosis

<16> I have had an organ transplant

<17> I have a condition that makes me much more likely to get infections

<18> I am taking medicine that weakens my immune system

<19> Dementia, Parkinson's or other neurological disease

<98> Prefer not to say

<99> None of these

Contacts

[Q13 What is your date of birth:

Date …..]

Q14 What is your sex:

<1> Female

<2> Male

<3> Prefer not to say

Q15 What ethnic group best describes you? Please select one option only.

<1>

English / Welsh / Scottish / Northern Irish / British

<11>

Bangladeshi

For peer review only

<2>

Irish

<12>

Chinese

<3>

Gypsy or Irish Traveller

<13>

Any other Asian background

<4>

Any other White background

<14>

African

<5>

White and Black Caribbean

<15>

Caribbean

<6>

White and Black African

<16>

Any other Black / African / Caribbean background

<7>

White and Asian

<17>

Arab

<8>

Any other Mixed / Multiple ethnic background

<18 fixed>

Any other ethnic group

<9>

Indian

<19 fixed>

Prefer not to say

For peer review only

<10>

Pakistani

Q16 Who else is CURRENTLY living in your household? Please tick all that apply

<1> I live by myself

<2> I live with my partner

<3> I live with my child/children aged under 18

<4> I live with my child/children aged over 18

<5> I live with family members other than partner / children

<6> I live with housemates

16a Where are you living during lockdown?

<1> My usual home

<2> not my usual home – keeping away from household members who are at high risk coronavirus

<3> Not my usual home – other reason

Q17 How many people, including yourself, are there in your household? Please include both adults and children. If you live alone, enter 1

Number

Q17a

For each household member ask age (in years) and sex

The following questions will ask you to report on how many people you have come into contact with both inside and outside of your household.

A contact is defined as either:

- *Direct skin-to-skin physical contact (e.g. kiss/embrace/handshake)*
- *Face-to-face conversation with another person which lasts over 3 mins, within 2m distance*
- *Being within 2m distance from another individual for over 5 mins*

Note: if you contacted the same person in different times through the day, they should be counted once.

Q18 How many different people did you have contact with, both inside your household and while outside (after having left your household) in the past 7 days?

For peer review only

Enter 0 if you had no contacts in the last 7 days

1. (enter number)

2. Don't know

Q19a Among the contacts you had, just from yesterday, both inside your household and while outside (after having left your household), how many belonged to the following age groups?

No contacts yesterday

0 to <10 years old (enter number)

10 to <20 years old (enter number)

20 to <30 years old (enter number)

30 to <40 years old (enter number)

40 to <50 years old (enter number)

50 to <60 years old (enter number)

60 to <70 years old (enter number)

70 to <80 years old (enter number)

80 to <90 years old (enter number)

90+ years old (enter number)

12. Don't know

IF Q18 is NOT=0

Q19b How many different people did you come in contact with in the past 7 days outside of your household?

Enter 0 if you had no contacts in the last 7 days outside of your household

1. (enter number)

For peer review only

2. Don't know

IF Q19b is NOT=0

Q19c Among the contacts you had, just from yesterday, outside your household, how many belonged to the following age groups?

No contacts yesterday

0 to <10 years old (enter number)

10 to <20 years old (enter number)

20 to <30 years old (enter number)

30 to <40 years old (enter number)

40 to <50 years old (enter number)

50 to <60 years old (enter number)

60 to <70 years old (enter number)

70 to <80 years old (enter number)

80 to <90 years old (enter number)

90+ years old (enter number)

Don't know

IF Q19b is NOT=0

Q19c Among the contacts that you have had in the past 7 days outside your household, how many contacts occurred at work?

Enter 0 if you had no contacts in the last 7 days outside of your household that occurred at work

For peer review only

1. (enter number)

2. Don't know

For the following questions please answer according to the following terms;

Self-isolation – refers to those who are symptomatic and self-isolating for 7 days from when symptoms started

Shielding – those in specific vulnerable groups staying at home for 12 weeks. These groups would include those with underlying chronic health conditions: cancers, respiratory disease, on immunosuppressants, those at increased risk of infection or pregnant women with heart disease and/or those advised by the NHS of their extremely vulnerable status'.

Household quarantine – 14-day quarantine period for all members of a household from the first day of symptom onset in first case in that household

Social distancing and isolation

Q20 Are you currently in self-isolation?

<1> Yes

<2> No

If yes, for how long:…days

Q21 Are you currently shielding as per government guidelines for vulnerable groups?

<1> Yes

<2> No

Q22 Have you moved residence recently due to the pandemic? Y/N

Q23. Are you single, married, living with a partner, divorced, widowed?

Q24. Are you

<1> Continuing to work in your usual job; at home

<2> Continuing to work in your usual job and leave home for your job <3> volunteering in response to the COVID pandemic

<4> a key worker

<5> unemployed

<6> retired

<7> furloughed (put on leave, still getting paid)

<8> had to close your business due to COVID-19

<9> lost my job due to the lockdown

<10> a student

<99> None of the above

Q25. How often are you now contacting friends/family members remotely (Skype/Zoom/Mobile/landline phone etc)?

Several times per day, once a day, 2-3 x per week, 4-6 x per week, once a week, less than once a week?

For peer review only

Q26 Overall, how are your relationships with other members of your household?

Not applicable

1 = worst

2

3

4

5

6

7

8

9

10 = best

Q27 If you are leaving your home, what activity is this for? Please tick all that apply

<1> I am not leaving my home

<2> Commute to work

<3> Essential shopping

<4> Exercise

<5> Other

Q28 Have you or anyone in your household received a letter or message informing you that you are in the population at 'high risk' from coronavirus? Please tick all that apply.

<1> No - Neither myself or anyone in my household is at 'high risk'

<2> No - but I think I should have

<3> No - but someone in my household is at 'high risk'

<4> Yes - letter about me

<5> Yes - letter about someone in my household

Health behaviours: dietary, alcohol and smoking

Q29 Do you drink alcohol?

<1> Yes (If yes trigger sub-questions)

Drinking less since lockdown

Drinking the same amount since lockdown

Drinking more since lockdown

How many units do you consume per week:…units

For peer review only

(half pint/ 300ml = approx. 1 unit, 175ml glass wine= approx. 2 units)

<2> No (If no, trigger sub-questions)

I never drink alcohol

I had already stopped drinking alcohol before lockdown

I stopped drinking alcohol when lockdown started

Q30 Do you smoke?

<1> Yes (if yes, trigger sub-questions)

Smoking less since lockdown

Smoking the same amount since lockdown

Smoking more since lockdown

If yes, how many cigarettes or roll-ups do you smoke per day:…

<2> No (if now, trigger sub-questions)

I never smoked

I had already stopped smoking before lockdown

I stopped smoking since the lockdown

Q30a) Has there been a change in your vaping (e-cigarettes) status since the coronavirus lockdown?

<1> I never vaped

<2> I had already stopped vaping before

<3> I stopped vaping since the lockdown

<4> Vaping less

<5> Vaping the same amount

<6> Vaping more

Q31 Since the lockdown, are you managing to keep a healthy diet, for example, eating fresh fruits and vegetables?

<1> No change from usual - already had a healthy diet

<2> No change from usual - my diet isn't very healthy

<3> My diet has become more healthy

<4> My diet was healthy before but has got worse since lockdown

Q32 On average, how many portions (or servings) of fruit and vegetables do you eat per day?.......

For peer review only

- One portion is typically 80g, 3 heaped tablespoons of cooked veg or 1 cereal bowl of mixed salad
- Three heaped tablespoons of beans and other pulse vegetables, such as kidney beans, lentils and chickpeas, count as 1 portion.
- The following starchy vegetables should not be included – potatoes, yams, cassava and plantain

Q32a Have you ever skipped meals due to difficulties accessing food as a result of COVID-19?

Yes /No

If yes:

How many meals per week, on average have you missed?

<1> 1-3 meals per week

<2> 4-6 meals per week

<4> 7-9 meals per week

<5> 10 or more meals per week

Biometric data: height and weight

Q33 Please enter your weight: Kg

Q34 Please enter your height:…cm

Q35 Do you have a recent (from the past week) blood pressure?_____mm/Hg

Current Physical activity: International Physical Activity Questionnaire

We are interested in finding out about the kinds of physical activities that people do as part of their everyday lives. The questions will ask you about the time you spent being physically active in the last 7 days. Please answer each question even if you do not consider yourself to be an active person. Please think about the activities you do at work, as part of your house and garden work, to get from place to place, and in your spare time for recreation, exercise or sport.

Think about all the vigorous activities that you did in the last 7 days. Vigorous physical activities refer to activities that take hard physical effort and make you breathe much harder than normal. Think only about those physical activities that you did for at least 10 minutes at a time.

Q36: During the last 7 days, on how many days did you do vigorous physical activities like heavy lifting, digging, aerobics, or fast bicycling?

_____ days per week

If no vigorous physical activities, skip to question 38

Q37: How much time did you usually spend doing vigorous physical activities on one of those days? If you only exercised in hours or minutes, please input a '0' in the non-applicable field.

_____ hours per day

For peer review only

_____ *minutes per day*

Think about all the moderate activities that you did in the last 7 days. Moderate activities refer to activities that take moderate physical effort and make you breathe somewhat harder than normal. Think only about those physical activities that you did for at least 10 minutes at a time.

Q38: During the last 7 days, on how many days did you do moderate physical activities like carrying light loads or bicycling at a regular pace? Do not include walking.

_____ *days per week*

If no moderate physical activities, skip to question 40

Q39: How much time did you usually spend doing moderate physical activities on one of those days? If you only exercised in hours or minutes, please input a '0' in the non-applicable field.

_____ *hours per day*

_____ *minutes per day*

Think about the time you spent walking in the last 7 days. This includes at work and at home, walking to travel from place to place, and any other walking that you have done solely for recreation, sport, exercise, or leisure.

Q40: During the last 7 days, on how many days did you walk for at least 10 minutes at a time?

_____ *days per week*

No walking, skip to question 42

Q41: How much time did you usually spend walking on one of those days? If you only exercised in hours or minutes, please input a '0' in the non-applicable field.

_____ *hours per day*

_____ *minutes per day*

The last question is about the time you spent sitting on weekdays during the last 7 days. Include time spent at work, at home, while doing course work and during leisure time. This may include time spent sitting at a desk, reading, or sitting or lying down to watch television.

Q42: During the last 7 days, how much time did you spend sitting on a week day?

For peer review only

If you only exercised in hours or minutes, please input a ‘0’ in the non-applicable field.

_____ *hours per day*

_____ *minutes per day*

Previous Physical activity: International Physical Activity Questionnaire

These questions will ask you about the time you spent being physically active in the 7 days prior to implementation of social distancing measures (please use first week of March 2020). Please answer each question even if you do not consider yourself to be an active person. Please think about the activities you do at work, as part of your house and garden work, to get from place to place, and in your spare time for recreation, exercise or sport.

Think about all the vigorous activities that you did in the 7 days prior to social distancing measures. Vigorous physical activities refer to activities that take hard physical effort and make you breathe much harder than normal. Think only about those physical activities that you did for at least 10 minutes at a time.

Q43: During the 7 days prior to social distancing measures (please use first week of March 2020), on how many days did you do vigorous physical activities like heavy lifting, digging, aerobics, or fast bicycling?

_____ *days per week*

If no vigorous physical activities, skip to question 45

Q44: How much time did you usually spend doing vigorous physical activities on one of those days? If you only exercised in hours or minutes, please input a ‘0’ in the non-applicable field.

_____ *hours per day*

_____ *minutes per day*

Think about all the moderate activities that you did in the 7 days prior to social distancing measures. Moderate activities refer to activities that take moderate physical effort and make you breathe somewhat harder than normal. Think only about those physical activities that you did for at least 10 minutes at a time.

Q45: During the 7 days prior to social distancing measures (please use first week of March 2020), on how many days did you do moderate physical activities like carrying light loads or bicycling at a regular pace? Do not include walking.

_____ *days per week*

If no moderate physical activities, skip to question 47

For peer review only

Q46: How much time did you usually spend doing moderate physical activities on one of those days? If you only exercised in hours or minutes, please input a '0' in the non-applicable field.

_____ hours per day

_____ minutes per day

Think about the time you spent walking in the 7 days prior to social distancing measures. This includes at work and at home, walking to travel from place to place, and any other walking that you have done solely for recreation, sport, exercise, or leisure.

Q47: During the 7 days prior to social distancing measures (please use first week of March 2020), on how many days did you walk for at least 10 minutes at a time?

_____ days per week

No walking, skip to question 49

Q48: How much time did you usually spend walking on one of those days? If you only exercised in hours or minutes, please input a '0' in the non-applicable field.

_____ hours per day

_____ minutes per day

The last question is about the time you spent sitting on weekdays during 7 days prior to social distancing measures. Include time spent at work, at home, while doing course work and during leisure time. This may include time spent sitting at a desk, reading, or sitting or lying down to watch television.

Q49: During the 7 days prior to social distancing measures (please use first week of March 2020), how much time did you spend sitting on a week day? If you only exercised in hours or minutes, please input a '0' in the non-applicable field.

_____ hours per day

_____ minutes per day

For peer review only

Frailty Questionnaire:

Q50: Are you fatigued?

<1> Yes

<2> No

Q51: Can you walk up one flight of stairs?

<1> Yes

<2> No

Q52: Can you walk around the block?

<1> Yes

<2> No

Q53: Do you have more than 5 illnesses?

<1> Yes

<2> No

Q54: Have you lost more than 5% of your weight in the past 6 months?

<1> Yes

<2> No

Qx Have you had a fall during the COVID lockdown period?

Yes/No

If yes:

What actions were taken (select multiple where applicable):

a) No follow-up required, I did not hurt myself

b) Pain medication

c) A hospital and/or GP appointment

d) A follow-up X-ray

e) Sling/plaster cast for a fracture

For peer review only

f) Surgery

Mood

a) Tick the box beside the reply that is closest to how you have been feeling in the past week.

Don't take too long over you replies: your immediate response is best.

Tick here

1.

Tick here

8.

I feel tense or 'wound up':

I feel as if I am slowed down:

Most of the time

Nearly all the time

A lot of the time

Very often

From time to time, occasionally

Sometimes

Not at all

For peer review only

Not at all

2.

9.

I still enjoy the things I used to

enjoy:

I get a sort of frightened feeling like 'butterflies' in the stomach:

Definitely as much

Not at all

Not quite so much

Occasionally

Only a little

Quite Often

Hardly at all

Very Often

3.

10.

I get a sort of frightened feeling as if something awful is about to

happen:

I have lost interest in my appearance:

For peer review only

Very definitely and quite badly

Definitely

Yes, but not too badly

I don't take as much care as I should

A little, but it doesn't worry me

I may not take quite as much care

Not at all

I take just as much care as ever

4.

11.

I can laugh and see the funny side of things:

I feel restless as I have to be on the move:

As much as I always could

Very much indeed

Not quite so much now

Quite a lot

Definitely not so much now

Not very much

For peer review only

Not at all

Not at all

5.

12.

Worrying thoughts go through my mind:

I look forward with enjoyment to things:

A great deal of the time

As much as I ever did

A lot of the time

Rather less than I used to

From time to time, but not too often

Definitely less than I used to

Only occasionally

Hardly at all

6.

13.

I feel cheerful:

For peer review only

I get sudden feelings of panic:

Not at all

Very often indeed

Not often

Quite often

Sometimes

Not very often

Most of the time

Not at all

7.

14.

I can sit at ease and feel relaxed:

I can enjoy a good book or radio or TV program:

Definitely

Often

Usually

Sometimes

Not Often

For peer review only

Not often

Not at all

Very seldom

Qx . I experience a general sense of emptiness

- *Not ever*
- *Rarely*
- *Sometimes*
- *Often*

Qy. There are plenty of people I can rely on when I have problems

- *Not ever*
- *Rarely*
- *Sometimes*
- *Often*

Qz. I miss having people around me

- *Not ever*
- *Rarely*
- *Sometimes*
- *Often*

Please check you have answered all the questions above.

b) For each of the 17 mood questions above, please also indicate if you are feeling or experiencing this 1, less than; 2, the same as; or 3, more than before social isolation was implemented.

<1> [1 or 2 or 3]

<2> [1 or 2 or 3]

<3> [1 or 2 or 3]

<4> [1 or 2 or 3]

<5> [1 or 2 or 3]

<6> [1 or 2 or 3]

<7> [1 or 2 or 3]

<8> [1 or 2 or 3]

<9> [1 or 2 or 3]

<10> [1 or 2 or 3]

For peer review only

<11> [1 or 2 or 3]

<12> [1 or 2 or 3]

<13> [1 or 2 or 3]

<14> [1 or 2 or 3]

<15> [1 or 2 or 3]

<16> [1 or 2 or 3]

<17> [1 or 2 or 3]

Q. People may have worries about Covid-19. Have you been worried about any of the following and, if so, how much?

Not at all

Little

Some

Rather much

Very much

Getting Covid-19 infection and/or infecting someone else

That a person close to me could get infected with Covid-19

Being discriminated against or avoided because of Covid-19

Impact of the Covid-19 epidemic on my own

For peer review only

economy and/or loss of my employment

Economic impact of the Covid-19 epidemic on the global economy

The government's and/or health system's lack of ability to handle the Covid-19 pandemic situation, including the shortage of food and other groceries

Imperial College Sleep Quality (ICSQ) Questionnaire

Instructions:

The following questions relate to your usual sleep habits for a period of one month before and during a period of reduced social contact. Your answers should indicate the most accurate reply for the majority of days and nights during these periods.

Please answer all questions.

1. During the period before reduced social contact, what time did you usually go to bed at night: bed-time was …………………………………………………

1b) During the period of reduced social contact, what time have you usually gone to bed at night: bed-time is - …………………………………………………......

2. During the period before reduced social contact, how long (in minutes) did it usually take you to fall asleep each night: number of minutes - …………………

2b) During the period of reduced social contact, how long (in minutes) has it usually taken you to fall asleep each night: number of minutes - ……………………………

3. During the period before reduced social contact, what time did you usually get up in the morning: getting-up time was - ………………………………………

3b) During the period of reduced social contact, what time do you usually get up in the morning: getting-up time is - …………………………………………………………..

4. During the period before reduced social contact, how many hours of actual sleep did you get at night? (This may be different from the number of hours you

For peer review only

spent in bed): hours of sleep per night - …………………………………………

4b) During the period of reduced social contact, how many hours of actual sleep do you get at night? (This may be different from the number of hours you spend in bed): hours of sleep per night - ………………………………………………...

5. During the period before reduced social contact, how often did you have trouble sleeping because you could not get to sleep within 30 minutes:

o Not ever

o Less than once a week

o Once or twice a week

o Three or more times a week

5b) During the period of reduced social contact, how often have you had trouble sleeping because you could not get to sleep within 30 minutes:

o Not ever

o Less than once a week

o Once or twice a week

o Three or more times a week

6. During the period before reduced social contact, did you experience poor sleep (restless and unable to sleep):

o Not ever

o Less than once a week

o Once or twice a week

o Three or more times a week

6b) During the period of reduced social contact, have you experienced poor sleep (restless and unable to sleep):

o Not ever

o Less than once a week

o Once or twice a week

o Three or more times a week

7a) During the period before reduced social contact, did you experience loneliness (felt isolated, with no companions):

o Not ever

o Rarely

o Sometimes

o Often

7b) During the period of reduced social contact, have you experienced loneliness (felt isolated, with no companions):

o Not ever

o Rarely

o Sometimes

o Often

7c) During the period of reduced social contact, have you experienced loneliness: 1,

For peer review only

less than; 2, the same as; or 3, more than before social isolation was implemented

Select: [1 or 2 or 3]

Functional Activities Questionnaire

For each of the tasks below please rate your ability to carry out the task/activity independently on the following scale:

1. I had no difficulty

2. I had some difficulty, but I completed the task/activity myself.

3. I need some assistance to complete the task/activity:

a) I did not need assistance prior to COVID-19 lockdown but need assistance now to maintain social isolation/distancing

b) I could do the task/activity before the COVID-19 lockdown, but now would need assistance even if it were not to maintain social distancing

c) I required assistance since before the COVID-19 lockdown

4. I needed others to do this for me,

a) I could do the task/activity myself or with assistance prior to COVID-19 lockdown but need others to do it for me to maintain social isolation/distancing

b) I could do the task/activity myself or with assistance before the COVID-19 lockdown, but now would need others to do it for me even if it were not to maintain social distancing

c) I required others to do it for me since before the COVID-19 lockdown

5. I am unsure if I require assistance (e.g., never did the task/activity or have not done the task/activity over the past week)

Activities:

1. Writing cheques, paying bills, balancing cheque book, using an ATM cash machine

Response: …………………

2. Assembling tax records, business affairs, or papers

Response: …………………

3. Shopping alone for household necessities, medicines or groceries

Response: …………………

4. Playing a game of skill, working on a hobby

Response: …………………

5. Heating water, making a cup of coffee, turning off stove after use

Response: …………………

For peer review only

6. Preparing a balanced meal

Response: …………………

7. Keeping track of current events

Response: …………………

8. Paying attention to, understanding, discussing TV, video, book, magazine

Response: …………………

9. Remembering appointments, family occasions, public holidays, to take medications

Response: …………………

10. Travelling out of my neighbourhood by taxi, car, bus or train and making travel arrangements.

Response: …………………

THANK YOU FOR COMPLETING THIS QUESTIONNAIRE. YOUR RESPONSES HAVE BEEN SAVED AND SENT TO THE STUDY TEAM.

NHS health advice and information regarding the novel coronavirus can be found here: https://www.nhs.uk/conditions/coronavirus-covid-19/

For Advice on Mental health we suggest using these links:

1. The NHS Every Mind Matters website has information on how to look after your mental wellbeing while in isolation: https://www.nhs.uk/oneyou/every-mind-matters/

2. The charity Mental Health UK have advice on managing mental health during the coronavirus outbreak: https://mentalhealth-uk.org/help-and-information/covid-19-and-your-mental-health/

3. The NHS recommends a range of mobile apps to help with mental wellbeing, many of which are free to download: https://www.nhs.uk/apps-library/category/mental-health/

4. If you need someone to talk to about your mental health, the charity Samaritans have a helpline available 24 hours a day, 7 days a week:

a. Call: 116 123

b. or visit: https://www.samaritans.org/how-we-can-help/contact-samaritan/

For Advice on Physical activity we suggest using these links:

1. The NHS Live Well website has a range of free advice and programmes from light activity to more strenuous exercises for those aged under 65: https://www.nhs.uk/live-well/exercise/

2. The NHS Live Well website has a range of free advice and programmes from light activity to more strenuous exercises for those aged 65 or older: https://www.nhs.uk/live-well/exercise/physical-activity-guidelines-older-adults/

3. Tips, advice and guidance from Sport England on how to keep or get active in and around your home: https://www.sportengland.org/stayinworkout

For peer review only

4. Stay Active at Home: a simple set of exercises designed for older people to stay active at home: https://www.csp.org.uk/public-patient/keeping-active-and-healthy/staying-healthy-you-age/staying-strong-you-age/strength

For Advice on Sleep we suggest using these links:

1. The NHS ten top tips to improve sleep: https://www.nhs.uk/live-well/sleep-and-tiredness/10-tips-to-beat-insomnia/

2. The NHS recommends a range of mobile apps to help with sleep: https://www.nhs.uk/apps-library/category/sleep/

Supplementary Table 1: CCRR survey

For peer review only

Supplementary file 2

Supplementary methods

<u>Metabolic Equivalent of Task (MET) calculation</u>

Briefly, 1 MET equates to an individual’s resting energy expenditure. According to the IPAQ scoring protocol, 3.3 METS is considered equivalent to walking, and moderate and vigorous activity to be 4 and 8 METS, respectively. To calculate the continuous variable of total MET minutes a week, the self-reported duration (minutes) and frequency (days) of each of these PA categories is multiplied by the by the specified metric.

Supplementary figures and tables

Figure 1: Box-plot of distribution of MET minutes per week before and during lockdown for 6,219 participants with completed IPAQ data

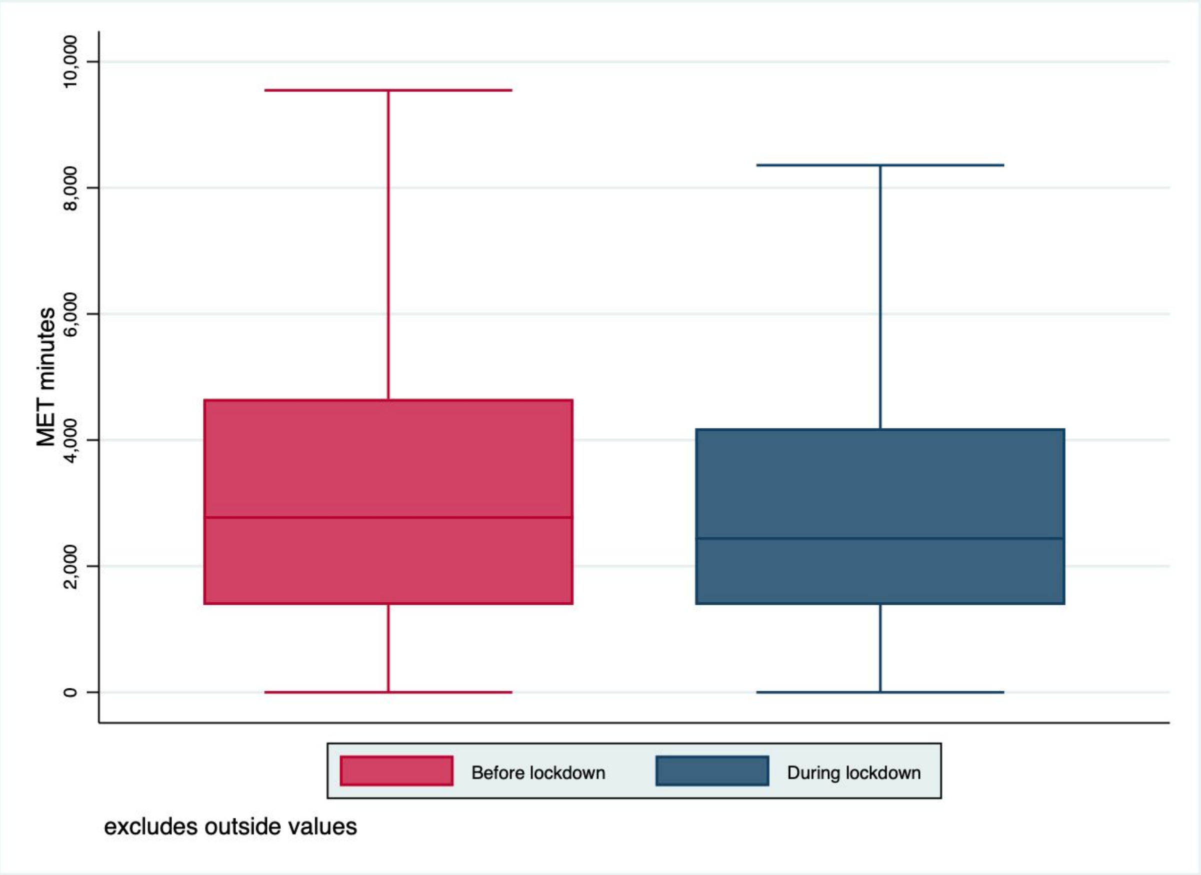

Figure 2: Box-plot of distribution of MET minutes per week after introduction of lockdown by month of survey completion for 6,219 participants with completed IPAQ data

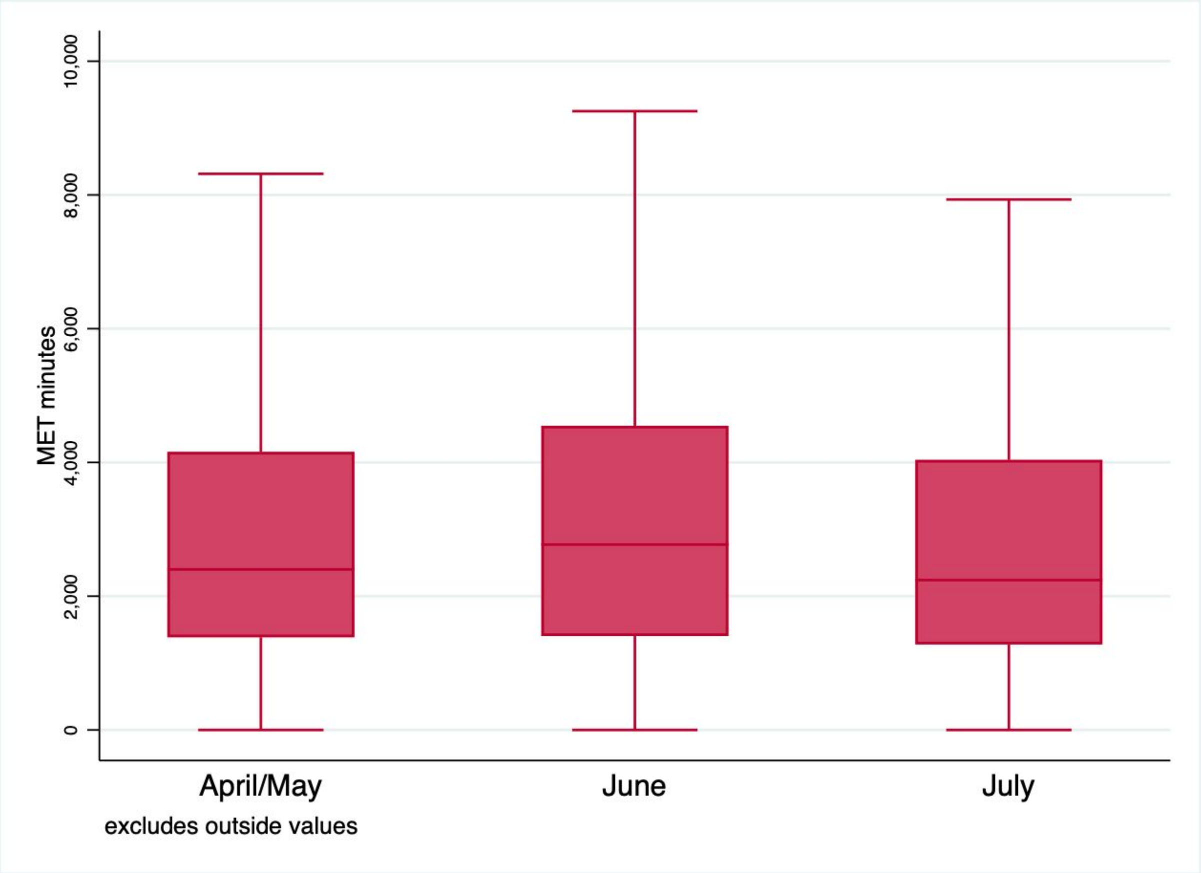

Table 1: Mean MET minutes after introduction of lockdown measures by month of survey completion

Month	Total	Percent	Mean MET minutes	p value¶
April/May*	4975	80.0%	3139	
June	994	16.0%	3470	0.0007
July	250	4.0%	2967	

* April (110) and May (4865) combined due to small numbers completed in April
¶ p-value from linear regression models of MET minutes as dependent variable, against survey completion month as explanatory variable.

For peer review only - http://bmjopen.bmj.com/site/about/guidelines.xhtml

Figure 3: Box-plot of distribution of MET minutes per week before introduction of lockdown by month of survey completion for 6,219 participants with completed IPAQ data

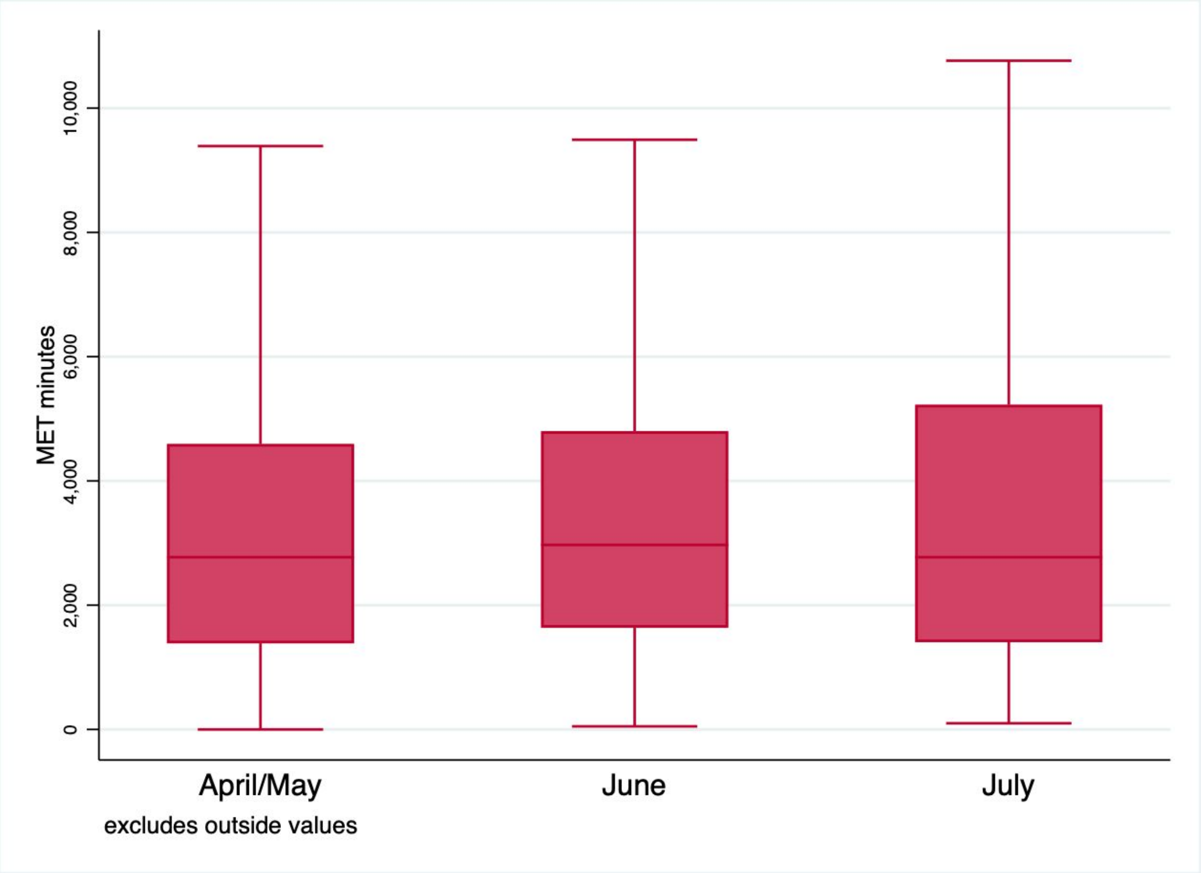

Linear regression models of MET minutes as dependent variable, against survey completion month as explanatory variable showed no significant association (p=0.1112).

Figure 4: Causal diagram representing factors impacting on change in physical activity after lockdown

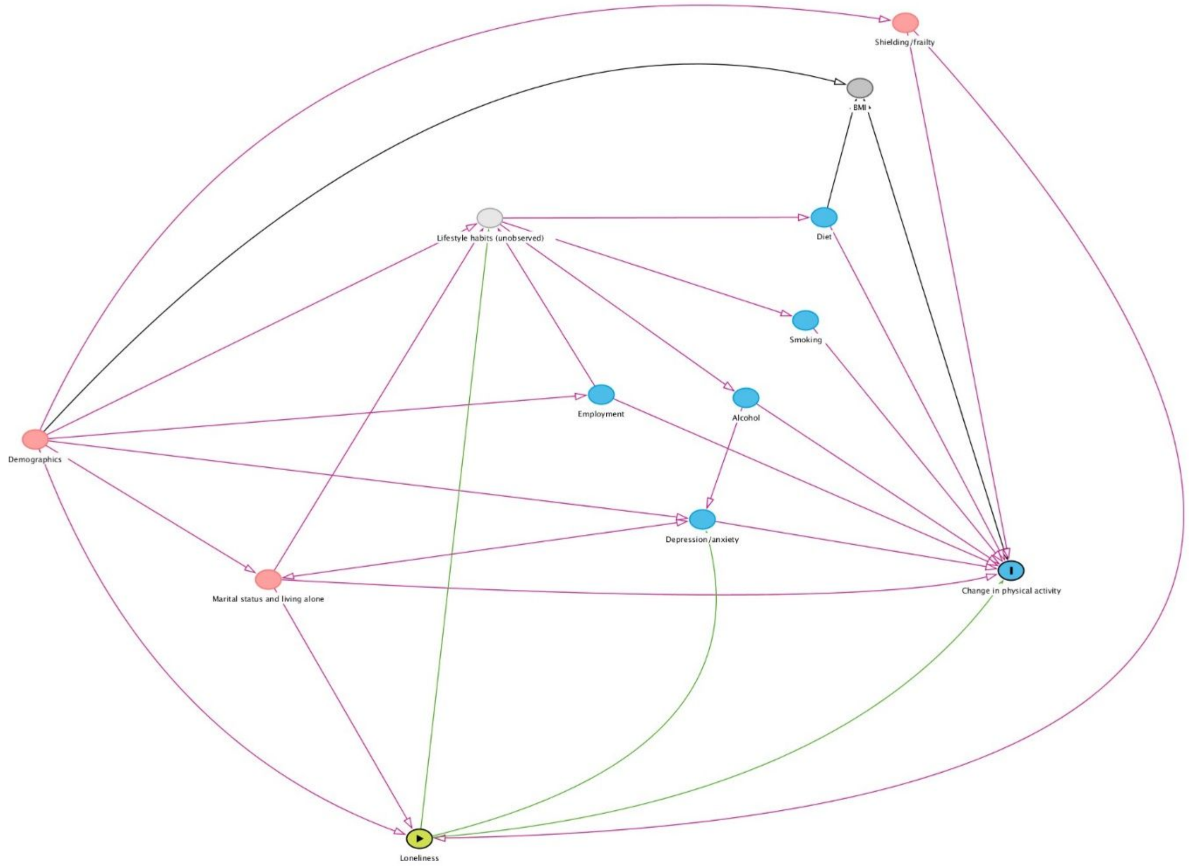

view only

Table 2: Unadjusted associations in physical activity (MET minutes per week) after introduction of lockdown measures and change from before lockdown, from linear regression models. Note: negative values for change in activity indicate reduction after lockdown

Predictor	Physical activity after lockdown (MET minutes/week)				Change in physical activity from before lockdown (MET minutes/week)			
	Mean	95% confidence interval		p value	Mean	95% confidence interval		p value
		Lower	Upper			Lower	Upper	
**Mean (whole cohort)**	3186	3120	3253	-	-333	-396	-271	-
**Age (years)**								
50-64	3341	3191	3491	<0.001	-196	-338	-55	0.184
65-74	3201	3112	3290		-362	-446	-278	
75-84	3092	2952	3232		-365	-497	-233	
85+	2326	1863	2790		-503	-940	-66	
**Sex**								
Female	3227	3138	3317	0.180	-450	-533	-366	<0.001
Male	3136	3036	3235		-189	-282	-95	
**Ethnicity**								
White	3196	3127	3265	0.425	-330	-394	-265	0.641
Mixed/multiple ethnic groups	3346	2819	3873		-392	-887	102	
Asian/Asian British	2929	2530	3327		-326	-700	48	
Black/African/Caribbean/Black British	3351	2551	4151		4	-746	754	
Other ethnic group	2754	2099	3410		-740	-1355	-125	
**Body Mass Index category**								
Underweight	3815	3137	4493	<0.001	-21	-649	607	0.055
Healthy weight	3569	3439	3700		-126	-247	-5	
Overweight	3130	2959	3300		-363	-521	-204	
Obese	2590	2309	2870		-400	-659	-140	

**Employment status**								
Employed	3093	2950	3236		-217	-351	-84	
Furloughed	3496	3122	3870	0.118	-488	-838	-139	0.101
Unemployed	3463	3031	3894		-672	-1076	-268	
Retired	3191	3111	3271		-334	-409	-259	
**Marital status**								
Divorced/single/widowed	3026	2908	3143	0.001	-540	-650	-430	<0.001
Living with a partner/married	3262	3181	3342		-236	-312	-161	
**Household**								
Not living alone	3262	3185	3340	<0.001	-240	-313	-168	<0.001
Living alone	2983	2855	3110		-582	-702	-463	
**Loneliness**								
Not ever	3284	3188	3380		-216	-306	-126	
Rarely	3087	2951	3224	0.024	-360	-488	-232	<0.001
Sometimes	3155	3010	3300		-481	-617	-345	
Often	2938	2666	3210		-762	-1018	-507	
**Shielding**								
Not shielding	3273	3196	3350	<0.001	-243	-315	-171	<0.001
Shielding	2942	2812	3072		-588	-710	-466	
**Frailty**								
Robust	3257	3183	3330		-335	-404	-265	
Pre-frail	2903	2746	3059	<0.001	-328	-475	-180	0.389
Frail	1952	1055	2849		-925	-1768	-82	
**Alcohol drinker**								
No	3044	2884	3203	0.054	-485	-634	-335	0.029
Yes	3217	3143	3290		-301	-370	-233	
**Smoker**				0.010				0.046
No	3202	3135	3270		-322	-385	-259	
Yes	2696	2318	3074		-689	-1043	-334	

For peer review only

**Diet**								
No change from usual - already had a healthy diet	3257	3183	3331	<0.001	-333	-402	-263	<0.001
My diet has become more healthy	3314	3119	3509		-55	-238	129	
My diet was healthy before but has got worse since lockdown	2523	2227	2818		-890	-1168	-612	
No change from usual - my diet isn't very healthy	2009	1641	2377		-479	-825	-133	
**HADS (depression score)**								
Normal (0-7)	3195	3119	3270	<0.001	-293	-365	-222	<0.001
Borderline (8-10)	2787	2495	3079		-676	-953	-399	
Abnormal (11-21)	2450	1971	2929		-1450	-1904	-997	
**HADS (anxiety score)**								
Normal (0-7)	3123	3044	3201	0.150	-312	-386	-237	0.004
Borderline (8-10)	3343	3109	3577		-348	-570	-125	
Abnormal (11-21)	3288	2971	3605		-836	-1137	-535	

*HADS – Hospital Anxiety and Depression Score

For peer review only

Table 3: Results of multivariable linear regression models of physical activity after lockdown, adjusted for age, sex, ethnicity, month of survey completion and baseline physical activity. Data presented as mean MET minutes/week +/- 95% confidence interval compared to the reference group, with negative values indicating lower physical activity than the reference.

Predictor	Physical activity after lockdown (MET minutes/week)	95% confidence interval		p value	Number of observations
		Lower	Upper		
**Age (years)**					
50-64 (reference)	-	-	-	<0.001	6155
65-74	-154	-296	-12		
75-84	-213	-380	-46		
85+	-640	-1034	-246		
**Sex**					
Female (reference)	-	-	-	0.053	6155
Male	108	-1	216		
**Ethnicity**					
White (reference)	-	-	-	0.517	6155
Mixed/multiple ethnic groups	-14	-442	415		
Asian/Asian British	-136	-463	191		
Black/African/Caribbean/Black British	248	-398	894		
Other ethnic group	-435	-969	100		
**Body Mass Index category**					
Underweight	153	-411	717	0.030	2987
Healthy weight (reference)	-	-	-		
Overweight	-341	-518	-165		
Obese	-578	-832	-324		
**Employment status**					
Employed (reference)	-	-	-	0.905	5958
Furloughed	47	-278	372		
Unemployed	-110	-480	259		

For peer review only

Retired	99	-48	246		
**Marital status**					
Divorced/single/widowed (reference)	-	-	-	<0.001	6155
Living with a partner/married	240	120	360		
**Household**					
Not living alone (reference)	-	-	-	<0.001	6155
Living alone	-277	-402	-152		
**Loneliness**					
Not ever (reference)	-	-	-		
Rarely	-161	-297	-25	<0.001	6077
Sometimes	-186	-329	-42		
Often	-452	-688	-217		
**Shielding**					
Not shielding (reference)	-	-	-	<0.001	6155
Shielding	-290	-417	-163		
**Frailty**					
Robust (reference)	-	-	-	0.005	6142
Pre-frail	-160	-301	-19		
Frail	-926	-1663	-189		
**Alcohol drinker**					
No (reference)	-	-	-	0.049	6155
Yes	145	1	289		
**Smoker**					
No (reference)				0.005	6155
Yes	-451	-762	-140		
**Diet**					
No change from usual - already had a healthy diet (reference)	-	-	-	<0.001	6155
My diet has become more healthy	156	-13	326		
My diet was healthy before but has got worse since lockdown	-662	-910	-414		

For peer review only

No change from usual - my diet isn't very healthy	-667	-975	-359		
**HADS (depression score)**					
Normal (0-7) (reference)	-	-	-	<0.001	5038
Borderline (8-10)	-408	-654	-163		
Abnormal (11-21)	-1007	-1401	-612		
**HADS (anxiety score)**					
Normal (0-7) (reference)	-	-	-	0.478	5038
Borderline (8-10)	94	-109	296		
Abnormal (11-21)	-220	-486	47		

HADS – Hospital Anxiety and Depression Score

For peer review only

Table 4: Multivariable linear regression model for physical activity after lockdown with loneliness, adjusted for age, sex, ethnicity, month of survey completion, baseline physical activity, living alone, marital status, shielding and frailty

Predictor	Physical activity after lockdown (MET minutes/week)	95% confidence interval		p value	Number of observations
		Lower	Upper		
**Loneliness**					
Not ever (reference)	-	-	-		
Rarely	-127	-265	11	0.007	6077
Sometimes	-107	-256	42		
Often	-306	-552	-60		

Adjusted: age, sex, ethnicity, month of survey completion, baseline physical activity, living alone, marital status, shielding, frailty

For peer review only

STROBE 2007 (v4) checklist of items to be included in reports of observational studies in epidemiology*

Checklist for cohort, case-control, and cross-sectional studies (combined)

Section/Topic	Item #	Recommendation	Reported on page #
**Title and abstract**	1	(*a*) Indicate the study's design with a commonly used term in the title or the abstract	1
		(*b*) Provide in the abstract an informative and balanced summary of what was done and what was found	4
**Introduction**			
Background/rationale	2	Explain the scientific background and rationale for the investigation being reported	5
Objectives	3	State specific objectives, including any pre-specified hypotheses	5
**Methods**			
Study design	4	Present key elements of study design early in the paper	5
Setting	5	Describe the setting, locations, and relevant dates, including periods of recruitment, exposure, follow-up, and data collection	5-6
Participants	6	(*a*) *Cohort study*—Give the eligibility criteria, and the sources and methods of selection of participants. Describe methods of follow-up<br>*Case-control study*—Give the eligibility criteria, and the sources and methods of case ascertainment and control selection. Give the rationale for the choice of cases and controls<br>*Cross-sectional study*—Give the eligibility criteria, and the sources and methods of selection of participants	5-6
		(*b*) *Cohort study*—For matched studies, give matching criteria and number of exposed and unexposed<br>*Case-control study*—For matched studies, give matching criteria and the number of controls per case	
Variables	7	Clearly define all outcomes, exposures, predictors, potential confounders, and effect modifiers. Give diagnostic criteria, if applicable	6
Data sources/ measurement	8*	For each variable of interest, give sources of data and details of methods of assessment (measurement). Describe comparability of assessment methods if there is more than one group	6-7
Bias	9	Describe any efforts to address potential sources of bias	6-7
Study size	10	Explain how the study size was arrived at	6
Quantitative variables	11	Explain how quantitative variables were handled in the analyses. If applicable, describe which groupings were chosen and why	6-7
Statistical methods	12	(*a*) Describe all statistical methods, including those used to control for confounding	6-7
		(*b*) Describe any methods used to examine subgroups and interactions	6-7
		(*c*) Explain how missing data were addressed	6-7
		(*d*) *Cohort study*—If applicable, explain how loss to follow-up was addressed<br>*Case-control study*—If applicable, explain how matching of cases and controls was addressed	NA

For peer review only

For peer review only - http://bmjopen.bmj.com/site/about/guidelines.xhtml

		Cross-sectional study—If applicable, describe analytical methods taking account of sampling strategy	
		(*e*) Describe any sensitivity analyses	NA
**Results**			
Participants	13*	(a) Report numbers of individuals at each stage of study—eg numbers potentially eligible, examined for eligibility, confirmed eligible, included in the study, completing follow-up, and analysed	6
		(b) Give reasons for non-participation at each stage	NA
		(c) Consider use of a flow diagram	NA
Descriptive data	14*	(a) Give characteristics of study participants (eg demographic, clinical, social) and information on exposures and potential confounders	7
		(b) Indicate number of participants with missing data for each variable of interest	Table 1
		(c) *Cohort study*—Summarise follow-up time (eg, average and total amount)	NA: baseline analysis
Outcome data	15*	*Cohort study*—Report numbers of outcome events or summary measures over time	NA: baseline analysis
		Case-control study—Report numbers in each exposure category, or summary measures of exposure	
		Cross-sectional study—Report numbers of outcome events or summary measures	
Main results	16	(*a*) Give unadjusted estimates and, if applicable, confounder-adjusted estimates and their precision (eg, 95% confidence interval). Make clear which confounders were adjusted for and why they were included	7-9
		(*b*) Report category boundaries when continuous variables were categorized	7-9
		(*c*) If relevant, consider translating estimates of relative risk into absolute risk for a meaningful time period	NA
Other analyses	17	Report other analyses done—eg analyses of subgroups and interactions, and sensitivity analyses	7-9
**Discussion**			
Key results	18	Summarise key results with reference to study objectives	10
Limitations	19	Discuss limitations of the study, taking into account sources of potential bias or imprecision. Discuss both direction and magnitude of any potential bias	12-13
Interpretation	20	Give a cautious overall interpretation of results considering objectives, limitations, multiplicity of analyses, results from similar studies, and other relevant evidence	10-12
Generalisability	21	Discuss the generalisability (external validity) of the study results	10-12
**Other information**			
Funding	22	Give the source of funding and the role of the funders for the present study and, if applicable, for the original study on which the present article is based	2

*Give information separately for cases and controls in case-control studies and, if applicable, for exposed and unexposed groups in cohort and cross-sectional studies.

Note: An Explanation and Elaboration article discusses each checklist item and gives methodological background and published examples of transparent reporting. The STROBE checklist is best used in conjunction with this article (freely available on the Web sites of PLoS Medicine at http://www.plosmedicine.org/, Annals of Internal Medicine at http://www.annals.org/, and Epidemiology at http://www.epidem.com/). Information on the STROBE Initiative is available at www.strobe-statement.org.

For peer review only

BMJ Open

The impact of social restrictions during the COVID-19 pandemic on the physical activity levels of adults aged 50-92 years: a baseline survey of the CHARIOT COVID-19 Rapid Response prospective cohort study

Journal:	*BMJ Open*
Manuscript ID	bmjopen-2021-050680.R1
Article Type:	Original research
Date Submitted by the Author:	14-Jul-2021

Complete List of Authors:	Salman, David; Imperial College London Department of Primary Care and Public Health; Imperial College London, MSk lab, Department of Surgery and Cancer, Faculty of Medicine<br>Beaney, Thomas; Imperial College London, Department of Primary Care and Public Health<br>Robb, Catherine; Imperial College London, Ageing Epidemiology Research Unit (AGE), School of Public Health, Faculty of Medicine<br>de-Jaegar Loots, Celeste; Imperial College London, Ageing Epidemiology Research Unit (AGE), School of Public Health, Faculty of Medicine<br>Giannakopoulou, Parthenia; Imperial College London, Ageing Epidemiology Research Unit (AGE), School of Public Health, Faculty of Medicine<br>Udeh-Momoh, Chinedu ; Imperial College London, Ageing Epidemiology Research Unit (AGE), School of Public Health, Faculty of Medicine<br>Ahmadi-Abhari, Sara ; Imperial College London, Ageing Epidemiology Research Unit (AGE), School of Public Health, Faculty of Medicine<br>Majeed, Azeem; Imperial College London, Department of Primary Care and Public Health; Imperial College Healthcare NHS Trust, Public Health Directorate<br>Middleton, Lefkos; Imperial College London, Ageing Epidemiology Research Unit (AGE), School of Public Health, Faculty of Medicine; Imperial College Healthcare NHS Trust, Public Health Directorate<br>McGregor, Alison; Imperial College London, MSk lab, Department of Surgery and Cancer, Faculty of Medicine

**Primary Subject Heading**:	Sports and exercise medicine
Secondary Subject Heading:	General practice / Family practice, Geriatric medicine, Public health
Keywords:	COVID-19, PREVENTIVE MEDICINE, PUBLIC HEALTH, SPORTS MEDICINE, GERIATRIC MEDICINE

For peer review only - http://bmjopen.bmj.com/site/about/guidelines.xhtml

I, the Submitting Author has the right to grant and does grant on behalf of all authors of the Work (as defined in the below author licence), an exclusive licence and/or a non-exclusive licence for contributions from authors who are: i) UK Crown employees; ii) where BMJ has agreed a CC-BY licence shall apply, and/or iii) in accordance with the terms applicable for US Federal Government officers or employees acting as part of their official duties; on a worldwide, perpetual, irrevocable, royalty-free basis to BMJ Publishing Group Ltd ("BMJ") its licensees and where the relevant Journal is co-owned by BMJ to the co-owners of the Journal, to publish the Work in this journal and any other BMJ products and to exploit all rights, as set out in our licence.

The Submitting Author accepts and understands that any supply made under these terms is made by BMJ to the Submitting Author unless you are acting as an employee on behalf of your employer or a postgraduate student of an affiliated institution which is paying any applicable article publishing charge ("APC") for Open Access articles. Where the Submitting Author wishes to make the Work available on an Open Access basis (and intends to pay the relevant APC), the terms of reuse of such Open Access shall be governed by a Creative Commons licence – details of these licences and which Creative Commons licence will apply to this Work are set out in our licence referred to above.

Other than as permitted in any relevant BMJ Author's Self Archiving Policies, I confirm this Work has not been accepted for publication elsewhere, is not being considered for publication elsewhere and does not duplicate material already published. I confirm all authors consent to publication of this Work and authorise the granting of this licence.

For peer review only

The impact of social restrictions during the COVID-19 pandemic on the physical activity levels of adults aged 50-92 years: a baseline survey of the CHARIOT COVID-19 Rapid Response prospective cohort study

David Salman, academic clinical fellow in primary care*[1,2@], **Thomas Beaney,** academic clinical fellow in primary care*[2@], **Catherine E. Robb,** postgraduate research associate*[3], Celeste A. de Jager Loots, research fellow[3], Parthenia Giannakopoulou, data management coordinator[3], Chi Udeh-Momoh, research programme manager[3], Sara Ahmadi-Abhari, lecturer in epidemiology of ageing[3], Azeem Majeed, chair: primary care and public health[2,4], Lefkos T. Middleton, chair: neurology, neuroepidemiology and ageing[3,4], Alison. H. McGregor, professor of musculoskeletal biodynamics[1]

*contributed equally as joint lead authors

Author affiliations:

[1]MSk lab, Faculty of Medicine, Imperial College London, UK, W12 0BZ

[2]Department of Primary Care and Public Health, Imperial College London, UK, W6 8RP

[3]Ageing Epidemiology Research Unit (AGE), School of Public Health, Faculty of Medicine, Imperial College London, UK

[4] Public Health Directorate, Imperial College NHS Healthcare Trust, London, UK

[@]**Correspondence to:**

David Salman

ORCID ID: 0000-0002-1481-8829

MSk lab, 2nd Floor, Sir Michael Uren Hub, 86 Wood Lane, London, W12 0BZ

Email: d.salman11@imperial.ac.uk

Phone: +44 (0) 20 7594 2703

Thomas Beaney

ORCID ID: 0000-0001-9709-7264

Department of Primary Care and Public Health

Charing Cross Campus, The Reynolds Building, St Dunstan's Road, London, W6 8RP

Email: thomas.beaney@imperial.ac.uk

Phone: +44 (0) 207 5943 368

Word count: 4306

Figures:3

Tables:2

For peer review only - http://bmjopen.bmj.com/site/about/guidelines.xhtml

Contributorship and the guarantor

DS, TB and CR conceived the paper, developed the survey materials, carried out the analysis, wrote the paper equally as joint lead authors and are the guarantors. CAdJ, PG, CU-M and SA-A developed the survey materials, managed the cohort and dataset and contributed to the analysis and writing and editing of the paper. AM, LM and AHM developed the survey materials, supervised and managed the survey collection and analysis, and contributed to the writing and editing of the paper. All authors developed the survey, carried out analysis and contributed to the development and editing of the paper.

Transparency declaration

The lead authors confirm that the submitted manuscript is an honest, accurate and transparent account of the study being reported. No important aspects of the study have been omitted.

Ethics approval

This research was approved by the Imperial College Research and Ethics Committee (ICREC) and Joint Research Compliance Office (22/04/2020; 20IC5942). All participants were required to provide informed consent before taking part in the study. Data collected as a part of this study are anonymized and kept strictly confidential in accordance with the UK General Data Protection Regulations (2016).

Data sharing

This is an ongoing study, but anonymised data can be provided upon request for the purposes of further data analysis, and can be requested from the Data Management Co-ordinator, Parthenia Giannakopoulou: parthenia.giannakopoulou13@imperial.ac.uk

Dissemination declaration

Participants in the CHARIOT cohort are informed by regular newsletter of all publications pertaining to the cohort.

Funding statement

Work towards this article was in part supported by the National Institute for Health Research (NIHR) Applied Research Collaboration Northwest London and Imperial Biomedical Research Centre (BRC). DS and TB are supported by NIHR academic clinical fellowships. The views expressed in this publication are those of the authors and not necessarily those of the National Institute for Health Research or the Department of Health and Social Care. Imperial College London is the sponsor for the CCRR study, and has no influence on the direction or content of the work. There was no external financial funding for the study.

For peer review only - http://bmjopen.bmj.com/site/about/guidelines.xhtml

Acknowledgements

We are grateful to Lesley Williamson, Monica Munoz-Troncoso, Snehal Pandya and Emily Pickering (CHARIOT register and facilitator team); Mariam Jiwani, Rachel Veeravalli, Islam Saiful, Danielle Rose, Susie Gold, Rachel Nejade and Shehla Shamsuddin (Imperial College London student volunteers); Stefan McGinn-Summers, Neil Beckford, Inthushaa Indrakumar and Kristina Lakey (Departmental administrative staff in AGE); Dinithi Perera (departmental manager); Heather McLellan-Young (project manager); Helen Ward, James McKeand, Geraint Price, Josip Car, Christina Atchison, Nicholas Peters, Aldo Faisal, and Jennifer Quint (investigator team contributing to CCRR survey design, development and improvement).

Patient and public involvement:

Older adult volunteers (60-80 years of age) from various social and cultural backgrounds provided feedback on the survey content. This feedback was incorporated into the survey design.

Conflicts of Interest

All authors have completed the ICMJE uniform disclosure form at www.icmje.org/coi_disclosure.pdf and declare: no support from any organisation for the submitted work; Lefkos T. Middleton reports research funding from Janssen, Novartis, Merck and Takeda, outside the submitted work.

Licence

The Corresponding Author has the right to grant on behalf of all authors and does grant on behalf of all authors, a worldwide licence to the Publishers and its licensees in perpetuity, in all forms, formats and media (whether known now or created in the future), to i) publish, reproduce, distribute, display and store the Contribution, ii) translate the Contribution into other languages, create adaptations, reprints, include within collections and create summaries, extracts and/or, abstracts of the Contribution, iii) create any other derivative work(s) based on the Contribution, iv) to exploit all subsidiary rights in the Contribution, v) the inclusion of electronic links from the Contribution to third party material where-ever it may be located; and, vi) licence any third party to do any or all of the above.

For peer review only

Abstract

Objectives: Physical inactivity is more common in older adults, is associated with social isolation and loneliness, and contributes to increased morbidity and mortality. We examined the effect of social restrictions to reduce COVID-19 transmission in the UK (lockdown), on physical activity (PA) levels of older adults, and the social predictors of any change.

Design: Baseline analysis of a survey-based prospective cohort study

Setting: Adults enrolled in the Cognitive Health in Ageing Register for Investigational and Observational Trials (CHARIOT) cohort from General Practitioner (GP) practices in North West London were invited to participate from April to July 2020.

Participants: 6,219 cognitively healthy adults aged 50 to 92 years completed the survey.

Main outcome measures: Self-reported PA before and after the introduction of lockdown, as measured by Metabolic Equivalent of Task (MET) minutes. Associations of PA with demographic, lifestyle and social factors, mood and frailty.

Results: Mean PA was significantly lower following the introduction of lockdown, from 3,519 MET minutes/week to 3,185 MET minutes/week ($p<0.001$). After adjustment for confounders and pre-lockdown PA, lower levels of PA after the introduction of lockdown were found in those who were over 85 years old (640 [95% CI: 246 to 1034] MET minutes/week less); were divorced or single (240 [95% CI: 120 to 360] MET minutes/week less); living alone (277 [95% CI: 152 to 402] MET minutes/week less); reported feeling lonely often (306 [95% CI: 60 to 552] MET minutes/week less); and showed symptoms of depression (1007 [95% CI: 1401 to 612] MET minutes/week less) compared to those aged 50-64 years, married, co-habiting, and not reporting loneliness or depression, respectively.

Conclusions and Implications: Markers of social isolation, loneliness and depression were associated with lower PA following the introduction of lockdown in the UK. Targeted interventions to increase PA in these groups should be considered.

Strengths and limitations of this study:

- Out of 40,000 people contacted, 7320 responded and 6219 completed the survey
- A significant reduction in mean levels of physical activity were found in older adults after the introduction of lockdown measures.
- Multivariable analyses were adjusted for confounders according to pre-determined causal pathways

For peer review only - http://bmjopen.bmj.com/site/about/guidelines.xhtml

For peer review only

- Survey responders identified predominantly as White/Caucasian background, and showed higher levels of physical activity than the general population, which may limit the generalisability of the findings to other population groups
- The potential for recall bias from using a self-report questionnaire for physical activity levels (International Physical Activity Questionnaire – IPAQ). This includes reliance on recall for pre-lockdown PA levels

For peer review only

For peer review only - http://bmjopen.bmj.com/site/about/guidelines.xhtml

1.0 Background and Rationale

Physical inactivity adversely affects older adults, with 60-70% of those aged over 75 years not sufficiently physically active for good health[1,2] as defined by meeting World Health Organization (WHO)[3] and UK[4] guidelines. From March until June 2020 in the UK, a national 'lockdown' was implemented to reduce exposure to, and transmission of, COVID-19. Although applied to the whole population, adults aged over 70 years and those with underlying health conditions at higher risk of severe COVID-19 disease were asked to follow more stringent social distancing measures. These included remaining at home where possible; avoiding social mixing in the community; avoiding physically interacting with friends and family; and avoiding public transport (supplementary file 1: figure S1).[5]

Social isolation and loneliness in older adults, possibly exacerbated during lockdowns,[6] is associated with increases in morbidity and mortality, increased physical inactivity and sedentary time,[7,8] and reduced physical performance.[9] Physical inactivity may therefore have a role in mediating the increased morbidity and mortality associated with social isolation.[10] Physical activity (PA) is important in the prevention of sarcopenia, frailty and decreased functional ability in older adults.[11] Data collected on the pandemic, predominantly in younger adults and children, suggests a decrease in PA and an increase in sedentary time.[12] Given the increased susceptibility to physical inactivity and social isolation in older adults in particular, this is an important area of study.[13] We set up the CHARIOT COVID-19 Rapid Response study (CCRR) in April 2020 to monitor symptoms and the impact of the COVID-19 pandemic on various health and lifestyle factors, by repeat questionnaire survey of the Cognitive Health in Ageing Register for Investigational and Observational Trials (CHARIOT) members.

We hypothesised that imposed social restrictions would negatively impact on PA levels of older adults, and that change in PA after the introduction of lockdown would be modified by certain demographic, lifestyle and social factors, with a focus on markers of social isolation and perceived loneliness. An awareness of the extent of, and predictors for, change in PA levels will aid our understanding of the impact of social isolation on the health of older adults, both with respect to pandemic-related lockdowns and social isolation itself.

2.0 Methods

2.1 CCRR survey

For peer review only - http://bmjopen.bmj.com/site/about/guidelines.xhtml

Study participants were recruited from the CHARIOT register, a cohort of over 40,000 cognitively healthy (without a known diagnosis of dementia) adult volunteers aged over 50 years, recruited from 172 GP surgeries across West and North London as part of a collaboration between regional GP practices and the School of Public Health, at Imperial College London.

This ongoing prospective cohort study was initiated in April 2020 with repeated questionnaire surveys conducted every six weeks. The CCRR baseline survey consists of questions related to basic demographics, diet, alcohol and smoking status, symptoms of COVID-19, functional activities, physical activity, sleep, frailty and mental health (supplementary file 2). For physical activity, the International Physical Activity Questionnaire (IPAQ) short-form (last 7 days) was used,[14] asking respondents to document their weekly vigorous and moderate activity, walking and sitting time from the week prior to completing the survey; and for the week prior to implementation of social restriction measures. This has test-retest reliability of 0.75 in those under the age of 60 years.[15] However, although less commonly studied in older populations, one study demonstrated reduced reliability, at 0.65 and 0.57 for men and women respectively aged 65-74 years, and 0.50 and 0.56 for those aged 75-89 years, but with adequate validity when assessed against objective measures.[16] For assessing frailty, the 5-point FRAIL scale,[17,18] (ordinal scale 1-5; predictive validity for mortality up to 10 years; HR: 2.60)[19] and for assessing mental health symptoms, the Hospital Anxiety and Depression (HADS) scale;[20] sensitivity and specificity 0.8 for both anxiety and depression;[21] 14 questions on feelings related to anxiety and depression rated on a 4-level Likert scale) were used. A question on loneliness was used from the Imperial College Sleep Quality questionnaire; in turn adapted from the Pittsburgh Sleep Quality Index[22] and Centre for Epidemiologic Studies of Depression Scale[23], for work-free periods.

Participants were eligible for recruitment if they were participating in the CHARIOT Register, or were a consenting member of the household of a participant who wished to take part; had mental capacity to consent to participate; were willing and able to undertake an electronic questionnaire survey; were able to read, write and were fluent in English, or identify an informant who was. Participants were excluded where they were no longer participating in the CHARIOT register, or if they did not have access to electronic devices to complete the questionnaire surveys. Survey data used in the present analysis were completed between 30th April and the 22nd July 2020, and a timeline of lockdown measures has been incorporated into the supplementary data (supplementary file 1: figure S1)

For peer review only - http://bmjopen.bmj.com/site/about/guidelines.xhtml

This research was approved by the Imperial College Research and Ethics Committee (ICREC) and Joint Research Compliance Office (22/04/2020; 20IC5942)

2.2 Statistical analysis

All analyses were conducted using Stata version 16.1 (StataCorp 2019) and R.[24,25] Body Mass Index (BMI) was calculated as weight in kilograms divided by the square of height in metres and categorised according to standard WHO criteria. IPAQ data were cleaned according to the IPAQ data cleaning protocol,[26] and the Metabolic Equivalent of Task (MET) minutes per week, calculated for each activity and total activity (where 3.3 METS is considered equivalent to walking, and moderate and vigorous activity equivalent to 4 and 8 METS, respectively). Periods of activity under 10 minutes were excluded as per the protocol, excluding for vigorous, moderate and walking activities during lockdown, 25, 23 and 12 periods, respectively (for pre-lockdown activity, excluding 10, 13 and 3 periods of activity, respectively). To calculate the total MET minutes per week, the self-reported duration (minutes) and frequency (days) of each of these PA categories is multiplied by the specified metric (supplementary file 1: supplementary methods). Paired t-tests were used to compare the distributions of mean PA levels pre- and following the introduction of lockdown.

Measures of association with explanatory variables were explored in univariable linear regression models for two outcomes: i) overall weekly MET minutes after introduction of lockdown and ii) the difference in overall weekly MET minutes before versus after the introduction of lockdown. Multivariable models were constructed for the outcome of MET minutes after the introduction of lockdown, adjusting *a priori* each explanatory variable in turn for age, sex and ethnicity. Month of survey completion was also included in the model to account for seasonal changes, and the finding that physical activity after the introduction of lockdown varied by month (supplementary file 1: figure S2 and table S1). Weekly MET minutes before the introduction of lockdown was also included in the model given its strong association with activity levels after the introduction of lockdown, which remained significantly associated in all models. Denominators for each model vary according to the levels of missingness in variables included in the models, which was low for most variables, except for BMI (unrecorded in 51.4% of participants). Employment was re-categorised into four groups for the purposes of regression analysis (supplementary file 1: Table S2).

A causal diagram was constructed using DAGitty[27] (supplementary file 1: figure S3) to aid adjustment for confounders in order to separate the overall causal effects of marital status, loneliness and living alone on physical activity. Additional multivariable models were then constructed based on the causal diagram for loneliness, adjusting for age, sex, ethnicity,

For peer review only - http://bmjopen.bmj.com/site/about/guidelines.xhtml

household status, marital status, shielding status and frailty category. No further adjustment was necessary for marital status or household status. Residuals were plotted against fitted values to assess for outlying points and heteroskedasticity; and plots of Cook's distance and leverage against fitted values were examined to detect the presence of influential points.

2.3 Patient and public involvement:

Older adult volunteers (60-80 years of age) from various social and cultural backgrounds provided feedback on the survey content. This feedback was incorporated into the survey design. Participants in the CHARIOT cohort are informed by regular newsletter of all publications pertaining to the cohort.

For peer review only

3.0 Results

3.1 Participant characteristics

The survey was sent to 15,000 CHARIOT participants via email, with a subsequent 25,000 contacted by post. 7,320 participants responded and completed the survey. Of these respondents, 6,219 completed IPAQ data both before and after introduction of lockdown measures and were included in the final analysis.

Of the 6,219 participants included in the present study, 55.4% were female, and the majority (55.3%) were aged 65-74 years with a mean age of 70 years. 93.7% of respondents classified themselves as being of white ethnic background, with 2.8% of Asian ethnic background, and only 0.7% of black African or Caribbean background. Approximately half of participants (48.6%) had a recorded height and weight, with a mean BMI of 25.3 kg/m^2. The majority of respondents were married (62.2%), co-habiting (72.8%) and retired (69.5%). Most respondents did not smoke (96.9%), drank alcohol (82.6%) and felt they ate a healthy diet (80.3%). 18.0% of respondents were classified as pre-frail, with 0.5% as frail and 26.2% reported that they were shielding at the time of the survey (table 1).

Participant characteristic		Total	Percent
**Gender**	Female	3,445	55.4%
	Male	2,770	44.5%
	Prefer not to say	4	0.1%
**Age (years)**	Mean (SD)		69.9 (7.3)
	Median (IQR)		70 (66-74)
	Range		50 - 92
	50-64	1,212	19.5%
	65-74	3,440	55.3%
	75-84	1,394	22.4%
	85+	127	2.0%
	Missing data	46	0.7%

Ethnicity	**White**	**5,825**	**93.7%**
	English/Welsh/Scottish/Northern Irish/British	5,143	82.7%
	Any other white background	536	8.6%
	Irish	146	2.3%
	**Mixed/multiple ethnic groups**	**99**	**1.6%**
	White and Black African	10	0.2%
	White and Asian	33	0.5%
	White and Black Carribean	7	0.1%
	Any other mixed/multiple ethnic background	49	0.8%
	**Asian/Asian British**	**174**	2.8%
	Indian	91	1.5%
	Pakistani	12	0.2%
	Bangladeshi	2	0.0%
	Chinese	32	0.5%
	Any other Asian background	37	0.6%
	**Black/African/Caribbean/Black British**	**43**	**0.7%**
	African	13	0.2%
	Carribean	21	0.3%
	Any other Black/African/Caribbean/Black British	9	0.1%
	**Other ethnic group**	**64**	**1.0%**
	Arab	17	0.3%
	Any other ethnic group	47	0.8%
	**Prefer not to say**	**14**	**0.2%**
Body Mass Index (BMI) (Kg/m2)	Mean (SD)		25.3 (5.1)
	Median (IQR)		24.4 (22.2-27.1)
	<18.5 (underweight range)	61	1.0%
	18.5-24.9 (healthy weight)	1,644	26.4%
	25.0-29.9 (overweight)	962	15.5%
	>=30.0 (obese range)	358	5.8%
	Missing data	3,194	51.4%
Shielding at time of questionnaire	No	4,591	73.8%
	Yes	1,628	26.2%
Marital status	Married	3,869	62.2%
	Single	789	12.7%
	Widowed	601	9.7%
	Divorced	595	9.6%
	Living with a partner	365	5.9%
Living arrangements	Co-habiting	4,530	72.8%
	Living alone	1,689	27.2%
Employment	Retired	4,322	69.5%
	Continuing to work in your usual job; at home	1,101	17.7%
	None of the above	201	3.2%
	Furloughed (put on leave, still getting paid	197	3.2%

For peer review only - http://bmjopen.bmj.com/site/about/guidelines.xhtml

	Continuing to work in your usual job and leave home for your job	141	2.3%
	A key worker	96	1.5%
	Had to close your business due to COVID-19	70	1.1%
	Lost my job due to the lockdown	42	0.7%
	Unemployed	36	0.6%
	A student	13	0.2%
**Current smoker**	No	6,027	96.9%
	Yes	192	3.1%
**Alcohol intake**	No	1,083	17.4%
	Yes	5,136	82.6%
**Diet**	No change from usual - already had a healthy diet	4,991	80.3%
	My diet has become more healthy	715	11.5%
	My diet was healthy before but has got worse since lockdown	312	5.0%
	No change from usual - my diet isn't very healthy	201	3.2%
**FRAIL scale**	Robust	5,055	81.3%
	Pre-frail	1,117	18.0%
	Frail	34	0.5%
	Missing data	13	0.2%
**Loneliness**	Not ever	2,994	48.1%
	Rarely	1,469	23.6%
	Sometimes	1,305	21.0%
	Often	372	6.0%
	Missing data	79	1.3%
**HADS (depression score)**	Normal (0-7)	4,658	74.9%
	Borderline (8-10)	312	5.0%
	Abnormal (11-21)	116	1.9%
	Missing data	1,133	18.2%
**HADS (anxiety score)**	Normal (0-7)	4,335	69.7%
	Borderline (8-10)	486	7.8%
	Abnormal (11-21)	265	4.3%
	Missing data	1133	18.2%
**Total participants**		**6,219**	

Table 1: Participant characteristics for 6,219 participants with complete data on physical activity; HADS – Hospital Anxiety and Depression Score

3.2 Physical activity before and after social distancing measures

Mean (SD) PA for participants prior to lockdown was 3,519 (2867) MET minutes/week. There was a significant reduction in mean MET minutes following implementation of lockdown to 3,185 (2673) MET minutes/week (p<0.001; table 2 & figure 1). 3,167 (50.9%) participants

For peer review only - http://bmjopen.bmj.com/site/about/guidelines.xhtml

decreased their activity following the introduction of lockdown by a mean (SD) of 1,957 (2025) MET minutes/week, 534 (8.6%) maintained the same level of activity, and 2,518 (40.5%) increased activity by a mean (SD) of 1,636 (1775) MET minutes/week. Mean sitting time increased by 276 MET minutes/week after the introduction of lockdown (2,680) compared to before (2,404) (table 2).

5,762 (92.7%) participants achieved at least the minimum guidance of 600 MET minutes/week of activity, as defined by WHO,[3] prior to implementation of lockdown measures, slightly reducing to 5,672 (91.2%) following their introduction (p<0.001). 5,039 (81.0%) achieved 1,200 MET minutes/week before lockdown, with 4,904 (78.9%) achieving this after the introduction of lockdown (p<0.001, supplementary file 1: figure S4). Following the introduction of lockdown, PA levels varied by month of survey completion, with the highest levels in June and lowest levels in July. There was no significant difference between self-reported PA before lockdown by month of survey completion (supplementary file 1: figure S5).

For peer review only

Physical activity type		Before	During	p value for difference
**Vigorous activity**	Mean (SD) minutes/week	145 (276)	135 (253)	0.004
	Median (IQR) minutes/week	40 (0 - 180)	10 (0 - 180)	
**Moderate activity (minutes/week)**	Mean (SD) minutes/week	292 (430)	245 (374)	<0.001
	Median (IQR) minutes/week	120 (0 - 360)	120 (0-360)	
**Walking (minutes/week)**	Mean (SD) minutes/week	462 (460)	403 (408)	<0.001
	Median (IQR) minutes/week	360 (150 - 630)	315 (150 - 525)	
**Sitting (minutes/week) ***	Mean (SD) minutes/week	2404 (1137)	2680 (1181)	<0.001
	Median (IQR) minutes/week	2100 (1680 - 2940)	2520 (1680 - 3360)	
**MET minutes/week**	Mean (SD) minutes/week	3519 (2867)	3185 (2673)	<0.001
	Median (IQR) minutes/week	2772 (1386 - 4650)	2440 (1386 - 4185)	

Table 2: Physical activity and sitting time for recipients before and following introduction of lockdown measures. Data presented as minutes per week with both mean (standard

For peer review only - http://bmjopen.bmj.com/site/about/guidelines.xhtml

deviation) and median (interquartile range) shown. p-values from paired t-test; *denominator 6,023; MET - Metabolic Equivalent of Task

3.3 Predictors of physical activity after the introduction of lockdown, and change from before lockdown

3.3.1 Demographic and lifestyle factors

Univariable linear regression models (supplementary file 1: table S3) showed statistically significant associations with lower PA after the introduction of lockdown in older age groups (p<0.001; figure 1), but no evidence of differences in the change from before lockdown between age groups (p=0.184; figure 2). After multivariable adjustment for age, sex, ethnicity, month of survey completion and pre-lockdown physical activity (supplementary file 1: table S4) there was evidence of significantly lower levels of PA with increasing age, with adults aged 85 years and over doing on average 640 (95% CI: 246 to 1034) MET minutes/week less than those aged 50-64 years (figure 3). There was no significant difference in PA after the introduction of lockdown in males and females (p=0.180; figure 1), but females on average exhibited a greater decline in PA from before lockdown than males (450 vs 189 MET minutes/week less respectively; p<0.001; figure2). After multivariable adjustment, there was only a small and borderline significant difference in PA after lockdown was introduced between gender (PA in males on average 108 MET minutes/week more than females; 95% CI: -1 to 216; figure 3). No significant associations were seen between PA after the introduction of lockdown or change in PA according to ethnicity or employment status, before or after adjustment.

Lower levels of PA after the introduction of lockdown were seen with increasing BMI category, in current smokers and in those reporting an unhealthy or worsening diet before and after adjustment (figure 1). After adjustment, a dose-response relationship was evident between lower PA and increasing BMI (p=0.030), with obese individuals doing 578 (95% CI: 324 to 832) MET minutes/week less than those of a healthy weight (figure 3). The denominator included in analyses of BMI was significantly lower than for other models, as BMI was unrecorded for 51.4% of participants. Current alcohol consumption was weakly associated with increased levels of PA in both univariable and multivariable models, with current drinkers reporting 145 MET minutes/week more than non-drinkers after adjustment (95% CI: 1 to 289; figures 2 & 3).

3.3.2 Associations with social isolation and loneliness

For peer review only - http://bmjopen.bmj.com/site/about/guidelines.xhtml

Participants who were divorced, single or widowed were, on average, less active after the introduction of lockdown than those married or living with a partner (3,026 vs 3,262 MET minutes/week; p=0.001; figure 1); and exhibited a greater decline in PA from before lockdown (540 vs 236 MET minutes/week less; p<0.001; figure 2). The association with PA after the introduction of lockdown remained after adjustment, with those divorced, single or widowed doing on average 240 (95% CI: 120 to 360) MET minutes/week less (figure 3). Participants living alone were also less active than those co-habiting and showed greater reductions in PA from before lockdown. After adjustment for confounders and PA before lockdown, those living alone were doing 277 (95% CI: 152 to 402) MET minutes/week less than those co-habiting (figure 3).

Significant associations were seen between PA after the introduction of lockdown and frequency of loneliness, with those 'often' experiencing loneliness achieving 2,938 MET minutes/week compared with 3,284 MET minutes/week in those 'never' experiencing loneliness (p=0.024; figure 1). Greater declines in PA from before lockdown were also seen with increasing loneliness (figure 2). After adjustment, PA after the introduction of lockdown was significantly lower for those with increased frequency of loneliness (figure 3). After full adjustment including, in addition, household status, marital status, shielding status and frailty category, those experiencing loneliness 'often' reported 306 (95% CI: 60 to 552) MET minutes/week less activity than those 'never' lonely (supplementary file 1: table S5).

Significantly lower physical activity levels were recorded in those shielding and in participants categorised as pre-frail or frail (both p<0.001; figure 1). Larger declines in PA from before lockdown were seen in those shielding compared to those not shielding (588 vs 243 MET minutes/week less; p<0.001; figure 2), but there was no significant difference in change in PA according to frailty category (p=0.389; figure 2). After adjustment, frail participants were doing 926 (95% CI: 189 to 1,663) MET minutes less on average than those classed as robust (figure 3). Participants who were shielding were doing an average of 290 (95% CI: 163 to 417) MET minutes/week less than those not shielding (figure 3).

3.3.3 Associations with depression and anxiety

Symptoms of depression were associated with lower levels of PA following the introduction of lockdown, with those meeting the criteria for depression reporting 2,450 MET minutes/week compared to 3,195 MET minutes/week in those with normal scores (p<0.001; figure 1). There was no strong association with anxiety scores. Mean change in PA from before lockdown was associated with both depression and, in contrast to absolute PA levels, with anxiety scores. Participants with depression reported 1,450 MET minutes/week less on average after lockdown was introduced compared with before, while those with normal scores

For peer review only - http://bmjopen.bmj.com/site/about/guidelines.xhtml

reported 293 MET minutes/week less ($p<0.001$; figure 2). Similarly, in those with anxiety, PA reduced by 836 MET minutes/week compared to 312 MET minutes/week in those with normal scores ($p=0.004$; figure 2).

After adjustment, those meeting the criteria for depression on the HADS scale had significantly lower PA levels than those with normal scores, doing on average 1,007 (95% CI: 1401 to 612) MET minutes/week less (figure 3). There remained no statistically significant association between anxiety score and physical activity after adjustment.

4.0 Discussion

4.1 Main findings

Data from the CCRR study show that participants experienced, on average, a significant decrease in PA after the introduction of lockdown in the UK when compared with before, together with an increase in sitting time. When adjusted for age, sex, ethnicity, month of survey completion and baseline physical activity, factors strongly associated with a reduction in PA include; increased age, increased BMI, frailty, current smoking, and a change to a less healthy diet. Factors associated with social isolation were also significantly associated with a reduction in PA: those divorced, single or widowed, living alone, shielding or reporting increased frequency of loneliness did significantly less PA after lockdown was introduced. Furthermore, a strong association was also seen with lower PA following the introduction of lockdown in those with depression, but not for those with anxiety.

4.2 The effect of lockdown on physical activity

There was a reduction in PA in over half of our participants, and a decrease in mean levels of PA by 333 MET minutes/week following the introduction of lockdown measures in the UK. This was accompanied by an increase in sitting time by 276 minutes per week, an adverse finding given the adverse health impacts associated with increased sedentary and sitting time.[28] These findings correlate with other studies from the UK (a decrease in 25% of adults aged over 20 years following lockdown),[29] Spain[30] and China,[31] and from a global survey collected in 8 different languages,[32] despite the differences in outdoor exercise permissions between countries. Reductions in PA may impact disproportionately across society. We found that increasing age associated with a reduction in PA after lockdown was introduced, corresponding with that seen in Japan, with a 26.5% (65 minutes) decrease in total physical activity in adults aged 65 to 84.[33] The UK Active Lives Survey found a 7.3% reduction in the proportion of active adults aged 55-74 years, from 63% to 56%, during the pandemic, and a 6.6% reduction in those aged 75 years and above, from 42% to 35%.[34] A self-reported study

For peer review only - http://bmjopen.bmj.com/site/about/guidelines.xhtml

in the UK found that those with a diagnosis of obesity, hypertension, lung disease, depression or a disability were more likely to reduce PA during lockdown.[29]

4.3 Social relationships, loneliness, and physical activity

Individuals for whom social engagement was more likely to be restricted, such as those who were shielding, divorced, single, widowed, or living alone, were more likely to have lower levels of PA after lockdown implementation, and to have declined to a greater extent. Similarly, those who subjectively reported feeling lonely were more likely to have lower PA levels, and greater declines from before lockdown. These associations remained significant after multivariable adjustment.

Associations between health behaviours, including PA, and social relationships have been noted previously. Data from the English Longitudinal Study of Ageing (ELSA) showed that socially isolated respondents were less likely to report healthy diets, and more likely to smoke.[7] Crucially, they showed reduced activity counts in socially isolated individuals (measured by accelerometer) in a sample of adults older than 50 years,[8] and reduced self-reported moderate to vigorous physical activity.[7] This is particularly important given that isolated and lonely individuals are at an increased risk of morbidity and mortality from cardiovascular events, with the majority of this association mediated by risk factors which include physical inactivity.[35] Fixed effect models from the ELSA cohort show that social disengagement, domestic isolation and loneliness are associated with measures of poorer physical performance, and although they appear to be independent of physical activity, may still be associated along the causal pathway.[9] Studies of spousal pairs found that both men and women in married couples had greater levels of PA than their single counterparts,[36] and changes in PA are positively associated with changes in the PA of a spouse.[37] Increasing PA is associated with larger,[38,39] more diverse[40] and more heterogenous (in terms of PA) social networks, and having more physically active people in a social network is associated with being more active.[41]

The interaction between social relationships and PA levels may be bi-directional. Levels of PA are influenced by multiple factors at different levels, including individual (psychological, genetic); interpersonal (social networks); environmental (social, built, natural); and regional or global determinants.[42] Social networks might influence PA through social support for individuals to take up and maintain activity, but also by regulating social norms, and associating PA with social connections or attachments.[43] There may also be increased opportunities for PA[41] when social networks are present.

4.4 Mood and physical activity

For peer review only - http://bmjopen.bmj.com/site/about/guidelines.xhtml

In those reporting symptoms of depression, there were significantly lower levels of PA and a significant decrease in activity when compared to before lockdown. These findings correlate with those from the UK,[44] Australia,[45] and Spain,[46] which found inverse associations between physical activity levels and poor mental health. Similarly, a cross sectional study of Brazilian adults who were self-isolating found lower odds of symptoms of anxiety or depression in those who were performing over 30 or 15 minutes per day of moderate or vigorous activity respectively, and higher odds in those with prolonged sedentary time over 10 hours.[47] The associations between PA and mental health are well known, with positive impacts on wellbeing,[48] and reduced incidence and severity of symptoms of mental ill-health.[49–51] Therefore, these findings are unsurprising, although the interaction between PA and reduced markers of mental ill-health in older adults may be bi-directional. Moreover, social isolation and loneliness may mediate some of this effect: previous data from the CCRR cohort showed an interaction between social isolation, loneliness, and female gender with worsening depression and anxiety over lockdown.[52] We found no statistically significant difference in PA following the introduction of lockdown with anxiety symptoms, at odds with previous studies.[44] However, the trajectory of anxiety symptoms is not known, and it is not clear whether anxiety symptoms pre-dated the introduction of lockdown.

4.5 Health behaviours and physical activity

A decrease in PA was associated with other detrimental health behaviours, including unhealthy diet and smoking. A similar tendency of clustering of unhealthy behaviours during the COVID-19 pandemic was noted in a cohort of patients in Spain with type 2 diabetes mellitus, who showed an increase in sugary foods and snack consumption alongside an increase in sitting time, and a decrease in time spent walking or doing moderate physical activity during lockdown when compared to beforehand.[53] That detrimental health behaviours might coincide in response to lockdown shows the importance of targeted interventions for certain groups. Interestingly, alcohol consumption was seen to be a protective factor in our cohort, and this does not tie with other findings on the negative associations with increased alcohol use during the COVID-19 pandemic.[54] This may be due to the specific demographic features of our cohort, but the possibility of alcohol consumption being associated with social interaction in this group cannot be excluded.

4.6 Limitations

This study has several limitations which may impact the generalisability of our findings. First, the CCRR cohort appear more physically active than the general population. 90% of participants in CCRR achieved minimum WHO (2010) [3] guidance, both before and

For peer review only - http://bmjopen.bmj.com/site/about/guidelines.xhtml

For peer review only

following the introduction of lockdown. Over 78% achieved double this amount, and mean levels of PA were at least five times greater than the minimum recommendation. In contrast, only 61% of UK adults aged 55-74 years achieve minimum recommended WHO (2010) levels.[2] Despite this, CCRR participants may still not be active enough for major health gains. A 2016 systematic review and meta-analysis suggested that optimal risk reduction for breast and colorectal cancer, diabetes, ischaemic heart disease and stroke events were obtained from physical activity at 3000-4000 MET minutes per week.[55]

Second, there are differences in demography between the CCRR cohort and the general population of the UK, which may explain the higher levels of PA we observed. 93% of CCRR respondents identify as white/Caucasian ethnicity. The Active Lives Survey demonstrated a difference in those achieving minimum activity levels in White British individuals (65%) and those from Black (58%) and Asian (54%) ethnicities.[2] Third, the CCRR survey relies on self-report, using the short form IPAQ. IPAQ data is well validated across diverse participants up to the age of 65 years [14] and a study of the performance of the IPAQ in older Japanese adults demonstrated adequate validity.[16] However, results from self-reporting tools for PA only weakly correlate with those from objective measures, such as accelerometers and pedometers.[56–59] Finally, recall bias and seasonal changes in physical activity may also have impacted on the results, with the additional factor that data were collected remotely rather than face to face (although there was necessary due to pandemic control measures). The CCRR survey was collected in April-July 2020, with participants asked to recall PA levels in the week before lockdown, which over time may become less reliable. However, no significant differences were found in the mean PA levels reported before lockdown according to month of survey completion and although there were apparent differences in PA following the introduction of lockdown by month, we were able to adjust for this in multivariable models. Furthermore, social restriction measures are dynamic and change over time, with a loosening of restrictions by 4th July 2020, and as a result the majority of the small proportion of respondents from July were reported outside of actual lockdown measures. However, changes to physical activity may persist, and the CCRR prospective cohort study is ongoing, with follow-up questionnaires sent to participants at regular intervals. When complete this will allow for long-term impacts to be measured, accounting for seasonal variation and changes to restriction measures over time.

4.7 Conclusions

Findings from our CCRR study suggest a significant decline in average PA levels in older adults following the introduction of lockdown measures during the COVID-19 pandemic. These are in keeping with similar decreases across age ranges, including healthy

For peer review only - http://bmjopen.bmj.com/site/about/guidelines.xhtml

adults, children and adolescents, and in those with medical conditions,[12] and are particularly concerning given the negative health connotations of physical inactivity. Moreover, even before the pandemic, older adults were more physically inactive than younger individuals, with only 61% and 40% of those aged 55-74 and 75 years old respectively meeting recommended levels of PA.[2]

In our study, lower activity levels after the introduction of lockdown were strongly linked to older age, and to those with objective markers of social isolation, subjective feelings of loneliness and symptoms of depression. Strategies and targeted interventions to increase and sustain PA levels in older adults are needed to mitigate the adverse health impacts not only of COVID-19 related lockdowns, but of social isolation in general. A recent systematic review suggested that digital behavioural change interventions can increase physical activity levels, and decrease sedentary time, in older adults, and this may be an area of future research for physical activity in the context of social isolation.[11,60] Although there can be no 'one size fits all' approach,[13] interventions should consider social relationships in their design and implementation.

6.0 References

1 NHS Digital. Health Survey for England 2016 Physical activity in adults. 2017 https://files.digital.nhs.uk/publication/m/3/hse16-adult-phy-act.pdf.

2 Sport England. Active Lives Adult Survey November 2018/19 Report. 2020 https://sportengland-production-files.s3.eu-west-2.amazonaws.com/s3fs-public/2020-04/Active Lives Adult November 18-19 Report..pdf?BhkAy2K28pd9bDEz_NuisHl2ppuqJtpZ.

3 World Health Organization. Global Recommendations on Physical Activity for Health. 2010 https://www.who.int/publications/i/item/9789241599979.

4 Davies SC, Atherton F, McBride M, Calderwood C. UK Chief Medical Officers' Physical Activity Guidelines. 2019.

5 Gov.uk. UK Government COVID-19 guidance. .

6 Wu B. Social isolation and loneliness among older adults in the context of COVID-19: a global challenge. *Glob Heal Res Policy* 2020; **5**: 27.

7 Kobayashi LC, Steptoe A. Social Isolation, Loneliness, and Health Behaviors at Older Ages: Longitudinal Cohort Study. *Ann Behav Med* 2018; **52**: 582–93.

8 Schrempft S, Jackowska M, Hamer M, Steptoe A. Associations between social isolation, loneliness, and objective physical activity in older men and women. *BMC Public Health* 2019; **19**: 74.

9 Philip KEJ, Polkey MI, Hopkinson NS, Steptoe A, Fancourt D. Social isolation, loneliness and physical performance in older-adults: fixed effects analyses of a cohort study. *Sci Rep* 2020; **10**: 13908.

10 Elovainio M, Hakulinen C, Pulkki-Råback L, *et al.* Contribution of risk factors to excess mortality in isolated and lonely individuals: an analysis of data from the UK Biobank cohort study. *Lancet Public Heal* 2017. DOI:10.1016/S2468-2667(17)30075-0.

For peer review only - http://bmjopen.bmj.com/site/about/guidelines.xhtml

11 Izquierdo M, Duque G, Morley JE. Physical activity guidelines for older people: knowledge gaps and future directions. *Lancet Heal Longev* 2021; **2**: e380–3.

12 Stockwell S, Trott M, Tully M, *et al.* Changes in physical activity and sedentary behaviours from before to during the COVID-19 pandemic lockdown: a systematic review. *BMJ Open Sp Ex Med* 2021; **7**: 960.

13 Fakoya OA, McCorry NK, Donnelly M. Loneliness and social isolation interventions for older adults: A scoping review of reviews. *BMC Public Health* 2020; **20**: 129.

14 Craig CL, Marshall AL, Sjöström M, *et al.* International physical activity questionnaire: 12-country reliability and validity. *Med Sci Sports Exerc* 2003; **35**: 1381–95.

15 Silsbury Z, Goldsmith R, Rushton A. Systematic review of the measurement properties of self-report physical activity questionnaires in healthy adult populations. *BMJ Open* 2015; **5**: e008430.

16 Tomioka K, Iwamoto J, Saeki K, Okamoto N. Reliability and validity of the international physical activity questionnaire (IPAQ) in elderly adults: The Fujiwara-kyo study. *J Epidemiol* 2011. DOI:10.2188/jea.JE20110003.

17 Gleason LJ, Benton EA, Alvarez-Nebreda ML, Weaver MJ, Harris MB, Javedan H. FRAIL Questionnaire Screening Tool and Short-Term Outcomes in Geriatric Fracture Patients. *J Am Med Dir Assoc* 2017; **18**: 1082–6.

18 Woo J, Yu R, Wong M, Yeung F, Wong M, Lum C. Frailty screening in the community using the FRAIL scale. *J Am Med Dir Assoc* 2015. DOI:10.1016/j.jamda.2015.01.087.

19 Thompson MQ, Theou O, Tucker GR, Adams RJ, Visvanathan R. FRAIL scale: Predictive validity and diagnostic test accuracy. *Australas J Ageing* 2020; **39**: e529–36.

20 Zigmond AS, Snaith RP. The Hospital Anxiety and Depression Scale. *Acta Psychiatr Scand* 1983. DOI:10.1111/j.1600-0447.1983.tb09716.x.

21 Bjelland I, Dahl AA, Haug TT, Neckelmann D. The validity of the Hospital Anxiety and Depression Scale: An updated literature review. *J Psychosom Res* 2002; **52**: 69–77.

22 Buysse DJ, Reynolds CF, Monk TH, Berman SR, Kupfer DJ. The Pittsburgh sleep quality index: A new instrument for psychiatric practice and research. *Psychiatry Res* 1989. DOI:10.1016/0165-1781(89)90047-4.

23 Radloff LS. The CES-D Scale: A Self-Report Depression Scale for Research in the General Population. *Appl Psychol Meas* 1977. DOI:10.1177/014662167700100306.

24 R Core Team. R: A language and environment for statistical computing. R Found. Stat. Comput. 2019.

25 Viechtbauer W. Conducting meta-analyses in R with the metafor. *J Stat Softw* 2010; **36**: 1–48.

26 IPAQ scoring protocol - International Physical Activity Questionnaire. https://sites.google.com/site/theipaq/scoring-protocol (accessed Dec 1, 2020).

27 Textor J, van der Zander B, Gilthorpe MS, Liśkiewicz M, Ellison GT. Robust causal inference using directed acyclic graphs: The R package ‘dagitty’. *Int J Epidemiol* 2016. DOI:10.1093/ije/dyw341.

28 Ekelund U, Tarp J, Steene-Johannessen J, *et al.* Dose-response associations between accelerometry measured physical activity and sedentary time and all cause mortality: Systematic review and harmonised meta-analysis. *BMJ* 2019.

For peer review only - http://bmjopen.bmj.com/site/about/guidelines.xhtml

DOI:10.1136/bmj.l4570.

29 Rogers NT, Waterlow NR, Brindle H, *et al.* Behavioral Change Towards Reduced Intensity Physical Activity Is Disproportionately Prevalent Among Adults With Serious Health Issues or Self-Perception of High Risk During the UK COVID-19 Lockdown . Front. Public Heal. . 2020; **8**: 526.

30 Castañeda-Babarro A, Arbillaga-Etxarri A, Gutiérrez-Santamaría B, Coca A. Physical Activity Change during COVID-19 Confinement. *Int J Environ Res Public Health* 2020; **17**: 1–10.

31 Wang X, Lei SM, Le S, *et al.* Bidirectional Influence of the COVID-19 Pandemic Lockdowns on Health Behaviors and Quality of Life among Chinese Adults. DOI:10.3390/ijerph17155575.

32 Ammar A, Brach M, Trabelsi K, *et al.* Effects of COVID-19 Home Confinement on Eating Behaviour and Physical Activity: Results of the ECLB-COVID19 International Online Survey. *Nutrients* 2020; **12**. DOI:https://dx.doi.org/10.3390/nu12061583.

33 Yamada M, Kimura Y, Ishiyama D, *et al.* Effect of the COVID-19 Epidemic on Physical Activity in Community-Dwelling Older Adults in Japan: A Cross-Sectional Online Survey. *J Nutr Health Aging* 2020; : 1–3.

34 Active Lives Adult Survey Coronavirus (Covid-19) Report. 2020 https://sportengland-production-files.s3.eu-west-2.amazonaws.com/s3fs-public/2020-10/Active Lives Adult May 19-20 Coronavirus Report.pdf?2L6TBVV5UvCGXb_VxZcWHcfFX0_wRal7 (accessed Feb 8, 2021).

35 Hakulinen C, Pulkki-Råback L, Virtanen M, Jokela M, Kivimäki M, Elovainio M. Social isolation and loneliness as risk factors for myocardial infarction, stroke and mortality: UK Biobank cohort study of 479 054 men and women. *Heart* 2018. DOI:10.1136/heartjnl-2017-312663.

36 Pettee KK, Brach JS, Kriska AM, *et al.* Influence of marital status on physical activity levels among older adults. *Med Sci Sports Exerc* 2006; **38**: 541–6.

37 Cobb LK, Godino JG, Selvin E, Kucharska-Newton A, Coresh J, Koton S. Spousal influence on physical activity in middle-aged and older adults. *Am J Epidemiol* 2016; **183**: 444–51.

38 Marquez B, Elder JP, Arredondo EM, Madanat H, Ji M, Ayala GX. Social network characteristics associated with health promoting behaviors among Latinos. *Heal Psychol* 2014; **33**: 544–53.

39 Tamers SL, Okechukwu C, Allen J, *et al.* Are social relationships a healthy influence on obesogenic behaviors among racially/ethnically diverse and socio-economically disadvantaged residents? *Prev Med (Baltim)* 2013; **56**: 70–4.

40 Legh-Jones H, Moore S. Network social capital, social participation, and physical inactivity in an urban adult population. *Soc Sci Med* 2012; **74**: 1362–7.

41 Mötteli S, Dohle S. Egocentric social network correlates of physical activity. *J Sport Heal Sci* 2020; **9**: 339–44.

42 Bauman AE, Reis RS, Sallis JF, *et al.* Correlates of physical activity: Why are some people physically active and others not? Lancet. 2012. DOI:10.1016/S0140-6736(12)60735-1.

43 McNeill LH, Kreuter MW, Subramanian S V. Social Environment and Physical activity: A review of concepts and evidence. *Soc Sci Med* 2006; **63**: 1011–22.

For peer review only

44 Jacob L, Tully MA, Barnett Y, *et al.* The relationship between physical activity and mental health in a sample of the UK public: A cross-sectional study during the implementation of COVID-19 social distancing measures. *Ment Health Phys Act* 2020; **19**: 100345.

45 Stanton R, To QG, Khalesi S, *et al.* Depression, Anxiety and Stress during COVID-19: Associations with Changes in Physical Activity, Sleep, Tobacco and Alcohol Use in Australian Adults. *Int J Environ Res Public Health* 2020; **17**: 4065.

46 López-Bueno R, Calatayud J, Ezzatvar Y, *et al.* Association Between Current Physical Activity and Current Perceived Anxiety and Mood in the Initial Phase of COVID-19 Confinement . Front. Psychiatry . 2020; **11**: 729.

47 Schuch FB, Bulzing RA, Meyer J, *et al.* Associations of moderate to vigorous physical activity and sedentary behavior with depressive and anxiety symptoms in self-isolating people during the COVID-19 pandemic: A cross-sectional survey in Brazil. *Psychiatry Res* 2020; **292**: 113339.

48 Penedo FJ, Dahn JR. Exercise and well-being: A review of mental and physical health benefits associated with physical activity. Curr. Opin. Psychiatry. 2005. DOI:10.1097/00001504-200503000-00013.

49 Chekroud SR, Gueorguieva R, Zheutlin AB, *et al.* Association between physical exercise and mental health in 1·2 million individuals in the USA between 2011 and 2015: a cross-sectional study. *The Lancet Psychiatry* 2018. DOI:10.1016/S2215-0366(18)30227-X.

50 McDowell CP, Dishman RK, Gordon BR, Herring MP. Physical Activity and Anxiety: A Systematic Review and Meta-analysis of Prospective Cohort Studies. Am. J. Prev. Med. 2019; **57**: 545–56.

51 Schuch FB, Vancampfort D, Firth J, *et al.* Physical activity and incident depression: A meta-analysis of prospective cohort studies. *Am J Psychiatry* 2018. DOI:10.1176/appi.ajp.2018.17111194.

52 Robb CE, de Jager CA, Ahmadi-Abhari S, *et al.* Associations of Social Isolation with Anxiety and Depression During the Early COVID-19 Pandemic: A Survey of Older Adults in London, UK. *Front Psychiatry* 2020. DOI:10.3389/fpsyt.2020.591120.

53 Ruiz-Roso MB, Knott-Torcal C, Matilla-Escalante DC, *et al.* COVID-19 Lockdown and Changes of the Dietary Pattern and Physical Activity Habits in a Cohort of Patients with Type 2 Diabetes Mellitus. *Nutrients* 2020; **12**. DOI:10.3390/nu12082327.

54 Sallie SN, Ritou V, Bowden-Jones H, Voon V. Assessing international alcohol consumption patterns during isolation from the COVID-19 pandemic using an online survey: highlighting negative emotionality mechanisms. *BMJ Open* 2020; **10**: e044276.

55 Kyu HH, Bachman VF, Alexander LT, *et al.* Physical activity and risk of breast cancer, colon cancer, diabetes, ischemic heart disease, and ischemic stroke events: systematic review and dose-response meta-analysis for the Global Burden of Disease Study 2013. *BMJ* 2016; **354**: i3857.

56 Prince SA, Adamo KB, Hamel ME, Hardt J, Connor Gorber S, Tremblay M. A comparison of direct versus self-report measures for assessing physical activity in adults: a systematic review. *Int J Behav Nutr Phys Act* 2008; **5**: 56.

57 Lee PH, Macfarlane DJ, Lam TH, Stewart SM. Validity of the international physical activity questionnaire short form (IPAQ-SF): A systematic review. Int. J. Behav. Nutr.

For peer review only - http://bmjopen.bmj.com/site/about/guidelines.xhtml

Phys. Act. 2011; **8**: 115.

58 Cleland C, Ferguson S, Ellis G, Hunter RF. Validity of the International Physical Activity Questionnaire (IPAQ) for assessing moderate-to-vigorous physical activity and sedentary behaviour of older adults in the United Kingdom. *BMC Med Res Methodol* 2018; **18**. DOI:10.1186/s12874-018-0642-3.

59 Kowalski K, Rhodes R, Naylor P-J, Tuokko H, MacDonald S. Direct and indirect measurement of physical activity in older adults: a systematic review of the literature. *Int J Behav Nutr Phys Act* 2012; **9**: 148.

60 Stockwell S, Schofield P, Fisher A, *et al.* Digital behavior change interventions to promote physical activity and/or reduce sedentary behavior in older adults: A systematic review and meta-analysis. *Exp Gerontol* 2019; **120**: 68–87.

7.0 Figure legends

Figure 1: Forest plot of unadjusted univariable associations with physical activity (PA) following the introduction of lockdown measures (during/in lockdown). Data presented as mean MET minutes/week +/- 95% confidence interval. Heavy dashed line – 600 MET minutes/week (WHO minimal physical activity guideline for adults); light dashed line – mean MET minutes for the whole cohort. See also supplementary file 1: table S3; HADS – Hospital Anxiety and Depression Score; MET – Metabolic Equivalent of Task; PA – Physical Activity; WHO – World Health Organization

Figure 2: Forest plot of unadjusted mean change in physical activity (PA), following the introduction of lockdown from before, for all variables (mean MET minutes/week +/- 95% confidence interval). Negative values indicate a decline in activity after the introduction of lockdown when compared to before. See also supplementary file 1: table S3; HADS – Hospital Anxiety and Depression Score; MET – Metabolic Equivalent of Task; PA – Physical Activity

Figure 3: Forest plot of multivariable associations with physical activity after the introduction of lockdown (during/in lockdown), adjusted for age, sex, ethnicity, month of year of survey completion and baseline physical activity. Data presented as mean MET minutes/week +/- 95% confidence interval, compared to the reference group, with negative values indicating lower physical activity than the reference. See also supplementary file 1: table S4; HADS – Hospital Anxiety and Depression Score; MET – Metabolic Equivalent of Task; PA – Physical Activity

For peer review only

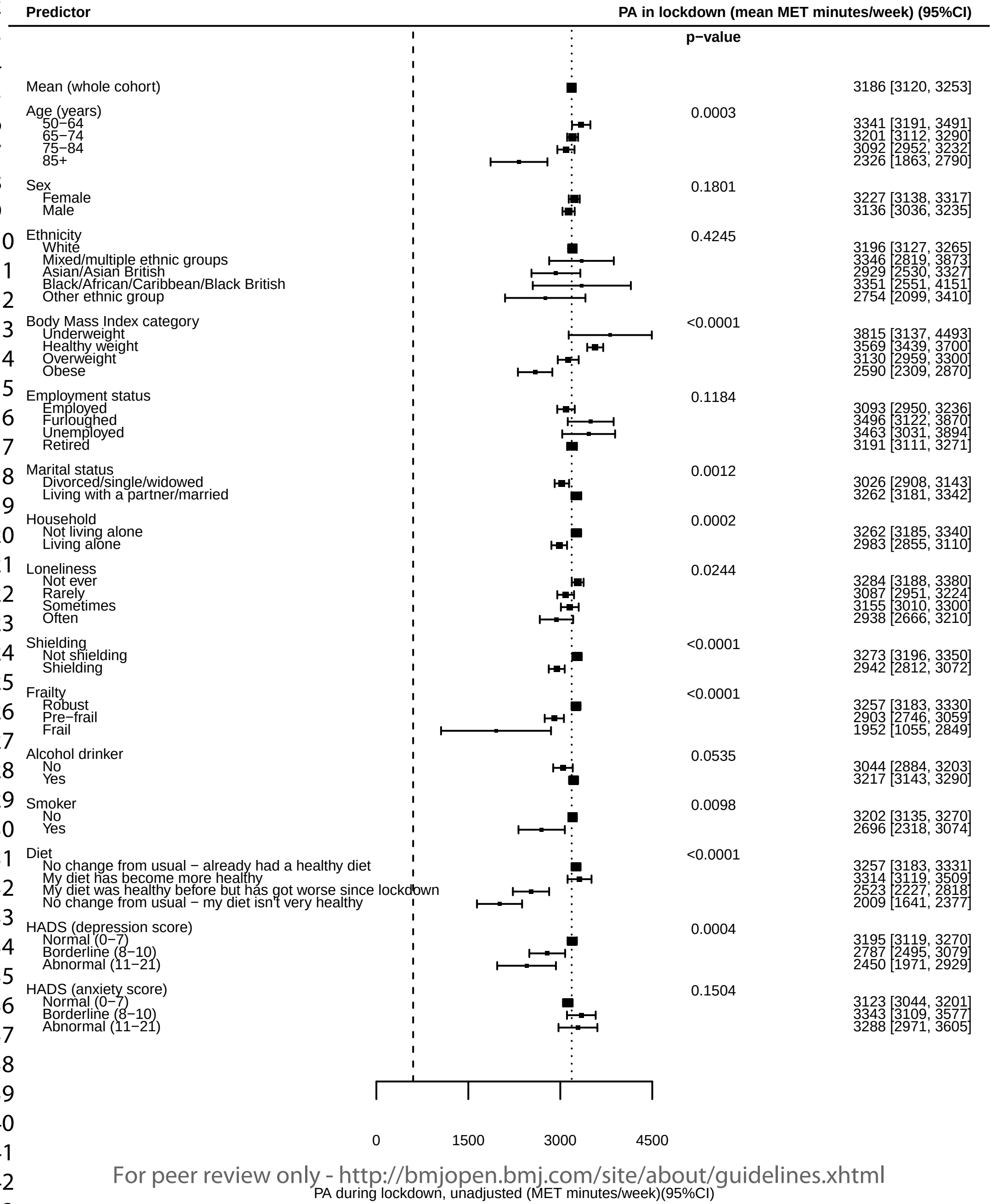

For peer review only - http://bmjopen.bmj.com/site/about/guidelines.xhtml

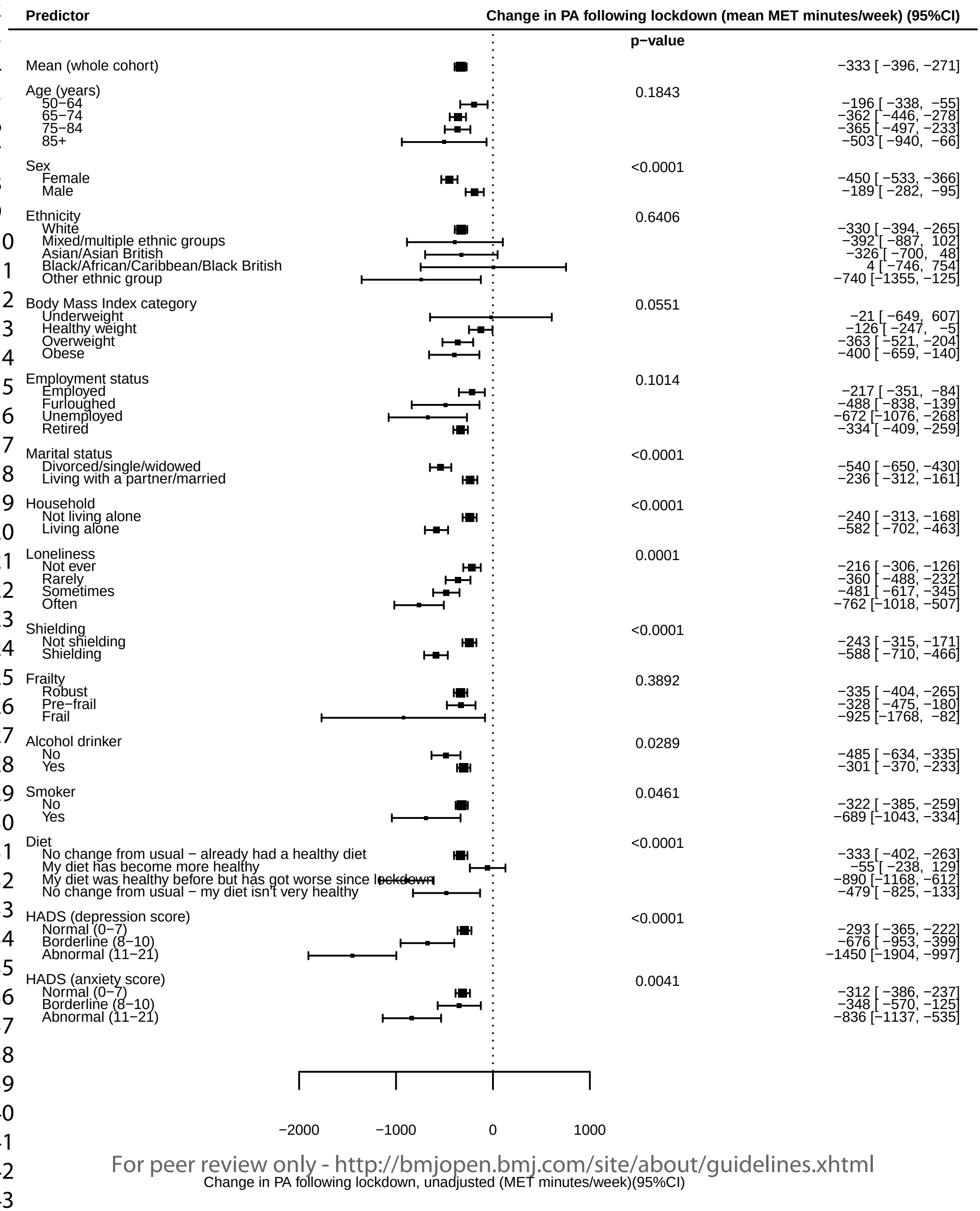
Predictor
Change in PA following lockdown (mean MET minutes/week) (95%CI)
p−value
Mean (whole cohort) −333 [−396, −271]
Age (years) 0.1843
50−64 −196 [−338, −55]
65−74 −362 [−446, −278]
75−84 −365 [−497, −233]
85+ −503 [−940, −66]
Sex <0.0001
Female −450 [−533, −366]
Male −189 [−282, −95]
Ethnicity 0.6406
White −330 [−394, −265]
Mixed/multiple ethnic groups −392 [−887, 102]
Asian/Asian British −326 [−700, 48]
Black/African/Caribbean/Black British 4 [−746, 754]
Other ethnic group −740 [−1355, −125]
Body Mass Index category 0.0551
Underweight −21 [−649, 607]
Healthy weight −126 [−247, −5]
Overweight −363 [−521, −204]
Obese −400 [−659, −140]
Employment status 0.1014
Employed −217 [−351, −84]
Furloughed −488 [−838, −139]
Unemployed −672 [−1076, −268]
Retired −334 [−409, −259]
Marital status <0.0001
Divorced/single/widowed −540 [−650, −430]
Living with a partner/married −236 [−312, −161]
Household <0.0001
Not living alone −240 [−313, −168]
Living alone −582 [−702, −463]
Loneliness 0.0001
Not ever −216 [−306, −126]
Rarely −360 [−488, −232]
Sometimes −481 [−617, −345]
Often −762 [−1018, −507]
Shielding <0.0001
Not shielding −243 [−315, −171]
Shielding −588 [−710, −466]
Frailty 0.3892
Robust −335 [−404, −265]
Pre−frail −328 [−475, −180]
Frail −925 [−1768, −82]
Alcohol drinker 0.0289
No −485 [−634, −335]
Yes −301 [−370, −233]
Smoker 0.0461
No −322 [−385, −259]
Yes −689 [−1043, −334]
Diet <0.0001
No change from usual − already had a healthy diet −333 [−402, −263]
My diet has become more healthy −55 [−238, 129]
My diet was healthy before but has got worse since lockdown −890 [−1168, −612]
No change from usual − my diet isn't very healthy −479 [−825, −133]
HADS (depression score) <0.0001
Normal (0−7) −293 [−365, −222]
Borderline (8−10) −676 [−953, −399]
Abnormal (11−21) −1450 [−1904, −997]
HADS (anxiety score) 0.0041
Normal (0−7) −312 [−386, −237]
Borderline (8−10) −348 [−570, −125]
Abnormal (11−21) −836 [−1137, −535]
−2000
−1000
0
1000
Change in PA following lockdown, unadjusted (MET minutes/week)(95%CI)

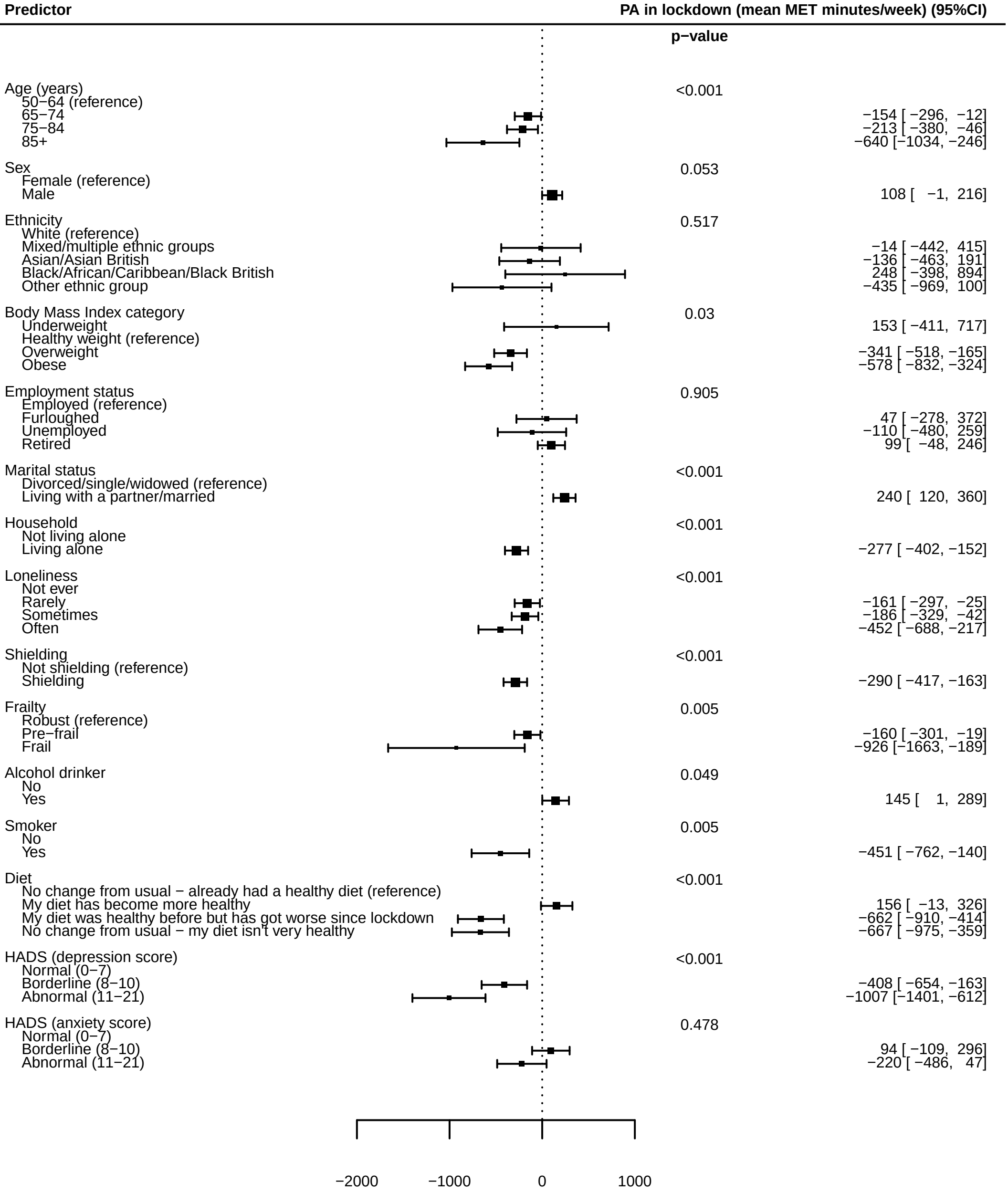

For peer review only - http://bmjopen.bmj.com/site/about/guidelines.xhtml

Multivariable associations with physical activity during lockdown (MET minutes/week)(95%CI)

Supplementary file 1

Supplementary methods

Metabolic Equivalent of Task (MET) calculation

Briefly, 1 MET equates to an individual's resting energy expenditure. According to the IPAQ scoring protocol, 3.3 METS is considered equivalent to walking, and moderate and vigorous activity to be 4 and 8 METS, respectively. To calculate the continuous variable of total MET minutes a week, the self-reported duration (minutes) and frequency (days) of each of these PA categories is multiplied by the by the specified metric.

For the purposes of regression analyses, employment status was re-categorised as per Table S1

Supplementary figures and tables

For peer review only

Figure S1: Timeline of lockdown restrictions in the United Kingdom in 2020

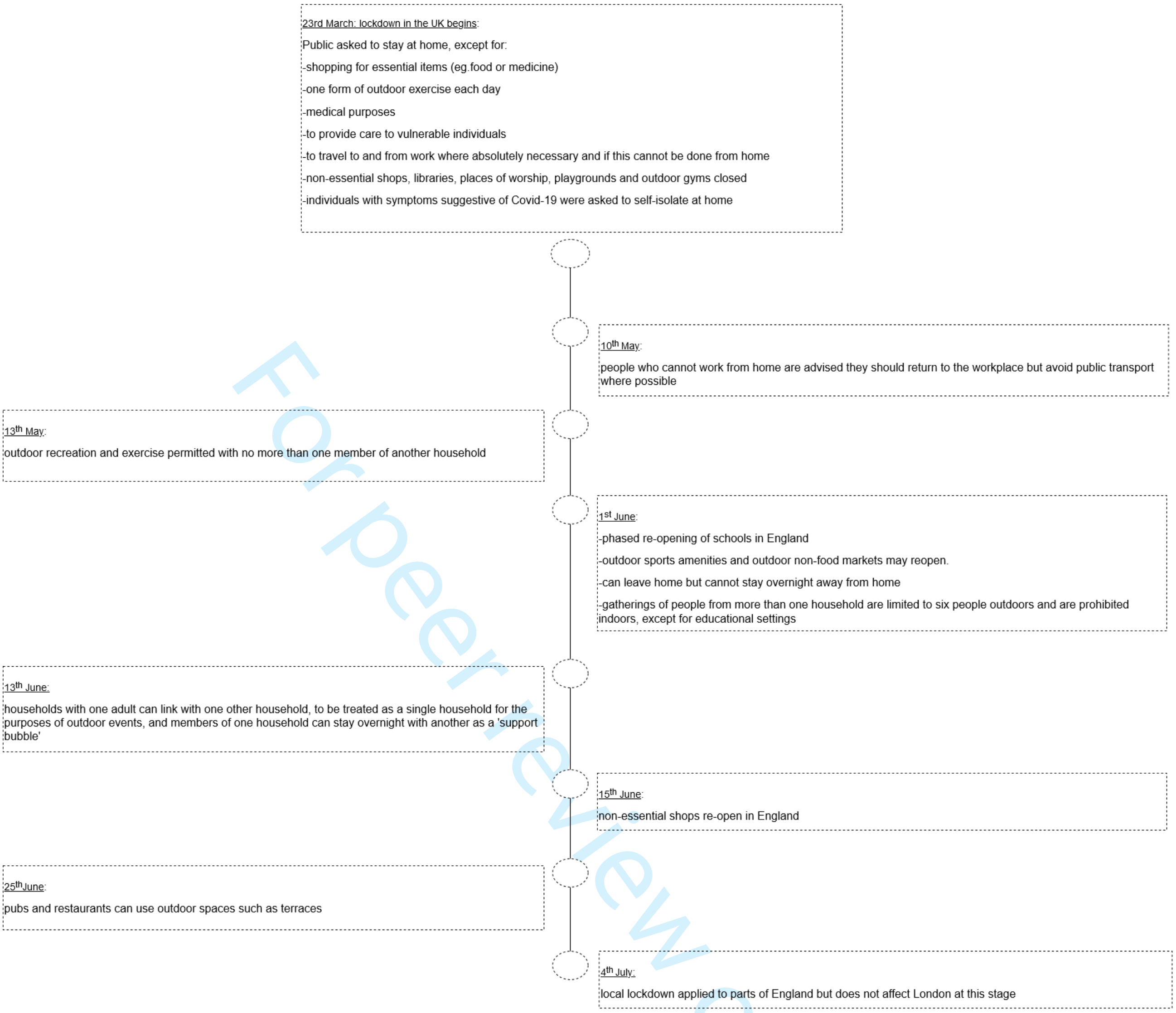

Gov.uk. UK Government COVID-19 guidance

For peer review only

Figure S2: Box-plot of distribution of MET minutes per week after introduction of lockdown by month of survey completion for 6,219 participants with completed IPAQ data

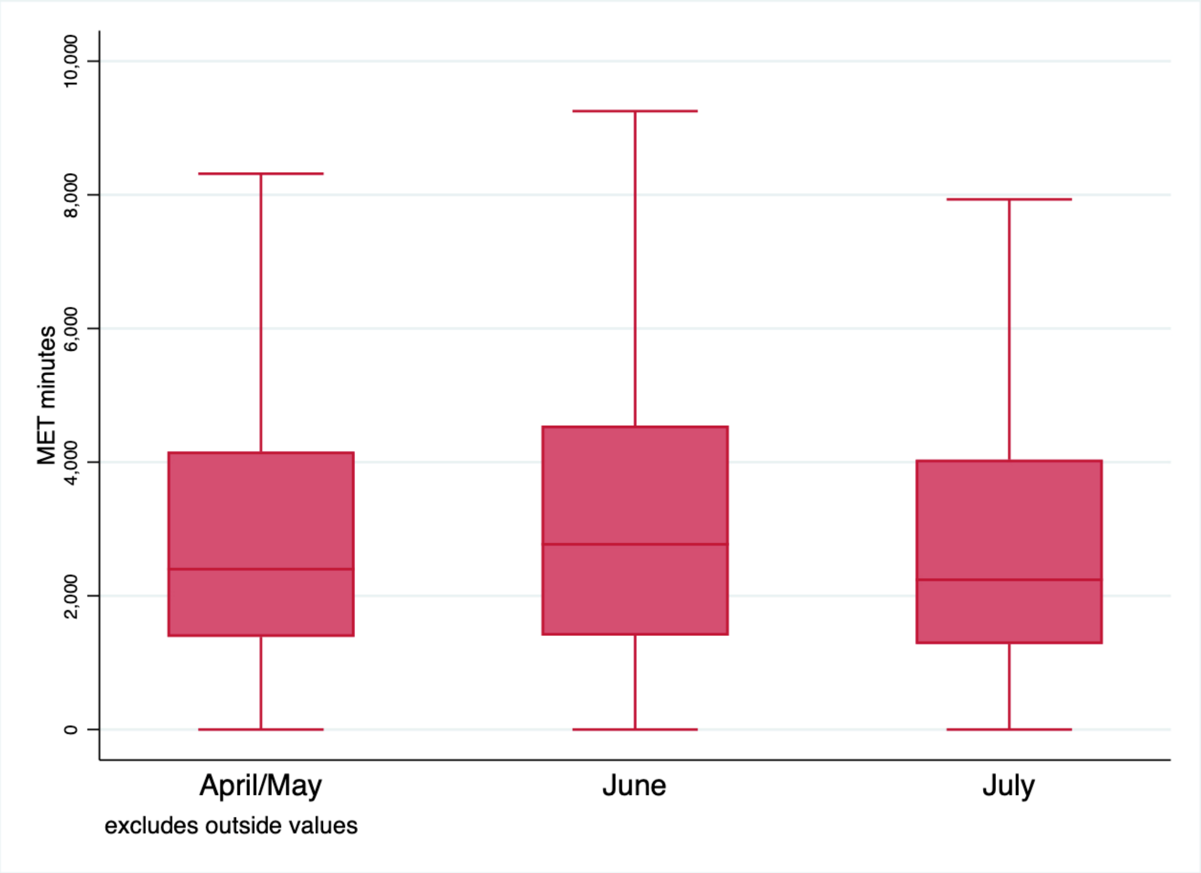

Table S1: Mean MET minutes after introduction of lockdown measures by month of survey completion

Month	Total	Percent	Mean MET minutes	p value¶
April/May*	4975	80.0%	3139	0.0007
June	994	16.0%	3470	
July	250	4.0%	2967	

* April (110) and May (4865) combined due to small numbers completed in April
¶ p-value from linear regression models of MET minutes as dependent variable, against survey completion month as explanatory variable.

For peer review only - http://bmjopen.bmj.com/site/about/guidelines.xhtml

Table S2: Re-categorisation of employment status

Recategorised variable	Original variable(s)
Retired	Retired
Employed	A key worker<br>A student<br>Continuing to work in your usual job; at home<br>Continuing to work in your usual job and leave home for your job
Furloughed	Furloughed
Unemployed	Had to close your business due to COVID-19<br>Lost my job due to the lockdown<br>Unemployed
Missing	None of the above

For peer review only

Figure S3: Causal diagram representing factors impacting on change in physical activity after lockdown

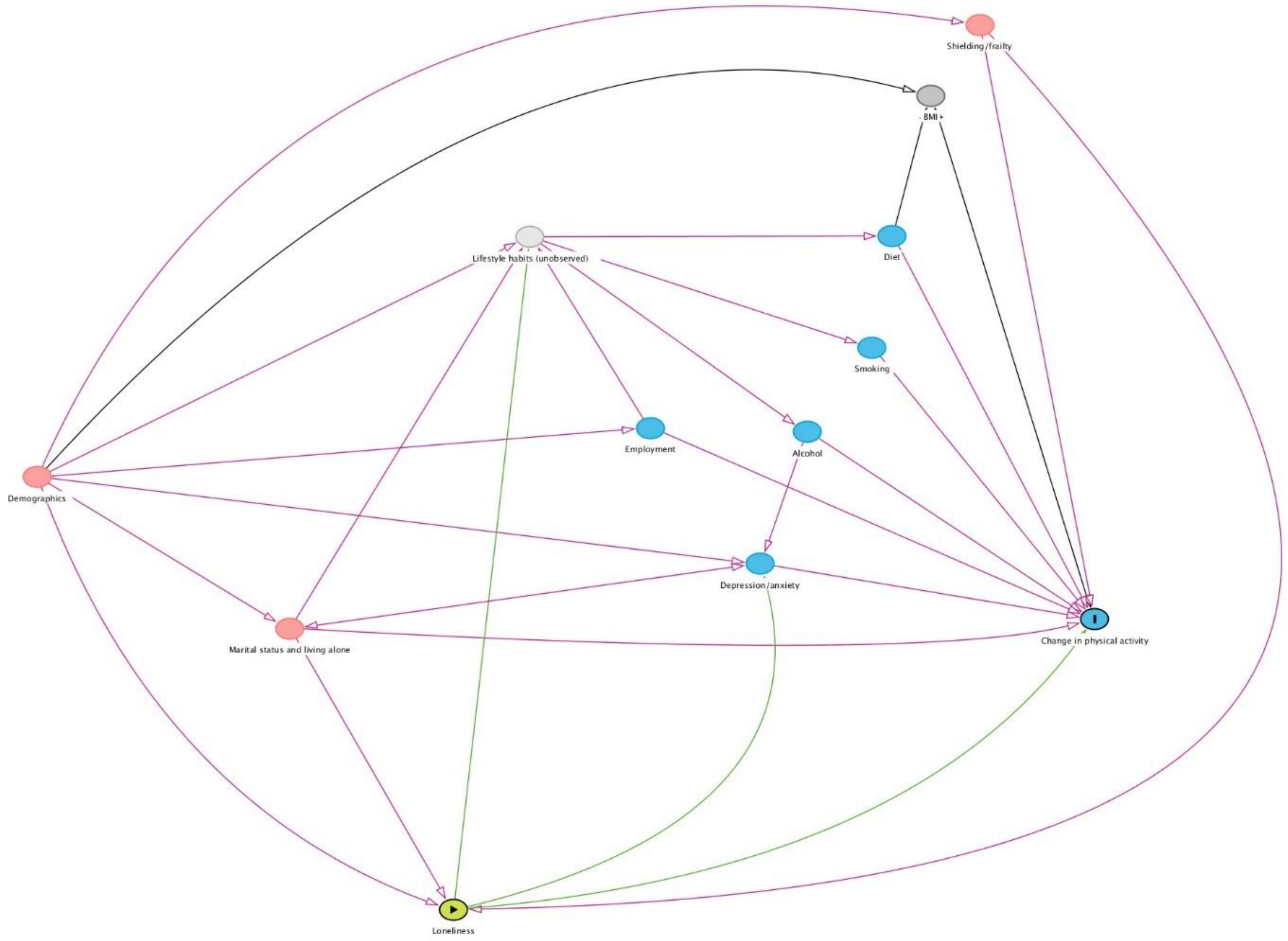

Figure S4: Box-plot of distribution of MET minutes per week before and after the introduction of lockdown for 6,219 participants with completed IPAQ data

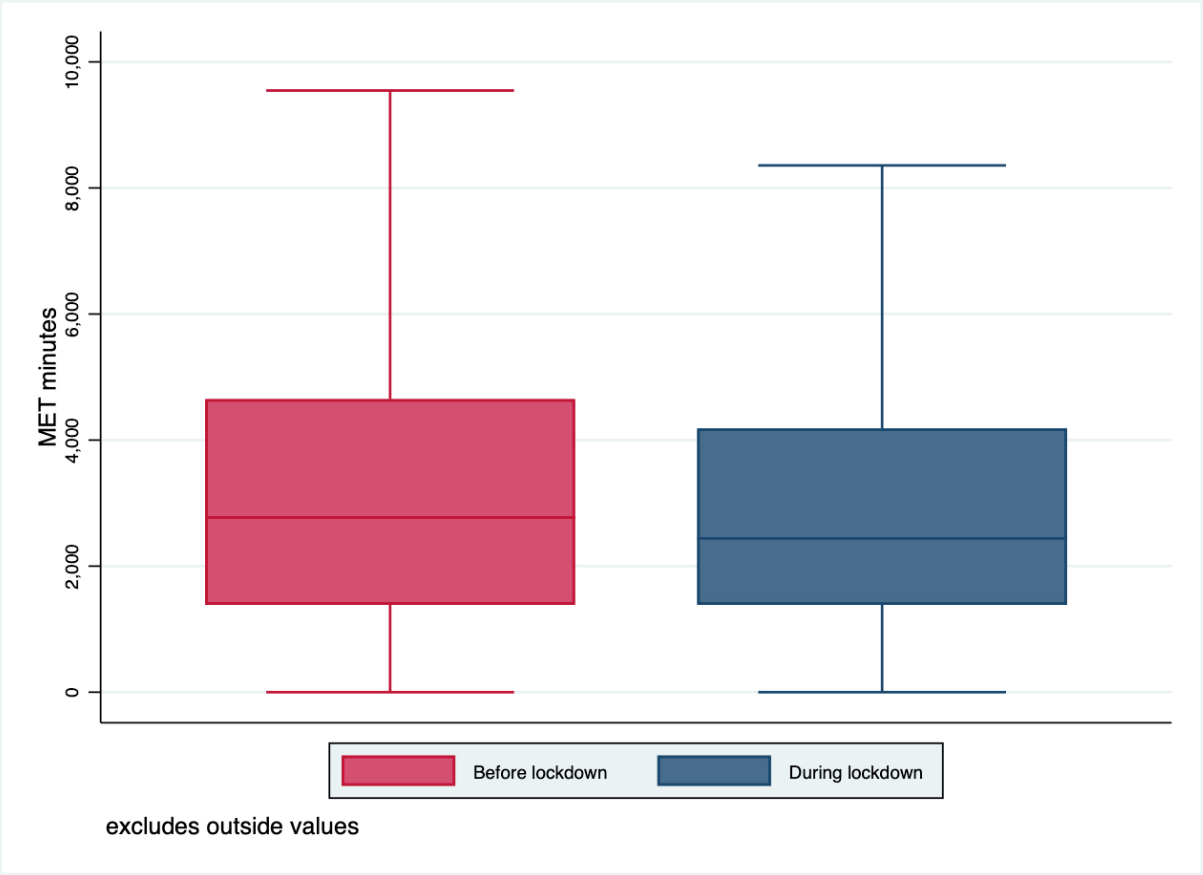

only

Figure S5: Box-plot of distribution of MET minutes per week before introduction of lockdown by month of survey completion for 6,219 participants with completed IPAQ data

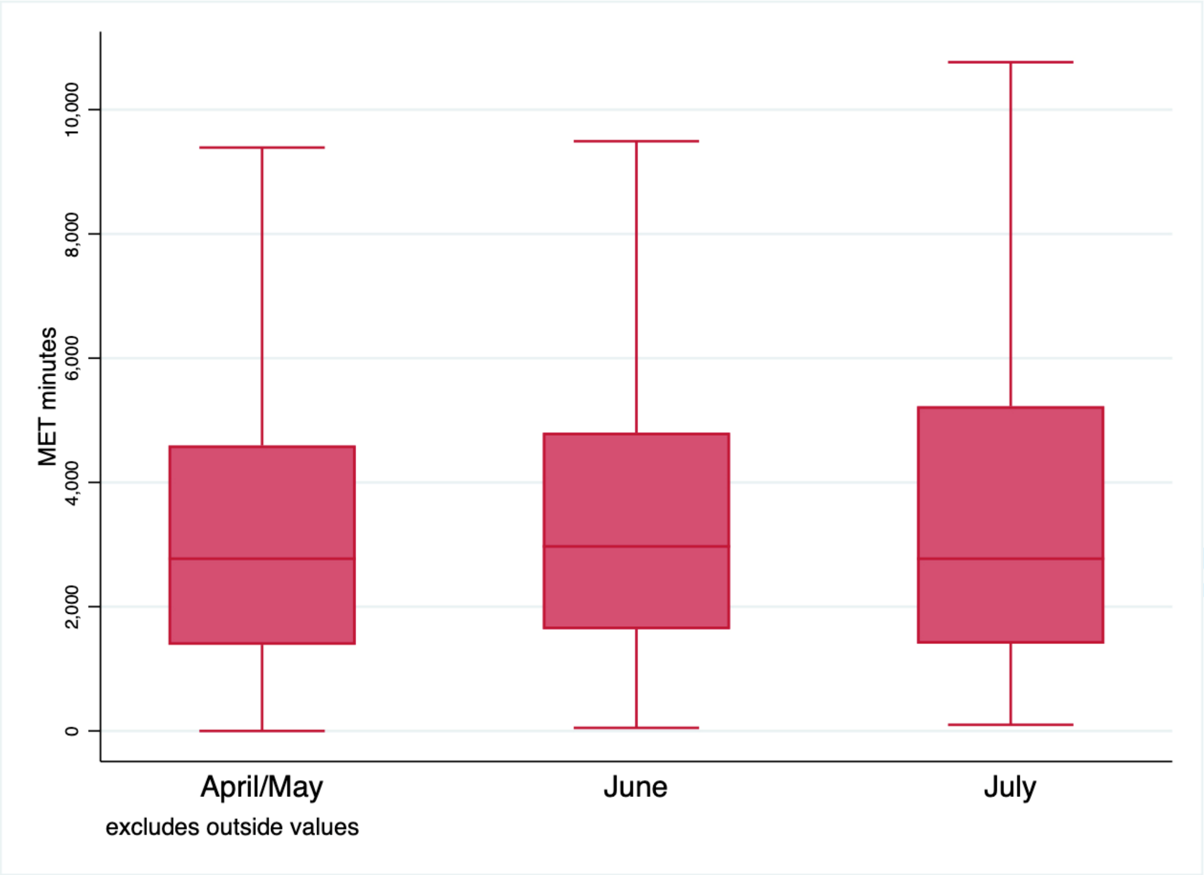

Linear regression models of MET minutes as dependent variable, against survey completion month as explanatory variable showed no significant association (p=0.1112).

Table S3: Unadjusted associations in physical activity (MET minutes per week) after introduction of lockdown measures and change from before lockdown, from linear regression models. Note: negative values for change in activity indicate reduction after lockdown

	Physical activity after lockdown (MET minutes/week)				Change in physical activity from before lockdown (MET minutes/week)			
Predictor	Mean	95% confidence interval		p value	Mean	95% confidence interval		p value
		Lower	Upper			Lower	Upper	
**Mean (whole cohort)**	3186	3120	3253	-	-333	-396	-271	-
**Age (years)**								
50-64	3341	3191	3491	<0.001	-196	-338	-55	0.184
65-74	3201	3112	3290		-362	-446	-278	
75-84	3092	2952	3232		-365	-497	-233	
85+	2326	1863	2790		-503	-940	-66	
**Sex**								
Female	3227	3138	3317	0.180	-450	-533	-366	<0.001
Male	3136	3036	3235		-189	-282	-95	
**Ethnicity**								
White	3196	3127	3265	0.425	-330	-394	-265	0.641
Mixed/multiple ethnic groups	3346	2819	3873		-392	-887	102	
Asian/Asian British	2929	2530	3327		-326	-700	48	
Black/African/Caribbean/Black British	3351	2551	4151		4	-746	754	
Other ethnic group	2754	2099	3410		-740	-1355	-125	
**Body Mass Index category**								
Underweight	3815	3137	4493	<0.001	-21	-649	607	0.055
Healthy weight	3569	3439	3700		-126	-247	-5	
Overweight	3130	2959	3300		-363	-521	-204	
Obese	2590	2309	2870		-400	-659	-140	

For peer review only

**Employment status**								
Employed	3093	2950	3236		-217	-351	-84	
Furloughed	3496	3122	3870	0.118	-488	-838	-139	0.101
Unemployed	3463	3031	3894		-672	-1076	-268	
Retired	3191	3111	3271		-334	-409	-259	
**Marital status**								
Divorced/single/widowed	3026	2908	3143	0.001	-540	-650	-430	<0.001
Living with a partner/married	3262	3181	3342		-236	-312	-161	
**Household**								
Not living alone	3262	3185	3340	<0.001	-240	-313	-168	<0.001
Living alone	2983	2855	3110		-582	-702	-463	
**Loneliness**								
Not ever	3284	3188	3380		-216	-306	-126	
Rarely	3087	2951	3224	0.024	-360	-488	-232	<0.001
Sometimes	3155	3010	3300		-481	-617	-345	
Often	2938	2666	3210		-762	-1018	-507	
**Shielding**								
Not shielding	3273	3196	3350	<0.001	-243	-315	-171	<0.001
Shielding	2942	2812	3072		-588	-710	-466	
**Frailty**								
Robust	3257	3183	3330	<0.001	-335	-404	-265	0.389
Pre-frail	2903	2746	3059		-328	-475	-180	
Frail	1952	1055	2849		-925	-1768	-82	
**Alcohol drinker**								
No	3044	2884	3203	0.054	-485	-634	-335	0.029
Yes	3217	3143	3290		-301	-370	-233	
**Smoker**								
No	3202	3135	3270	0.010	-322	-385	-259	0.046
Yes	2696	2318	3074		-689	-1043	-334	

For peer review only

**Diet**								
No change from usual - already had a healthy diet	3257	3183	3331	<0.001	-333	-402	-263	<0.001
My diet has become more healthy	3314	3119	3509		-55	-238	129	
My diet was healthy before but has got worse since lockdown	2523	2227	2818		-890	-1168	-612	
No change from usual - my diet isn't very healthy	2009	1641	2377		-479	-825	-133	
**HADS (depression score)**								
Normal (0-7)	3195	3119	3270	<0.001	-293	-365	-222	<0.001
Borderline (8-10)	2787	2495	3079		-676	-953	-399	
Abnormal (11-21)	2450	1971	2929		-1450	-1904	-997	
**HADS (anxiety score)**								
Normal (0-7)	3123	3044	3201	0.150	-312	-386	-237	0.004
Borderline (8-10)	3343	3109	3577		-348	-570	-125	
Abnormal (11-21)	3288	2971	3605		-836	-1137	-535	

*HADS – Hospital Anxiety and Depression Score

For peer review only

Table S4: Results of multivariable linear regression models of physical activity after lockdown, adjusted for age, sex, ethnicity, month of survey completion and baseline physical activity. Data presented as mean MET minutes/week +/- 95% confidence interval compared to the reference group, with negative values indicating lower physical activity than the reference.

Predictor	Physical activity after lockdown (MET minutes/week)	95% confidence interval		p value	Number of observations
		Lower	Upper		
**Age (years)**					
50-64 (reference)	-	-	-		
65-74	-154	-296	-12	<0.001	6155
75-84	-213	-380	-46		
85+	-640	-1034	-246		
**Sex**					
Female (reference)	-	-	-	0.053	6155
Male	108	-1	216		
**Ethnicity**					
White (reference)	-	-	-		
Mixed/multiple ethnic groups	-14	-442	415	0.517	6155
Asian/Asian British	-136	-463	191		
Black/African/Caribbean/Black British	248	-398	894		
Other ethnic group	-435	-969	100		
**Body Mass Index category**					
Underweight	153	-411	717		
Healthy weight (reference)	-	-	-	0.030	2987
Overweight	-341	-518	-165		
Obese	-578	-832	-324		
**Employment status**					
Employed (reference)	-	-	-	0.905	5958
Furloughed	47	-278	372		
Unemployed	-110	-480	259		

For peer review only

Retired	99	-48	246		
**Marital status**					
Divorced/single/widowed (reference)	-	-	-	<0.001	6155
Living with a partner/married	240	120	360		
**Household**					
Not living alone (reference)	-	-	-	<0.001	6155
Living alone	-277	-402	-152		
**Loneliness**					
Not ever (reference)	-	-	-		
Rarely	-161	-297	-25	<0.001	6077
Sometimes	-186	-329	-42		
Often	-452	-688	-217		
**Shielding**					
Not shielding (reference)	-	-	-	<0.001	6155
Shielding	-290	-417	-163		
**Frailty**					
Robust (reference)	-	-	-		
Pre-frail	-160	-301	-19	0.005	6142
Frail	-926	-1663	-189		
**Alcohol drinker**					
No (reference)	-	-	-	0.049	6155
Yes	145	1	289		
**Smoker**					
No (reference)				0.005	6155
Yes	-451	-762	-140		
**Diet**					
No change from usual - already had a healthy diet (reference)	-	-	-		
My diet has become more healthy	156	-13	326	<0.001	6155
My diet was healthy before but has got worse since lockdown	-662	-910	-414		

For peer review only

No change from usual - my diet isn't very healthy	-667	-975	-359		
**HADS (depression score)**					
Normal (0-7) (reference)	-	-	-	<0.001	5038
Borderline (8-10)	-408	-654	-163		
Abnormal (11-21)	-1007	-1401	-612		
**HADS (anxiety score)**					
Normal (0-7) (reference)	-	-	-	0.478	5038
Borderline (8-10)	94	-109	296		
Abnormal (11-21)	-220	-486	47		

HADS – Hospital Anxiety and Depression Score

For peer review only

Table S5: Multivariable linear regression model for physical activity after lockdown with loneliness, adjusted for age, sex, ethnicity, month of survey completion, baseline physical activity, living alone, marital status, shielding and frailty

Predictor	Physical activity after lockdown (MET minutes/week)	95% confidence interval		p value	Number of observations
		Lower	Upper		
**Loneliness**					
Not ever (reference)	-	-	-		
Rarely	-127	-265	11	0.007	6077
Sometimes	-107	-256	42		
Often	-306	-552	-60		

Adjusted: age, sex, ethnicity, month of survey completion, baseline physical activity, living alone, marital status, shielding, frailty

For peer review only

CHARIOT COVID-19 Rapid Response (CCRR) Study

Baseline Survey

Please answer all the questions in this survey before submitting it. Follow the prompts for those questions that are not applicable to you.

Symptoms

Q1. In the last week, have you had a cough?

<1> No

<2> Yes

Q2. In the last week, have you experienced unusual shortness of breath (difficulty breathing) compared to what's normal for you?

<1> No

<2> Yes, but it did not affect my normal activities

<3> Yes, it did affect my normal activities (eg walking short distances)

<4> Yes, even when I was sitting or lying down

Q3. In the last week, have you had a fever (feeling too hot) and did you take your temperature?

<1> I have NOT felt feverish

<2> I have felt feverish but did not check my temperature

<3> I felt feverish and my temperature was equal to, or BELOW 38 degrees Celcius

<4> I felt feverish and my temperature measured ABOVE 38 degrees Celcius

Q4. In the last week, have you experienced any of these other symptoms? Please do NOT include symptoms you experience on a regular basis due to a health condition you already know about. Please tick all that apply:

<1> Loss of sense of smell

<2> Loss of sense of taste

<3> Decrease in appetite (skipping meals)

<4> Diarrhoea

<5> Nauseas and/or Vomiting

<6> Abdominal pain/tummy ache

<7> Chills (feeling too cold)

<8> Difficulty sleeping

For peer review only

<9> Felt more tired than normal

<10> Severe Fatigue

<11> Sneezing

<12> Chest pain / tightness

<13> Tightness in chest

<14> Sore throat

<15> Hoarse voice

<16> Runny nose

<17> Blocked nose

<18> Sore eyes

<19> Itchy eyes

<20> Headache

<21> Joint pain / aches

<22> Dizziness

<23> Muscle pain/aches

<99> None of these

If you answered, 'None of these', please skip Q5 and go to Q6.

Q5. Thinking about the 14 days before your symptoms started, had you been in physical contact (within 2 metres / 6 feet) with someone who has a confirmed diagnosis of coronavirus (Covid-19), or someone with the following symptoms: dry cough, fever, loss of sense of smell, loss of sense of taste, shortness of breath or difficulty breathing.

<1> Yes, and it was an individual within my household

<2> Yes, and it was an individual from outside my household

<3> No, not that I am aware of

QX Since COVID-19 emerged in January, but before the official lockdown started on March 23rd 2020, which, if any of the following, have you experienced? Please do NOT include symptoms you experience on a regular basis due to a health condition you already know about. Please tick all that apply.

<1> New, continuous cough (coughing a lot for more than an hour, or have had 3 coughing episodes in 24 hours)

<2> High temperature (hot to touch on chest or back)

<3> Loss of sense of smell

<4> Loss of sense of taste

<5> Loss of appetite (skipping meals)

<6> Diarrhoea

<7> Vomiting

<8> Fatigue

<9> Sneezing

<10> Chest pain / tightness

For peer review only

<11> Sore throat

<12> Runny nose

<13> Itchy eyes

<14> Headache

<15> Joint pain / aches

<16> Muscle or joint pain

<99> None of these

If you answered, 'None of these', go to Q6.

QXa Approximately when did you start experiencing these symptoms?

[DD/MM/YYYY]

QXb Approximately how long did these symptoms last?

[Days:]

QXX Thinking about the 14 days before your symptoms started, had you been in physical contact (within 2 metres / 6 feet) with someone who has a confirmed diagnosis of coronavirus (Covid-19), or someone with the following symptoms: dry cough, fever, loss of sense of smell, loss of sense of taste, shortness of breath or difficulty breathing.

<1> Yes, and it was an individual within my household

<2> Yes, and it was an individual from outside my household

<3> No, not that I am aware of

Q6 Now, thinking about the period prior to last week, but after the official lockdown started on 23rd March 2020 , which, if any of the following, have you experienced? Please do NOT include symptoms you experience on a regular basis due to a health condition you already know about. Please tick all that apply.

<1> Fever (feeling too hot)

<2> New persistent cough

<3> Shortness of breath affecting normal activities

<4> Loss of sense of smell

<5> Loss of sense of taste

<6> Decrease in appetite (skipping meals)

<7> Diarrhoea

<8> Nauseas and/or vomiting

<9> Abdominal pain/tummy ache

<10> Chills (feeling too cold)

<11> Difficulty sleeping

<12> Felt more tired than normal

<13> Severe fatigue

For peer review only

<14> Sneezing

<15> Chest pain

<16> Tightness in chest

<17> Sore throat

<18> Hoarse throat

<19> Runny nose

<20> Blocked nose

<21> Sore eyes

<22> Itchy eyes

<23> Headache

<24> Dizziness

<25> Joint pain / aches

<26> Muscle pain/aches

If you answered, 'None of these', go to Q8.

Q6a Approximately when did you start experiencing these symptoms?

[DD/MM/YYYY]

Q6b Approximately how long did these symptoms last?

[Days:]

Q7 Thinking about the 14 days before your symptoms started, had you been in physical contact (within 2 metres / 6 feet) with someone who has a confirmed diagnosis of coronavirus (Covid-19), or someone with the following symptoms: dry cough, fever, loss of sense of smell, loss of sense of taste, shortness of breath or difficulty breathing.

<1> Yes, and it was an individual within my household

<2> Yes, and it was an individual from outside my household

<3> No, not that I am aware of

Q8 Have you or anyone in your house been tested for coronavirus? Please tick all that apply

<1> No testing

<2> I have not been tested -- BUT I think I have already had coronavirus and recovered

<3> I was tested - positive result

<4> I was tested - awaiting result

<5> I was tested - negative result

<6> Household member tested - positive result

<7> Household member tested - awaiting result

For peer review only

<8> Household member tested - negative result

Q9 In the last week, has anyone in your household had a new cough or fever?

Not applicable

<1> No

<2> Yes

Q10 Have you had any healthcare contact since the lockdown started? Please tick all that apply

<1> No

<2> Yes - remote appointment with my GP (phone/video)

<3> Yes - I attended my GP practice for an appointment

<4> Yes - remote appointment with hospital (phone/video)

<5> Yes - I attended hospital for an appointment

<6> Yes - attended Accident and Emergency

<7> Yes -- I was admitted to hospital (not because of coronavirus)

<8> Yes -- I was admitted to hospital with symptoms of coronavirus

<9> Yes – One or more remote calls to 111- home visit by ambulance

Q11. In the last week, have you been taking any medication for new symptoms?

<1> No

<2> Yes

<3> If yes, what medication?

Underlying conditions

For the following question, please remember that your answers are always treated confidentially and are never analysed individually. We have provided you with a "Prefer not to say" option if you would rather not share your experiences.

Q12 Which, if any, of the following chronic health conditions have you been diagnosed with? (Please select all that apply. If you do not currently have a chronic health condition, please select the 'None of these' option)

<1> Arthritis

<2> Asthma

For peer review only

<3> My doctor has told me I have severe asthma

<4> I am having cancer treatment

<5> Blood or bone marrow cancer, such as leukaemia

<6> Cystic fibrosis

<7> Chronic obstructive pulmonary disease (COPD)

<8> Diabetes

<9> Epilepsy

<10> Heart disease

<11> High blood pressure

<12> High cholesterol

<13> HIV/ AIDS

<14> Mental health condition

<15> Multiple Sclerosis

<16> I have had an organ transplant

<17> I have a condition that makes me much more likely to get infections

<18> I am taking medicine that weakens my immune system

<19> Dementia, Parkinson's or other neurological disease

<98> Prefer not to say

<99> None of these

Contacts

[Q13 What is your date of birth:

Date …..]

Q14 What is your sex:

<1> Female

<2> Male

<3> Prefer not to say

Q15 What ethnic group best describes you? Please select one option only.

<1>

English / Welsh / Scottish / Northern Irish / British

<11>

Bangladeshi

For peer review only

<2>

Irish

<12>

Chinese

<3>

Gypsy or Irish Traveller

<13>

Any other Asian background

<4>

Any other White background

<14>

African

<5>

White and Black Caribbean

<15>

Caribbean

<6>

White and Black African

<16>

Any other Black / African / Caribbean background

<7>

White and Asian

<17>

Arab

<8>

Any other Mixed / Multiple ethnic background

<18 fixed>

Any other ethnic group

<9>

Indian

<19 fixed>

Prefer not to say

For peer review only

<10>

Pakistani

Q16 Who else is CURRENTLY living in your household? Please tick all that apply

<1> I live by myself

<2> I live with my partner

<3> I live with my child/children aged under 18

<4> I live with my child/children aged over 18

<5> I live with family members other than partner / children

<6> I live with housemates

16a Where are you living during lockdown?

<1> My usual home

<2> not my usual home – keeping away from household members who are at high risk coronavirus

<3> Not my usual home – other reason

Q17 How many people, including yourself, are there in your household? Please include both adults and children. If you live alone, enter 1

Number

Q17a

For each household member ask age (in years) and sex

The following questions will ask you to report on how many people you have come into contact with both inside and outside of your household.

A contact is defined as either:

• Direct skin-to-skin physical contact (e.g. kiss/embrace/handshake)

• Face-to-face conversation with another person which lasts over 3 mins, within 2m distance

• Being within 2m distance from another individual for over 5 mins

Note: if you contacted the same person in different times through the day, they should be counted once.

Q18 How many different people did you have contact with, both inside your household and while outside (after having left your household) in the past 7 days?

For peer review only

Enter 0 if you had no contacts in the last 7 days

1. (enter number)

2. Don't know

Q19a Among the contacts you had, just from yesterday, both inside your household and while outside (after having left your household), how many belonged to the following age groups?

No contacts yesterday

0 to <10 years old (enter number)

10 to <20 years old (enter number)

20 to <30 years old (enter number)

30 to <40 years old (enter number)

40 to <50 years old (enter number)

50 to <60 years old (enter number)

60 to <70 years old (enter number)

70 to <80 years old (enter number)

80 to <90 years old (enter number)

90+ years old (enter number)

12. Don't know

IF Q18 is NOT=0

Q19b How many different people did you come in contact with in the past 7 days outside of your household?

Enter 0 if you had no contacts in the last 7 days outside of your household

1. (enter number)

For peer review only

2. Don't know

IF Q19b is NOT=0

Q19c Among the contacts you had, just from yesterday, outside your household, how many belonged to the following age groups?

No contacts yesterday

0 to <10 years old (enter number)

10 to <20 years old (enter number)

20 to <30 years old (enter number)

30 to <40 years old (enter number)

40 to <50 years old (enter number)

50 to <60 years old (enter number)

60 to <70 years old (enter number)

70 to <80 years old (enter number)

80 to <90 years old (enter number)

90+ years old (enter number)

Don't know

IF Q19b is NOT=0

Q19c Among the contacts that you have had in the past 7 days outside your household, how many contacts occurred at work?

Enter 0 if you had no contacts in the last 7 days outside of your household that occurred at work

For peer review only

1. (enter number)

2. Don't know

For the following questions please answer according to the following terms;

Self-isolation – refers to those who are symptomatic and self-isolating for 7 days from when symptoms started

Shielding – those in specific vulnerable groups staying at home for 12 weeks. These groups would include those with underlying chronic health conditions: cancers, respiratory disease, on immunosuppressants, those at increased risk of infection or pregnant women with heart disease and/or those advised by the NHS of their extremely vulnerable status'.

Household quarantine – 14-day quarantine period for all members of a household from the first day of symptom onset in first case in that household

Social distancing and isolation

Q20 Are you currently in self-isolation?

<1> Yes

<2> No

If yes, for how long:…days

Q21 Are you currently shielding as per government guidelines for vulnerable groups?

<1> Yes

<2> No

Q22 Have you moved residence recently due to the pandemic? Y/N

Q23. Are you single, married, living with a partner, divorced, widowed?

Q24. Are you

<1> Continuing to work in your usual job; at home

<2> Continuing to work in your usual job and leave home for your job <3> volunteering in response to the COVID pandemic

<4> a key worker

<5> unemployed

<6> retired

<7> furloughed (put on leave, still getting paid)

<8> had to close your business due to COVID-19

<9> lost my job due to the lockdown

<10> a student

<99> None of the above

Q25. How often are you now contacting friends/family members remotely (Skype/Zoom/Mobile/landline phone etc)?

Several times per day, once a day, 2-3 x per week, 4-6 x per week, once a week, less than once a week?

For peer review only

Q26 Overall, how are your relationships with other members of your household?

Not applicable

1 = worst

2

3

4

5

6

7

8

9

10 = best

Q27 If you are leaving your home, what activity is this for? Please tick all that apply

<1> I am not leaving my home

<2> Commute to work

<3> Essential shopping

<4> Exercise

<5> Other

Q28 Have you or anyone in your household received a letter or message informing you that you are in the population at 'high risk' from coronavirus? Please tick all that apply.

<1> No - Neither myself or anyone in my household is at 'high risk'

<2> No - but I think I should have

<3> No - but someone in my household is at 'high risk'

<4> Yes - letter about me

<5> Yes - letter about someone in my household

Health behaviours: dietary, alcohol and smoking

Q29 Do you drink alcohol?

<1> Yes (If yes trigger sub-questions)

Drinking less since lockdown

Drinking the same amount since lockdown

Drinking more since lockdown

How many units do you consume per week:…units

For peer review only

(half pint/ 300ml = approx. 1 unit, 175ml glass wine= approx. 2 units)

<2> No (If no, trigger sub-questions)

I never drink alcohol

I had already stopped drinking alcohol before lockdown

I stopped drinking alcohol when lockdown started

Q30 Do you smoke?

<1> Yes (if yes, trigger sub-questions)

Smoking less since lockdown

Smoking the same amount since lockdown

Smoking more since lockdown

If yes, how many cigarettes or roll-ups do you smoke per day:…

<2> No (if now, trigger sub-questions)

I never smoked

I had already stopped smoking before lockdown

I stopped smoking since the lockdown

Q30a) Has there been a change in your vaping (e-cigarettes) status since the coronavirus lockdown?

<1> I never vaped

<2> I had already stopped vaping before

<3> I stopped vaping since the lockdown

<4> Vaping less

<5> Vaping the same amount

<6> Vaping more

Q31 Since the lockdown, are you managing to keep a healthy diet, for example, eating fresh fruits and vegetables?

<1> No change from usual - already had a healthy diet

<2> No change from usual - my diet isn't very healthy

<3> My diet has become more healthy

<4> My diet was healthy before but has got worse since lockdown

Q32 On average, how many portions (or servings) of fruit and vegetables do you eat per day?.......

For peer review only

- One portion is typically 80g, 3 heaped tablespoons of cooked veg or 1 cereal bowl of mixed salad
- Three heaped tablespoons of beans and other pulse vegetables, such as kidney beans, lentils and chickpeas, count as 1 portion.
- The following starchy vegetables should not be included – potatoes, yams, cassava and plantain

Q32a Have you ever skipped meals due to difficulties accessing food as a result of COVID-19?

Yes /No

If yes:

How many meals per week, on average have you missed?

<1> 1-3 meals per week

<2> 4-6 meals per week

<4> 7-9 meals per week

<5> 10 or more meals per week

Biometric data: height and weight

Q33 Please enter your weight: Kg

Q34 Please enter your height:…cm

Q35 Do you have a recent (from the past week) blood pressure?_____mm/Hg

Current Physical activity: International Physical Activity Questionnaire

We are interested in finding out about the kinds of physical activities that people do as part of their everyday lives. The questions will ask you about the time you spent being physically active in the last 7 days. Please answer each question even if you do not consider yourself to be an active person. Please think about the activities you do at work, as part of your house and garden work, to get from place to place, and in your spare time for recreation, exercise or sport.

Think about all the vigorous activities that you did in the last 7 days. Vigorous physical activities refer to activities that take hard physical effort and make you breathe much harder than normal. Think only about those physical activities that you did for at least 10 minutes at a time.

Q36: During the last 7 days, on how many days did you do vigorous physical activities like heavy lifting, digging, aerobics, or fast bicycling?

_____ days per week

If no vigorous physical activities, skip to question 38

Q37: How much time did you usually spend doing vigorous physical activities on one of those days? If you only exercised in hours or minutes, please input a '0' in the non-applicable field.

_____ hours per day

For peer review only

_____ *minutes per day*

Think about all the moderate activities that you did in the last 7 days. Moderate activities refer to activities that take moderate physical effort and make you breathe somewhat harder than normal. Think only about those physical activities that you did for at least 10 minutes at a time.

Q38: During the last 7 days, on how many days did you do moderate physical activities like carrying light loads or bicycling at a regular pace? Do not include walking.

_____ *days per week*

If no moderate physical activities, skip to question 40

Q39: How much time did you usually spend doing moderate physical activities on one of those days? If you only exercised in hours or minutes, please input a '0' in the non-applicable field.

_____ *hours per day*

_____ *minutes per day*

Think about the time you spent walking in the last 7 days. This includes at work and at home, walking to travel from place to place, and any other walking that you have done solely for recreation, sport, exercise, or leisure.

Q40: During the last 7 days, on how many days did you walk for at least 10 minutes at a time?

_____ *days per week*

No walking, skip to question 42

Q41: How much time did you usually spend walking on one of those days? If you only exercised in hours or minutes, please input a '0' in the non-applicable field.

_____ *hours per day*

_____ *minutes per day*

The last question is about the time you spent sitting on weekdays during the last 7 days. Include time spent at work, at home, while doing course work and during leisure time. This may include time spent sitting at a desk, reading, or sitting or lying down to watch television.

Q42: During the last 7 days, how much time did you spend sitting on a week day?

For peer review only

If you only exercised in hours or minutes, please input a '0' in the non-applicable field.

_____ *hours per day*

_____ *minutes per day*

Previous Physical activity: International Physical Activity Questionnaire

These questions will ask you about the time you spent being physically active in the 7 days prior to implementation of social distancing measures (please use first week of March 2020). Please answer each question even if you do not consider yourself to be an active person. Please think about the activities you do at work, as part of your house and garden work, to get from place to place, and in your spare time for recreation, exercise or sport.

Think about all the vigorous activities that you did in the 7 days prior to social distancing measures. Vigorous physical activities refer to activities that take hard physical effort and make you breathe much harder than normal. Think only about those physical activities that you did for at least 10 minutes at a time.

Q43: During the 7 days prior to social distancing measures (please use first week of March 2020), on how many days did you do vigorous physical activities like heavy lifting, digging, aerobics, or fast bicycling?

_____ *days per week*

If no vigorous physical activities, skip to question 45

Q44: How much time did you usually spend doing vigorous physical activities on one of those days? If you only exercised in hours or minutes, please input a '0' in the non-applicable field.

_____ *hours per day*

_____ *minutes per day*

Think about all the moderate activities that you did in the 7 days prior to social distancing measures. Moderate activities refer to activities that take moderate physical effort and make you breathe somewhat harder than normal. Think only about those physical activities that you did for at least 10 minutes at a time.

Q45: During the 7 days prior to social distancing measures (please use first week of March 2020), on how many days did you do moderate physical activities like carrying light loads or bicycling at a regular pace? Do not include walking.

_____ *days per week*

If no moderate physical activities, skip to question 47

For peer review only

Q46: How much time did you usually spend doing moderate physical activities on one of those days? If you only exercised in hours or minutes, please input a '0' in the non-applicable field.

_____ *hours per day*

_____ *minutes per day*

Think about the time you spent walking in the 7 days prior to social distancing measures. This includes at work and at home, walking to travel from place to place, and any other walking that you have done solely for recreation, sport, exercise, or leisure.

Q47: During the 7 days prior to social distancing measures (please use first week of March 2020), on how many days did you walk for at least 10 minutes at a time?

_____ *days per week*

No walking, skip to question 49

Q48: How much time did you usually spend walking on one of those days? If you only exercised in hours or minutes, please input a '0' in the non-applicable field.

_____ *hours per day*

_____ *minutes per day*

The last question is about the time you spent sitting on weekdays during 7 days prior to social distancing measures. Include time spent at work, at home, while doing course work and during leisure time. This may include time spent sitting at a desk, reading, or sitting or lying down to watch television.

Q49: During the 7 days prior to social distancing measures (please use first week of March 2020), how much time did you spend sitting on a week day? If you only exercised in hours or minutes, please input a '0' in the non-applicable field.

_____ *hours per day*

_____ *minutes per day*

For peer review only

Frailty Questionnaire:

Q50: Are you fatigued?

<1> Yes

<2> No

Q51: Can you walk up one flight of stairs?

<1> Yes

<2> No

Q52: Can you walk around the block?

<1> Yes

<2> No

Q53: Do you have more than 5 illnesses?

<1> Yes

<2> No

Q54: Have you lost more than 5% of your weight in the past 6 months?

<1> Yes

<2> No

Qx Have you had a fall during the COVID lockdown period?

Yes/No

If yes:

What actions were taken (select multiple where applicable):

a) No follow-up required, I did not hurt myself

b) Pain medication

c) A hospital and/or GP appointment

d) A follow-up X-ray

e) Sling/plaster cast for a fracture

For peer review only

f) Surgery

Mood

a) Tick the box beside the reply that is closest to how you have been feeling in the past week.

Don't take too long over you replies: your immediate response is best.

Tick here

1.

Tick here

8.

I feel tense or 'wound up':

I feel as if I am slowed down:

Most of the time

Nearly all the time

A lot of the time

Very often

From time to time, occasionally

Sometimes

Not at all

For peer review only

Not at all

2.

9.

I still enjoy the things I used to

enjoy:

I get a sort of frightened feeling like 'butterflies' in the stomach:

Definitely as much

Not at all

Not quite so much

Occasionally

Only a little

Quite Often

Hardly at all

Very Often

3.

10.

I get a sort of frightened feeling as if something awful is about to

happen:

I have lost interest in my appearance:

For peer review only

Very definitely and quite badly

Definitely

Yes, but not too badly

I don't take as much care as I should

A little, but it doesn't worry me

I may not take quite as much care

Not at all

I take just as much care as ever

4.

11.

I can laugh and see the funny side of things:

I feel restless as I have to be on the move:

As much as I always could

Very much indeed

Not quite so much now

Quite a lot

Definitely not so much now

Not very much

For peer review only

Not at all

Not at all

5.

12.

Worrying thoughts go through my mind:

I look forward with enjoyment to things:

A great deal of the time

As much as I ever did

A lot of the time

Rather less than I used to

From time to time, but not too often

Definitely less than I used to

Only occasionally

Hardly at all

6.

13.

I feel cheerful:

For peer review only

I get sudden feelings of panic:

Not at all

Very often indeed

Not often

Quite often

Sometimes

Not very often

Most of the time

Not at all

7.

14.

I can sit at ease and feel relaxed:

I can enjoy a good book or radio or TV program:

Definitely

Often

Usually

Sometimes

Not Often

For peer review only

Not often

Not at all

Very seldom

Qx . I experience a general sense of emptiness

- *Not ever*
- *Rarely*
- *Sometimes*
- *Often*

Qy. There are plenty of people I can rely on when I have problems

- *Not ever*
- *Rarely*
- *Sometimes*
- *Often*

Qz. I miss having people around me

- *Not ever*
- *Rarely*
- *Sometimes*
- *Often*

Please check you have answered all the questions above.

b) For each of the 17 mood questions above, please also indicate if you are feeling or experiencing this 1, less than; 2, the same as; or 3, more than before social isolation was implemented.

<1> [1 or 2 or 3]

<2> [1 or 2 or 3]

<3> [1 or 2 or 3]

<4> [1 or 2 or 3]

<5> [1 or 2 or 3]

<6> [1 or 2 or 3]

<7> [1 or 2 or 3]

<8> [1 or 2 or 3]

<9> [1 or 2 or 3]

<10> [1 or 2 or 3]

For peer review only

<11> [1 or 2 or 3]

<12> [1 or 2 or 3]

<13> [1 or 2 or 3]

<14> [1 or 2 or 3]

<15> [1 or 2 or 3]

<16> [1 or 2 or 3]

<17> [1 or 2 or 3]

Q. People may have worries about Covid-19. Have you been worried about any of the following and, if so, how much?

Not at all

Little

Some

Rather much

Very much

Getting Covid-19 infection and/or infecting someone else

That a person close to me could get infected with Covid-19

Being discriminated against or avoided because of Covid-19

Impact of the Covid-19 epidemic on my own

For peer review only

economy and/or loss of my employment

Economic impact of the Covid-19 epidemic on the global economy

The government's and/or health system's lack of ability to handle the Covid-19 pandemic situation, including the shortage of food and other groceries

Imperial College Sleep Quality (ICSQ) Questionnaire

Instructions:

The following questions relate to your usual sleep habits for a period of one month before and during a period of reduced social contact. Your answers should indicate the most accurate reply for the majority of days and nights during these periods.

Please answer all questions.

1. During the period before reduced social contact, what time did you usually go to bed at night: bed-time was ………………………………………………….

1b) During the period of reduced social contact, what time have you usually gone to bed at night: bed-time is - ………………………………………………………......

2. During the period before reduced social contact, how long (in minutes) did it usually take you to fall asleep each night: number of minutes - …………….….

2b) During the period of reduced social contact, how long (in minutes) has it usually taken you to fall asleep each night: number of minutes - ……………………………

3. During the period before reduced social contact, what time did you usually get up in the morning: getting-up time was - ………………………………………

3b) During the period of reduced social contact, what time do you usually get up in the morning: getting-up time is - …………………………………………………………..

4. During the period before reduced social contact, how many hours of actual sleep did you get at night? (This may be different from the number of hours you

For peer review only

spent in bed): hours of sleep per night - …………………………………………

4b) During the period of reduced social contact, how many hours of actual sleep do you get at night? (This may be different from the number of hours you spend in bed): hours of sleep per night - ………………………………………………....

5. During the period before reduced social contact, how often did you have trouble sleeping because you could not get to sleep within 30 minutes:

o Not ever

o Less than once a week

o Once or twice a week

o Three or more times a week

5b) During the period of reduced social contact, how often have you had trouble sleeping because you could not get to sleep within 30 minutes:

o Not ever

o Less than once a week

o Once or twice a week

o Three or more times a week

6. During the period before reduced social contact, did you experience poor sleep (restless and unable to sleep):

o Not ever

o Less than once a week

o Once or twice a week

o Three or more times a week

6b) During the period of reduced social contact, have you experienced poor sleep (restless and unable to sleep):

o Not ever

o Less than once a week

o Once or twice a week

o Three or more times a week

7a) During the period before reduced social contact, did you experience loneliness (felt isolated, with no companions):

o Not ever

o Rarely

o Sometimes

o Often

7b) During the period of reduced social contact, have you experienced loneliness (felt isolated, with no companions):

o Not ever

o Rarely

o Sometimes

o Often

7c) During the period of reduced social contact, have you experienced loneliness: 1,

For peer review only

less than; 2, the same as; or 3, more than before social isolation was implemented

Select: [1 or 2 or 3]

Functional Activities Questionnaire

For each of the tasks below please rate your ability to carry out the task/activity independently on the following scale:

1. I had no difficulty

2. I had some difficulty, but I completed the task/activity myself.

3. I need some assistance to complete the task/activity:

a) I did not need assistance prior to COVID-19 lockdown but need assistance now to maintain social isolation/distancing

b) I could do the task/activity before the COVID-19 lockdown, but now would need assistance even if it were not to maintain social distancing

c) I required assistance since before the COVID-19 lockdown

4. I needed others to do this for me,

a) I could do the task/activity myself or with assistance prior to COVID-19 lockdown but need others to do it for me to maintain social isolation/distancing

b) I could do the task/activity myself or with assistance before the COVID-19 lockdown, but now would need others to do it for me even if it were not to maintain social distancing

c) I required others to do it for me since before the COVID-19 lockdown

5. I am unsure if I require assistance (e.g., never did the task/activity or have not done the task/activity over the past week)

Activities:

1. Writing cheques, paying bills, balancing cheque book, using an ATM cash machine

Response: …………………

2. Assembling tax records, business affairs, or papers

Response: …………………

3. Shopping alone for household necessities, medicines or groceries

Response: …………………

4. Playing a game of skill, working on a hobby

Response: …………………

5. Heating water, making a cup of coffee, turning off stove after use

Response: …………………

For peer review only

6. Preparing a balanced meal

Response: …………………

7. Keeping track of current events

Response: …………………

8. Paying attention to, understanding, discussing TV, video, book, magazine

Response: …………………

9. Remembering appointments, family occasions, public holidays, to take medications

Response: …………………

10. Travelling out of my neighbourhood by taxi, car, bus or train and making travel arrangements.

Response: …………………

THANK YOU FOR COMPLETING THIS QUESTIONNAIRE. YOUR RESPONSES HAVE BEEN SAVED AND SENT TO THE STUDY TEAM.

NHS health advice and information regarding the novel coronavirus can be found here: https://www.nhs.uk/conditions/coronavirus-covid-19/

For Advice on Mental health we suggest using these links:

1. The NHS Every Mind Matters website has information on how to look after your mental wellbeing while in isolation: https://www.nhs.uk/oneyou/every-mind-matters/

2. The charity Mental Health UK have advice on managing mental health during the coronavirus outbreak: https://mentalhealth-uk.org/help-and-information/covid-19-and-your-mental-health/

3. The NHS recommends a range of mobile apps to help with mental wellbeing, many of which are free to download: https://www.nhs.uk/apps-library/category/mental-health/

4. If you need someone to talk to about your mental health, the charity Samaritans have a helpline available 24 hours a day, 7 days a week:

a. Call: 116 123

b. or visit: https://www.samaritans.org/how-we-can-help/contact-samaritan/

For Advice on Physical activity we suggest using these links:

1. The NHS Live Well website has a range of free advice and programmes from light activity to more strenuous exercises for those aged under 65: https://www.nhs.uk/live-well/exercise/

2. The NHS Live Well website has a range of free advice and programmes from light activity to more strenuous exercises for those aged 65 or older: https://www.nhs.uk/live-well/exercise/physical-activity-guidelines-older-adults/

3. Tips, advice and guidance from Sport England on how to keep or get active in and around your home: https://www.sportengland.org/stayinworkout

For peer review only

4. Stay Active at Home: a simple set of exercises designed for older people to stay active at home: https://www.csp.org.uk/public-patient/keeping-active-and-healthy/staying-healthy-you-age/staying-strong-you-age/strength

For Advice on Sleep we suggest using these links:

1. The NHS ten top tips to improve sleep: https://www.nhs.uk/live-well/sleep-and-tiredness/10-tips-to-beat-insomnia/

2. The NHS recommends a range of mobile apps to help with sleep: https://www.nhs.uk/apps-library/category/sleep/

Supplementary Table 1: CCRR survey

For peer review only

STROBE 2007 (v4) checklist of items to be included in reports of observational studies in epidemiology*
Checklist for cohort, case-control, and cross-sectional studies (combined)

Section/Topic	Item #	Recommendation	Reported on page #
**Title and abstract**	1	(*a*) Indicate the study's design with a commonly used term in the title or the abstract	1
		(*b*) Provide in the abstract an informative and balanced summary of what was done and what was found	4
**Introduction**			
Background/rationale	2	Explain the scientific background and rationale for the investigation being reported	5
Objectives	3	State specific objectives, including any pre-specified hypotheses	5
**Methods**			
Study design	4	Present key elements of study design early in the paper	5
Setting	5	Describe the setting, locations, and relevant dates, including periods of recruitment, exposure, follow-up, and data collection	5-6
Participants	6	(*a*) *Cohort study*—Give the eligibility criteria, and the sources and methods of selection of participants. Describe methods of follow-up<br>*Case-control study*—Give the eligibility criteria, and the sources and methods of case ascertainment and control selection. Give the rationale for the choice of cases and controls<br>*Cross-sectional study*—Give the eligibility criteria, and the sources and methods of selection of participants	5-6
		(*b*) *Cohort study*—For matched studies, give matching criteria and number of exposed and unexposed<br>*Case-control study*—For matched studies, give matching criteria and the number of controls per case	
Variables	7	Clearly define all outcomes, exposures, predictors, potential confounders, and effect modifiers. Give diagnostic criteria, if applicable	6
Data sources/ measurement	8*	For each variable of interest, give sources of data and details of methods of assessment (measurement). Describe comparability of assessment methods if there is more than one group	6-7
Bias	9	Describe any efforts to address potential sources of bias	6-7
Study size	10	Explain how the study size was arrived at	6
Quantitative variables	11	Explain how quantitative variables were handled in the analyses. If applicable, describe which groupings were chosen and why	6-7
Statistical methods	12	(*a*) Describe all statistical methods, including those used to control for confounding	6-7
		(*b*) Describe any methods used to examine subgroups and interactions	6-7
		(*c*) Explain how missing data were addressed	6-7
		(*d*) *Cohort study*—If applicable, explain how loss to follow-up was addressed<br>*Case-control study*—If applicable, explain how matching of cases and controls was addressed	NA

For peer review only

		Cross-sectional study—If applicable, describe analytical methods taking account of sampling strategy	
		(*e*) Describe any sensitivity analyses	NA
**Results**			
Participants	13*	(a) Report numbers of individuals at each stage of study—eg numbers potentially eligible, examined for eligibility, confirmed eligible, included in the study, completing follow-up, and analysed	6
		(b) Give reasons for non-participation at each stage	NA
		(c) Consider use of a flow diagram	NA
Descriptive data	14*	(a) Give characteristics of study participants (eg demographic, clinical, social) and information on exposures and potential confounders	7
		(b) Indicate number of participants with missing data for each variable of interest	Table 1
		(c) *Cohort study*—Summarise follow-up time (eg, average and total amount)	NA: baseline analysis
Outcome data	15*	*Cohort study*—Report numbers of outcome events or summary measures over time	NA: baseline analysis
		Case-control study—Report numbers in each exposure category, or summary measures of exposure	
		Cross-sectional study—Report numbers of outcome events or summary measures	
Main results	16	(*a*) Give unadjusted estimates and, if applicable, confounder-adjusted estimates and their precision (eg, 95% confidence interval). Make clear which confounders were adjusted for and why they were included	7-9
		(*b*) Report category boundaries when continuous variables were categorized	7-9
		(*c*) If relevant, consider translating estimates of relative risk into absolute risk for a meaningful time period	NA
Other analyses	17	Report other analyses done—eg analyses of subgroups and interactions, and sensitivity analyses	7-9
**Discussion**			
Key results	18	Summarise key results with reference to study objectives	10
Limitations	19	Discuss limitations of the study, taking into account sources of potential bias or imprecision. Discuss both direction and magnitude of any potential bias	12-13
Interpretation	20	Give a cautious overall interpretation of results considering objectives, limitations, multiplicity of analyses, results from similar studies, and other relevant evidence	10-12
Generalisability	21	Discuss the generalisability (external validity) of the study results	10-12
**Other information**			
Funding	22	Give the source of funding and the role of the funders for the present study and, if applicable, for the original study on which the present article is based	2

*Give information separately for cases and controls in case-control studies and, if applicable, for exposed and unexposed groups in cohort and cross-sectional studies.
Note: An Explanation and Elaboration article discusses each checklist item and gives methodological background and published examples of transparent reporting. The STROBE checklist is best used in conjunction with this article (freely available on the Web sites of PLoS Medicine at http://www.plosmedicine.org/, Annals of Internal Medicine at http://www.annals.org/, and Epidemiology at http://www.epidem.com/). Information on the STROBE Initiative is available at www.strobe-statement.org.

For peer review only
